# The burden of prostate cancer in North Africa and Middle East, 1990–2019: Findings from the global burden of disease study

**DOI:** 10.3389/fonc.2022.961086

**Published:** 2022-09-13

**Authors:** Mohsen Abbasi-Kangevari, Sahar Saeedi Moghaddam, Seyyed-Hadi Ghamari, Mohammadreza Azangou-Khyavy, Mohammad-Reza Malekpour, Negar Rezaei, Nazila Rezaei, Ali-Asghar Kolahi, GBD 2019 NAME Prostate Cancer Collaborators, Behzad Abbasi, Erfan Amini, Ali H. Mokdad, Hamidreza Jamshidi, Mohsen Naghavi, Bagher Larijani, Farshad Farzadfar

**Affiliations:** ^1^ Non-Communicable Diseases Research Center, Endocrinology and Metabolism Population Sciences Institute, Tehran University of Medical Sciences, Tehran, Iran; ^2^ Endocrinology and Metabolism Research Center, Endocrinology and Metabolism Clinical Sciences Institute, Tehran University of Medical Sciences, Tehran, Iran; ^3^ Social Determinants of Health Research Center, Shahid Beheshti University of Medical Sciences, Tehran, Iran; ^4^ Department of Urology, Uro-oncology Research Center, Tehran University of Medical Sciences, Tehran, Iran; ^5^ Institute for Health Metrics and Evaluation, University of Washington, Seattle, WA, United States; ^6^ Department of Health Metrics Sciences, University of Washington, Seattle, WA, United States; ^7^ Department of Pharmacology, School of Medicine, Shahid Beheshti University of Medical Sciences, Tehran, Iran

**Keywords:** cancer, global burden of disease, incidence, mortality, prostate-specific antigen, prostatic neoplasms

## Abstract

**Background:**

Prostate cancer (PCa) is the second most prevalent cancer among men worldwide. This study presents estimates of PCa prevalence, incidence, death, years-of-life-lost (YLLs), years-lived-with-disability (YLDs), disability-adjusted-life-years (DALYs), and the burden attributable to smoking during 1990-2019 in North Africa and Middle East using data of Global Burden of Diseases (GBD) Study 2019.

**Methods:**

This study is a part of GBD 2019. Using vital registration and cancer registry data, the estimates on PCa burden were modeled. Risk factor analysis was performed through the six-step conceptual framework of Comparative Risk Assessment.

**Results:**

The age-standardized rates (95% UI) of PCa incidence, prevalence, and death in 2019 were 23.7 (18.5-27.9), 161.1 (126.6-187.6), and 11.7 (9.4-13.9) per 100,000 population. While PCa incidence and prevalence increased by 77% and 144% during 1990-2019, respectively, the death rate stagnated. Of the 397% increase in PCa new cases, 234% was due to a rise in the age-specific incidence rate, 79% due to population growth, and 84% due to population aging. The YLLs, YLDs, and DALYs of PCa increased by 2% (-11.8-23.1), 108% (75.5-155.1), and 6% (-8.9-28.1). The death rate and DALYs rate attributable to smoking have decreased 12% and 10%, respectively. The DALYs rate attributable to smoking was 37.4 (15.9-67.8) in Lebanon and 5.9 (2.5-10.6) in Saudi Arabia, which were the highest and lowest in the region, respectively.

**Conclusions:**

The PCa incidence and prevalence rates increased during 1990-2019; however, the death rate stagnated. The increase in the incidence was mostly due to the rise in the age-specific incidence rate, rather than population growth or aging. The burden of PCa attributable to smoking has decreased in the past 30 years.

## Introduction

Prostate cancer (PCa) is the second most prevalent cancer among men worldwide, accounting for 14.1% of all diagnosed cases with cancer in 2020 (1). PCa has the fifth rank among deaths due to neoplasm among men worldwide (2) and ranked among the top ten causes of disability-adjusted life years (DALYs) in men of older ages in 2019 (3). Smoking is the single risk factor for PCa, distinguished by the Global Burden of Disease (GBD) Study 2019 (4).

Globally, there are substantial variations in trends of PCa incidence and death (5). This could be attributable to regional population-based differences in risk factors, access to medical care, and disparate screening and diagnostic approaches across healthcare systems (6). Considering its burden (7), any change in screening, diagnosis, and management approaches could have substantial public health consequences (8). Therefore, investigation of the trends among various locations is essential in determining the accomplishments of distinct health policies and screening protocols across healthcare systems of countries. Providing policymakers with precise and reliable reports on trends and patterns of PCa empowers them to make prompt evidence-based decisions (9).

In this study, the North Africa and Middle East (NAME) super-region has been chosen to assess the burden of prostate cancer and its attributable risk factor. Despite similarities in culture and religion, the socio-economic status greatly differs across countries in the region. Currently, the countries in this region encounter numerous challenges in developmental aspects affecting their healthcare systems (10). Moreover, the lack of well-established national cancer registries challenges the accuracy of the burden estimates (11). Hence, studying the burden of disease trends in these countries using GBD as a tool could provide a more accurate picture of the current status in the health systems and efforts demanded to reduce PCa burden. Smoking is the only risk factor, for which there were enough high-quality evidence to be considered a risk factor for prostate cancer in GBD-2019 (3).

The objective of this study was to present estimates of PCa prevalence, incidence, death, years of life lost (YLLs), years lived with disability (YLDs), DALYs, and the burden attributable to smoking from 1990 to 2019 by age groups, and countries in NAME using the data of GBD-2019 (3).

## Materials and methods

### Data source

The data of GBD-2019 was used in the study, which includes estimations on epidemiological measures including prevalence, incidence, death, YLLs, YLDs, and DALYs for 286 causes of death, 369 diseases and injuries, and 87 risk factors in 204 countries and territories (3). NAME, a GBD super-region investigated in this study, included 21 countries: Afghanistan, Algeria, Bahrain, Egypt, Iran (Islamic Republic of), Iraq, Jordan, Kuwait, Lebanon, Libya, Morocco, Oman, Palestine, Qatar, Saudi Arabia, Sudan, Syrian Arab Republic, Tunisia, Turkey, United Arab Emirates, and Yemen (3). The data of PCa was extracted from GBD-2019: GBD code: B.1.18, International Statistical Classification of Diseases and Related Health Problems 10th Revision, World Health Organization (WHO) version 10 (ICD-10) code: C61-C61.9, D07.5, D29.1, D40.0 (for mapping death), and C61-C61.9, Z12.5, Z80.42, Z85.46 (for mapping new cases) (12, 13). The Global Health Data Exchange (GHDx) was used to extract the sources of data on PCa and its risk factor (14) (**Supplementary Table 1**). The detailed methods of GBD-2019 have been published previously (3). We briefly present the methods used to provide the estimates.

#### Death estimates

The data of the vital registration system and the cancer registry were transformed and modeled to provide the cause-specific death estimates using death-to-incidence ratios. Data were mapped to the GBD list of disease causes. To enhance the comparability of death data sources, the following statistical methods were used: 1) reclassification and redistribution of codes that are nonspecific or unspecific, 2) regression analysis using Bayesian geospatial regression software (CODEm, Cause of Death ensemble model), and 3) adjustment of the single cause death estimates applying CoDCorrect algorithm. As many as six selected covariates for CODEm models of PCa was defined including PCa summary exposure value (+1 direction), smoking prevalence (+1 direction), healthcare access and quality index (-1 direction), education *via* years per capita (-1 direction), lag distributed income per capita (-1 direction), and socio-demographic index (+1 direction). Finally, the single cause death estimates were adjusted by applying CoDCorrect algorithm. YLLs were calculated using normative global life expectancy and the number of deaths by age (3).

#### Incidence, prevalence, YLLs, YLDs, and DALYs estimates

To provide estimates on the incidence and prevalence of PCa, data were collected using the existing scientific reports on cohorts, registries, population surveys, and health system administrative data. The PCa prevalence was estimated for a maximum of 10 years after incidence. Prevalence extending beyond the 10-year period was only estimated for permanent sequelae resulting from prostatectomy. To estimate disability, the total PCa prevalence was split into four sequelae including diagnosis and primary therapy, controlled phase, metastatic phase, and the terminal phase. The diagnosis and primary therapy phase were defined as the time from the onset of symptoms to the end of treatment. The controlled phase was defined as the time between finishing primary treatment and the earliest of either: 1) cure (recurrence- and progression-free survival after 10 years), 2) death from another cause, 3) progression to the metastatic phase. The metastatic phase was defined as the time period of intensive treatment for metastatic disease. The terminal phase was defined as the one-month period prior to death. Considering the long-term disability due to either incontinence or impotence from prostatectomy, additional disability beyond these four sequelae was estimated for PCa. YLDs were calculated *via* multiplying the prevalence of each sequela by its disability weight and by adding the procedure-related morbidity associated with PCa treatment. YLLs due to PCa were calculated using normative global life expectancy, and the number of deaths by age. PCa DALYs were calculated by summing YLDs and YLLs. DisMod-MR 2.1, a Bayesian meta-regression modelling tool, was used to ensure consistency between incidence, prevalence, remission, excess mortality, and cause-specific mortality (3).

#### Decomposition investigation

The contribution of population growth, aging, and variations in age-specific incidence rates to the witnessed PCa new cases changes was investigated. In the first step, the age structure and age-specific incidence rate of PCa in 1990 were applied to the population size of 2019. In the second step, the age structure and age-specific incidence rate of PCa in 2019 was applied to the population size of 1990. The difference between the new cases as calculated in the first and second steps was considered as the contribution of changes in age structure from 1990 to 2019. The difference between the new cases in the first step and the actual new cases in 2019 was attributed to the population growth. The differences between the value of the second step and the actual new cases in 2019 was attributed to the changes in age-specific incidence rates (15).

#### Risk factor estimates

The six-step conceptual framework of Comparative Risk Assessment (CRA) was used to estimate the attributable burden of PCa (16). Data on attributable risk factors were derived from the World Cancer Research Fund Criteria since GBD 2010 (4). Smoking was the only risk factor, about which there were enough data to be included as a risk factor of PCa in GBD-2019. After calculating the relative risks for exposures, the levels of exposure based on various age, sex, location, and years were estimated. Attributable epidemiological measures of PCa due to smoking were computed by multiplication of Population Attributable Fractions (PAFs) and were reported in YLLs, YLDs, DALYs, and death rates (17).

### Data analysis

Age standardization was conducted using the direct method, applying a global age structure from the year 2019. Since no data were available for patients with PCa below the age of 15, data of patients aged 15 and higher were analyzed. Age-standardized rates of PCa for countries were calculated using the GBD world population standard and reported per 100,000 individuals. The 95% Uncertainty Intervals (95% UIs) were reported for each metric using 2.5% and 97.5% quintiles across 1,000 draws. The comparison regarding the differences in values of each metrics from 1990 to 2019 was computed to calculate the total and percentage of change. A regression model was used to investigate the association between Healthcare Access and Quality Index (HAQI) (18) and PCa burden. The statistical analyses and data visualizations were carried out using R statistical packages v3.4.3 (http://www.r-project.org, RRID: SCR_001905).

## Results

### Incidence, prevalence, and death

In 2019, there were 47,474 (95% UI 36,988-55,847) new cases of PCa, 348,924 (271,317–407,426) prevalent cases of PCa, and 19,089 (15,241–22,498) deaths due to PCa in NAME. The number of all-ages new cases, prevalent cases and deaths due to PCa has shown a 5.0-, 6.3-, and 2.9-fold increase since 1990, respectively (**Supplementary Table 2**).

The age-standardized incidence rate and prevalence of PCa in the region was 23.7 (18.5-27.9) and 161.1 (126.6-187.6) per 100,000 population. The age-standardized death rate of PCa in the region was 11.7 (9.4-13.9). The incidence rate of PCa ranged from 12.0 (8.5-17.0) in Egypt to 73.8 (47.9-103.4) in Lebanon. The prevalence of PCa ranged from 51.0 (38.8-64.7) in Afghanistan to 542.6 (362.0 to 751.2) in Lebanon. Simultaneously, the death rate of PCa has varied from 7.0 (5.1-9.7) in Egypt to 25.7 (16.0-37.7) in Lebanon (**Table 1**).

**Table 1:** Age-standardized rates (95% UI) for incidence, prevalence, and death due to prostate cancer in 1990 and 2019 and their percent changes.

Location		ASIR^1^ per 100,000 population			ASPR^2^ per 100,000 population			ASDR^3^ per 100,000 population		
		1990	2019	Percent changes	1990	2019	Percent changes	1990	2019	Percent changes
**North Africa and Middle East**		13.4 (10.8-16.4)	23.7 (18.5-27.9)	77.4 (51.1-117.2)	66.1 (54.4-77.5)	161.1 (126.6-187.6)	143.7 (108.1-190.3)	11.0 (8.9-13.9)	11.7(9.4-13.9)	6 (-8.6-28.7)
**Countries**	Afghanistan	11.5 (8.3-15.7)	13.5 (10.0-17.5)	17.4 (-10-49.4)	36.1 (28.5-46.2)	51 (38.8-64.7)	41.2 (6.5-81.7)	12.2 (8.6-16.9)	13.1(9.7-17.1)	7.1 (-17.4-36.1)
	Algeria	13.6 (10.2-17.6)	18.0 (13.4-25.0)	32.3 (-3.7-75.6)	69.7 (53.1-89.1)	123.4 (92.5-170.1)	77.1 (26.8-139.1)	11.3 (8.8-14.6)	9.7(7.4-13.3)	-13.7 (-33.8-11)
	Bahrain	23.1 (17.2-28.8)	33.8 (25.3-45.0)	46.5 (4.9-103.7)7	114.4 (88.6-141.6)	236.4 (178-319.2)	106.7 (45.1-190.7)	18.2 (13.5-22.5)	16.1(11.8-20.8)	-11.6 (-32.7-15.7)
	Egypt	7.2 (6.1-9.3)	12.0 (8.5-17.0)	66 (19.1-138.8)	33.3 (28.4-39.5)	73 (51.3-103)	119.4 (58.3-208.2)	6.5 (5.4-8.7)	7.0(5.1-9.7)	8.4 (-19.9-49.8)
	Iran (Islamic Republic of)	15.8 (12.2-19.6)	30.0 (21.3-34.7)	89.4 (50.9-142.5)	90.4 (70.8-110.4)	219.3 (158.3-257.6)	142.5 (93.1-207.9)	11.4 (9.0-14.4)	12.8(9.6-14.6)	12.2 (-6.7-40.8)
	Iraq	10.9 (7.7-15.2)	19.9 (14.9-26.3)	83.1 (32-159.6)	53 (38.6-71)	129.9 (97.2-166.9)	145.2 (75.9-248.7)	9.0 (6.4-12.8)	11.6(8.8-15.9)	28.7 (-4.7-74.8)
	Jordan	14.0 (10.9-17.9)	24.5 (17.1-32.4)	74.7 (27.5-137.6)	74.9 (59-94.8)	172.6 (121.6-230)	130.4 (67.5-218.8)	11.1 (8.7-14.0)	11.5(8.1-14.9)	4 (-22.1-37.9)
	Kuwait	16.1 (12.4-20.4)	31.2 (23.0-42.9)	94.2 (48.3-151.5)	119.7 (93.8-148.7)	247.4 (183.6-340.9)	106.7 (55.9-168.9)	7.6 (5.8-9.9)	10.2(7.5-13.5)	34.5 (8.7-66.2)
	Lebanon	30.4 (23.1-38.2)	73.8 (47.9-103.4)	142.8 (70.1-243.7)	156.1 (123.4-195.2)	542.6 (362-751.2)	247.6 (142.9-395.6)	22.8 (16.7-28.9)	25.7(16.0-37.7)	12.5 (-21.1-61.1)
	Libya	13.0 (8.2-17.6)	19.6 (14.2-26.1)	51 (2.1-139.1)	74.1 (49.3-97.6)	131.2 (95.2-173.1)	77.1 (19.9-171.8)	9.5 (6.2-13.1)	10.2(7.6-13.3)	6.7 (-24.2-64.4)
	Morocco	8.7 (6.3-11.4)	15.5 (11.1-20.2)	78.4 (27.8-135.3)	38.7 (30.5-47.3)	216.7 (170.2-289.9)	134.9 (69.9-207.6)	7.9 (5.6-10.7)	10.3(7.4-13.8)	30 (-2.5-69.1)
	Oman	15.8 (10.6-22.0)	29.6 (23.7-39.1)	87.7 (34.5-176.2)	90.3 (63.3-123.1)	197.9 (137.2-252.5)	140.1 (76.3-229.8)	11.5 (7.7-15.8)	12.5(10.0-16.0)	9.4 (-24-79.1)
	Palestine	23.7 (16.2-32.9)	31.5 (20.5-40.6)	32.7 (-6.4-103.8)	119.3 (85.2-162.9)	298.1 (207.8-420.3)	65.9 (18.4-147)	18.8 (12.7-26.5)	18.6(11.6-23.0)	-1.4 (-29.3-51.4)
	Qatar	19.6 (14.7-26.2)	42.3 (29.9-58.1)	116 (51.8-203.5)	103.4 (80.8-136.8)	176.2 (130.7-259.9)	188.2 (97.6-310.5)	15.1 (11.1-19.9)	19.2(13.7-26.0)	27 (-6-72.7)
	Saudi Arabia	12.9 (7.5-20.5)	23.4 (17.2-35.0)	82.1 (20-278.8)	70.4 (46.1-105.1)	77.2 (53.6-104.6)	150.2 (67.8-380.7)	10.4 (6.0-17.0)	9.0(6.8-13.4)	-14.1 (-39.5-73.3)
	Sudan	9.5 (6.6-13.2)	14.9 (10.4-19.9)	56.5 (17.8-116.1)	37.7 (28.4-50.2)	133.3 (93.8-208)	104.9 (53.7-178.4)	9.3 (6.5-13.0)	11.4(8.1-15.3)	22.3 (-4.4-64.5)
	Syrian Arab Republic	11.7 (7.8-18.8)	19.5 (13.3-31.4)	66.6 (9.9-166)	63.4 (46.5-89.9)	153.1 (108.9-215.8)	110.2 (39.8-225.3)	9.1 (5.7-15.4)	9.7(6.7-15.8)	6.3 (-26.9-68.4)
	Tunisia	11.6 (8.6-15.3)	21.3 (14.9-30.5)	82.9 (21.8-174.8)	65.6 (50-84.2)	249.4 (173.9-330.6)	133.4 (58.6-248.1)	8.8 (6.4-11.6)	9.1(6.6-12.7)	4 (-28.8-58)
	Turkey	19.6 (13.7-24.9)	35.1 (24.6-46.4)	78.5 (27.7-144.3)	94.6 (68.2-119.5)	161.4 (95.6-298.1)	163.5 (88.8-263.5)	16.2 (11.4-20.5)	14.7(10.5-18.7)	-9 (-32.3-21.1)
	United Arab Emirates	17.0 (10.3-29.4)	24.2 (13.7-46.9)	42.3 (-2-102.8)	93.3 (61.1-155)	72 (53.5-97.3)	72.9 (20.8-144.9)	12.9 (7.7-22.4)	12.8(7.4-24.7)	-0.8 (-29.2-37.3)
	Yemen	10.3 (6.5-14.7)	14.7 (10.8-19.6)	43.6 (5.9-110.8)	40.5 (28.9-55.8)	216.7 (170.2-289.9)	77.7 (32.4-155.3)	10.0 (6.1-14.5)	11.9(8.6-16.0)	19.7 (-10.4-72.1)

^1^Age-standardized incidence rate.

^2^Age-standardized prevalence rate.

^3^Age-standardized deaths rate.

The incidence rate and prevalence have shown an incessantly growing pattern since 1990, while the death rate has stagnated. The incidence rate, the prevalence rate, and death rate during 1990 to 2019 study increased by 77.4% (51.1-117.2), 143.7% (108.1-190.3), and 6% (-8.6-28.7). While the incidence rate increased in all countries in the region, the gap between the highest and lowest incidence rate among countries has widened during the past 30 years. The death rate increased in all countries except for six countries, including Saudi Arabia, Algeria, Bahrain, Turkey, Palestine, and United Arab Emirates (**Table 1** and **Supplementary Figures 1**, **2**).

**Figure 1:**
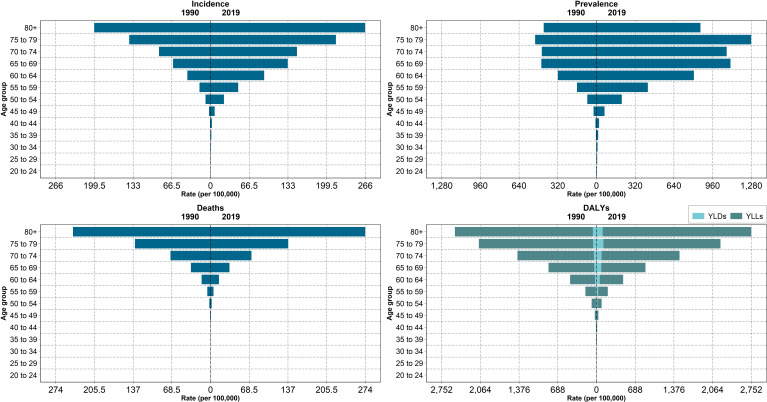
Rates of incidence, prevalence, deaths, YLLs, YLDs, and DALYs due to prostate cancer among age groups in North Africa and Middle East in 1990 and 2019.

Patients aged more than 70 accounted for 41.2% of all new and 29.8% of all prevalent cases. Moreover, 76.0% of deaths due to PCa were among patients aged more than 70 (**Figure 1** and **Supplementary Figure 3**). The incidence and mortality increased in both age groups under and over 70 years; however, the increase among people under 70 years of age was more prominent with the increasing rates of 210.3% and 132.3%, respectively.

The decomposition analysis showed that 234.2% of 397% increase in cases was due to a rise in the age-specific incidence rate. Population growth and population aging accounted for 78.9% and 83.9%, respectively (**Supplementary Table 3**). Higher HAQI was associated with higher PCa incidence (adjusted R^2^=0.51) and prevalence (adjusted R^2^=0.62) (**Supplementary Figure 4**).

### DALYs, YLLs, and YLDs

**Figure 2:**
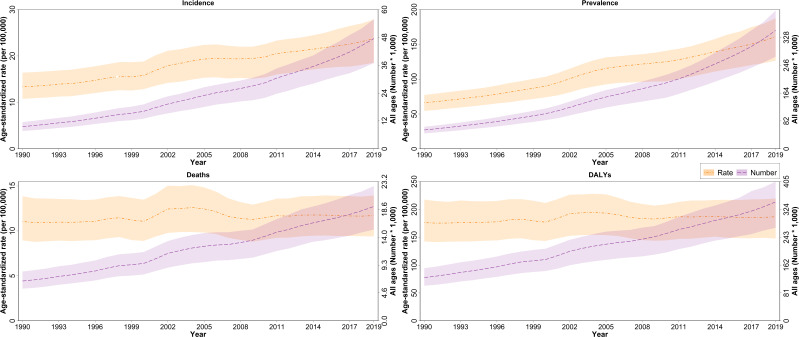
Age-standardized rates and all-ages numbers of incidence, prevalence, deaths, and DALYs due to prostate cancer in North Africa and Middle East from 1990 to 2019.

**Figure 3:**
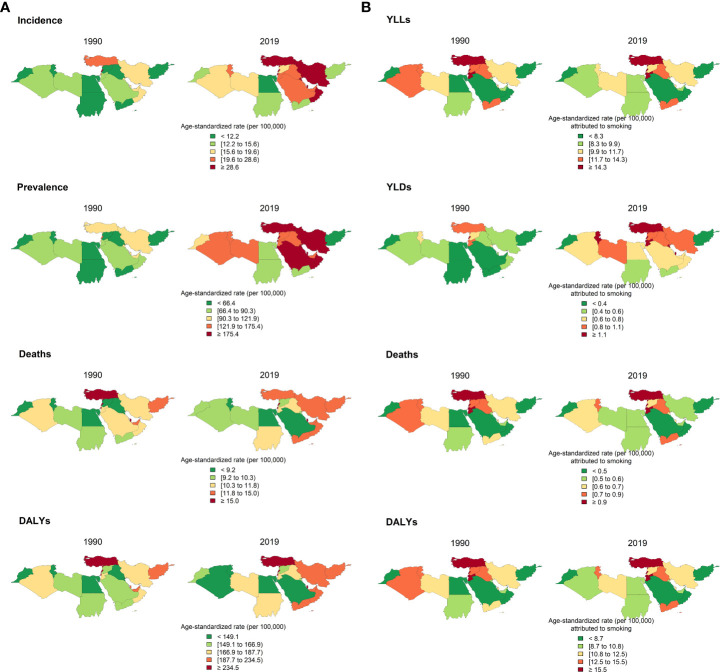
Distribution of epidemiological measures of prostate cancer by countries in 1900 and 2019: incidence, prevalence, death, and DALYs due to prostate cancer in 1990 and 2019 **(A)**; YLLs, YLDs, deaths, and DALYs of prostate cancer attributable to smoking in 1990 and 2019 **(B)**.

In 2019, the age-standardized DALYs due to PCa in NAME was 186.8 (147.7-219.5) per 100,000 population. The PCa DALYs increased by 6% (-8.9-28.1) since 1990. YLLs formed the highest proportion of DALYs both in 1990 and 2019 in all countries. DALYs' increasing trend is consistent with the pattern of YLLs and YLDs, with a 2.1% (-11.8-23.1) and 108.2% (75.5-155.1) increase since 1990. In addition, patients aged more than 70 accounted for 57.6% of all PCa DALYs. The DALYs of Algeria, Bahrain, Palestine, Saudi Arabia, and Turkey decreased (**Figures 2**, **3A**; **Table 2** and **Supplementary Table 4**). HAQI was not associated with PCa DALYs, YLLs, and YLDs (**Supplementary Figure 4**).

**Table 2:** Age-standardized YLLs, YLDs, and DALYs rate (95% UI) due to prostate cancer in 1990 and 2019 and their percent changes along with YLLs/DALYs and YLDs/DALYs.

Location		YLLs^1^ rate per 100,000 population			YLDs^2^ rate per 100,000 population			DALYs^3^ rate per 100,000 population			YLLs/DALYs		YLDs/DALYs	
		1990	2019	Percent changes	1990	2019	Percent changes	1990	2019	Percent changes	1990(%)	2019(%)	1990(%)	2019(%)
**North Africa and Middle East**		169.6 (137.3-209.6)	173.2 (137.9-203.6)	2.1 (-11.8-23.1)	6.5 (4.6-8.9)	13.6 (9.4-18.2)	108.2 (75.5-155.1)	176.2 (142.5-217.7)	186.8 (147.7-219.5)	6 (-8.9-28.1)	96.3	92.7	3.7	7.3
**Countries**	Afghanistan	192.7 (141.4-265.9)	203.6 (152.9-264.6)	5.6 (-18.7-34.9)	4.6 (3-6.6)	5.6 (3.7-8.1)	22.4 (-9.6-67.1)	197.3 (145–272)	209.2 (157.1-272.2)	6 (-18.4-35.3)	97.7	97.3	2.3	2.7
	Algeria	166 (124.8-214.7)	139 (103.7-189.6)	-16.3 (-37.4-10.8)	6.6 (4.3-9.3)	10 (6.6-14.8)	51.4 (5.3-110)	172.6 (130-222.2)	149 (111.1-203.2)	-13.7 (-35.4-14.2)	96.2	93.3	3.8	6.7
	Bahrain	268.2 (199.4-331.4)	220 (166.4-284)	-18 (-39.6-8.8)	11.6 (8-16.4)	19.2 (12.6-28)	65.7 (15.1-133.6)	279.8 (209.4-345)	239.3 (181.3-310.2)	-14.5 (-36.9-14.1)	95.9	91.9	4.1	8.1
	Egypt	101.5 (86.4-130.6)	112 (80.3-156.3)	10.3 (-19.9-55.6)	3.6 (2.5-4.9)	7.1 (4.4-10.9)	96.1 (31.6-181.1)	105.1 (89.5-135.2)	119.1 (85.7-166)	13.2 (-17.3-60.3)	96.6	94.0	3.4	6.0
	Iran (Islamic Republic of)	173.7 (136.4-216.9)	190.5 (142.2-215.3)	9.7 (-8.6-36.3)	8.2 (5.5-11.4)	17.5 (11.6-24.2)	114.1 (68.8-173.2)	181.9 (142.8-227.3)	208.1 (155.2-235.7)	14.4 (-4.4-41.6)	95.5	91.6	4.5	8.4
	Iraq	138.4 (97.7-197.5)	172.6 (130-230.2)	24.7 (-9.3-73.5)	5.3 (3.4-7.9)	10.7 (6.8-15.5)	100.2 (38.6-195.3)	143.7 (101.4-205.6)	183.3 (138.2-241.9)	27.5 (-7.4-78.2)	96.3	94.2	3.7	5.8
	Jordan	167.6 (130.4-212.4)	166.9 (117-214.4)	-0.4 (-25.3-33.1)	7.1 (4.8-9.9)	14.2 (8.6-20.8)	100.3 (44-182.6)	174.7 (136.4-221.3)	181.1 (126.8-234.8)	3.7 (-22.5-39.4)	95.9	92.2	4.1	7.8
	Kuwait	119.8 (91.2-153.3)	146 (107.5-193.1)	21.8 (-2.5-51.9)	9.5 (6.2-13.7)	18.8 (12.1-27.5)	96.6 (45.4-163.2)	129.3 (99-164.7)	164.7 (121.4-218.9)	27.3 (1.6-58.4)	92.7	88.6	7.3	11.4
	Lebanon	334.9 (250.7-419.2)	371.9 (235.2-528.3)	11.1 (-21.5-55.1)	15.6 (10.6-22.1)	44.6 (26.7-68.4)	185.6 (97.5-303.1)	350.5 (263.9-436.7)	416.5 (266.5-591.2)	18.8 (-15.7-66)	95.5	89.3	4.5	10.7
	Libya	148.6 (95.5-201.3)	158.7 (117.9-207)	6.9 (-24.7-64.8)	6.7 (3.9-10)	11.3 (7.3-16.4)	67.6 (11.1-165.2)	155.3 (100-210.9)	170 (126.1-221.8)	9.5 (-22.9-68.9)	95.7	93.4	4.3	6.6
	Morocco	121.1 (89.2-156.4)	155.9 (113.9-204.8)	28.7 (-4.9-66.8)	4 (2.7-5.5)	8.1 (5.2-11.5)	105.3 (44.7-183.6)	125.1 (92-161.3)	164 (120.4-215.2)	31.1 (-3.1-70.1)	96.8	95.1	3.2	4.9
	Oman	173.6 (115.4-243.6)	177.2 (143.9-226.5)	2.1 (-27.6-61.6)	8.5 (5.2-12.5)	18 (12.5-25.7)	111.3 (48.7-206)	182.1 (121.9-255.4)	195.2 (158.5-248.1)	7.2 (-23.5-68)	95.3	90.8	4.7	9.2
	Palestine	278.8 (189.9-388)	265.3 (172.6-335.9)	-4.9 (-32.5-46.3)	11.8 (7.3-18.5)	17 (10.5-24)	43.8 (1.3-122.5)	290.6 (197.5-402.6)	282.3 (184.4-358.8)	-2.9 (-31.3-49.7)	95.9	94.0	4.1	6.0
	Qatar	216.7 (161.7-286.6)	242.6 (175.1-329.2)	12 (-18.2-59.3)	10 (6.7-14.2)	24.3 (15.5-34.9)	142.9 (67.5-255.6)	226.7 (170.2-300.2)	266.9 (193.1-362.4)	17.8 (-14.4-66.5)	95.6	90.9	4.4	9.1
	Saudi Arabia	158.5 (94.7-251)	134.3 (101.9-200.9)	-15.2 (-41.7-74.5)	6.3 (3.4-10.2)	14 (9.1-21.9)	122 (45.9-353)	164.8 (98.8-260.3)	148.3 (111.7-222.3)	-10 (-38.1-85.3)	96.2	90.6	3.8	9.4
	Sudan	145.1 (102.7-201.2)	172.8 (120.7-233.4)	19.2 (-8.9-61.8)	4.2 (2.7-6.5)	7.4 (4.6-11)	75.2 (25.3-147.6)	149.3 (105.9-208.2)	180.2 (124.7-243.4)	20.7 (-7.9-64.3)	97.2	95.9	2.8	4.1
	Syrian Arab Republic	145.3 (94.8-233)	147.6 (101.2-237)	1.6 (-31.1-58.8)	6.1 (3.7-9.7)	11.4 (6.8-18.7)	88.7 (20.6-205.9)	151.4 (99.1-242.8)	159 (109.8-254.7)	5 (-28.7-64.3)	96.0	92.8	4.0	7.2
	Tunisia	128.9 (95.6-169.9)	134.2 (95.8-188.4)	4.1 (-29.3-57.1)	6 (3.8-8.6)	12.7 (7.9-19.2)	111.2 (42.3-217.1)	134.9 (99.7-177.9)	146.8 (104.5-208.2)	8.8 (-25.7-64.2)	95.6	91.3	4.4	8.7
	Turkey	255.4 (181.6-321.2)	215.5 (153.2-276.3)	-15.6 (-38.2-12.8)	9.6 (6.1-14.3)	20.9 (13.1-30.3)	116.8 (52.9-203.7)	265 (189-334.9)	236.4 (168.7-303.5)	-10.8 (-34.7-20)	96.4	91.2	3.6	8.8
	United Arab Emirates	204.9 (122.9-354.7)	200.9 (116.4-387.1)	-1.9 (-31.5-36.7)	8.8 (4.9-16)	14.1 (7.1-28.5)	59.8 (8.4-131)	213.7 (128.3-368.3)	215 (125.1-415.3)	0.6 (-29.3-39.5)	95.9	93.4	4.1	6.6
	Yemen	155.4 (98.8-224.5)	183.4 (133.9-244.4)	18 (-12.1-72)	4.6 (2.7-7.1)	7.2 (4.6-10.7)	56.8 (11.7-136.5)	160 (101.4-232.2)	190.6 (139.4-254)	19.1 (-11.4-73.7)	97.1	96.2	2.9	3.8

^1^Years of Life Lost.

^2^Years Lived with Disability.

^3^Disability-Adjusted Life Years.

### The burden of PCa attributable to smoking

The age-standardized DALYs rate and death rate attributable to smoking in NAME in 2019 was 12.8 (5.7-20.5) and 0.7 (0.3-1.2) per 100,000 population. The DALYs rate attributable to smoking varied significantly among countries, ranging from 5.9 (2.5-10.6) in Saudi Arabia to 37.4 (15.9-67.8) in Lebanon. Death rate attributable to smoking ranged from 0.3 (0.1-0.6) in Saudi Arabia to 2.1 (0.9-3.9) in Lebanon. The DALYs rate and death rate attributable to smoking in the region has decreased 10% (-28.5-15.2) and 11.9% (-30.4-12.4), respectively. While Turkey had the highest DALYs attributable to smoking in 1990, it managed to decrease it by almost 33% in 2019. In contrast, Lebanon with the second highest DALYs attributable to smoking in 1990 witnessed a 37% increase in 2019 (**Figure 3B**; **Table 3** and **Supplementary Table 5**).

**Table 3:** Age-standardized rates (95% UI) for YLLs, YLDs, and DALYs of prostate cancer attributable to smoking in 1990 and 2019 and their percent changes.

Location		YLLs^1^ rate per 100,000 population			YLDs^2^ rate per 100,000 population			DALYs^3^ rate per 100,000 population			Death rate per 100,000 population		
		1990	2019	Percent changes	1990	2019	Percent changes	1990	2019	Percent changes	1990	2019	Percent changes
**North Africa and Middle East**		13.7 (5.8-21.7)	11.8 (5.3-18.8)	-13.8 (-31.5-9.8)	0.6 (0.2-1)	1 (0.4-1.7)	83.9 (44.5-135.1)	14.2 (6-22.6)	12.8 (5.7-20.5)	-10 (-28.5-15.2)	0.8 (0.3-1.3)	0.7 (0.3-1.2)	-11.9 (-30.4-12.4)
**Countries**	Afghanistan	5.4 (2.1-10)	7.8 (3.1-13.6)	44.2 (2.6-102.2)	0.1 (0-0.2)	0.2 (0.1-0.4)	71.3 (18.2-160.5)	5.6 (2.1-10.2)	8 (3.2-14)	44.8 (3.4-103.1)	0.3 (0.1-0.6)	0.5 (0.2-0.8)	39.9 (1.4-97.7)
	Algeria	13.5 (5.8-22.6)	9.9 (4.3-16.8)	-27 (-48.6-3.8)	0.5 (0.2-0.9)	0.7 (0.3-1.3)	33.6 (-10.1-91.3)	14 (6.1-23.4)	10.6 (4.7-18)	-24.7 (-46.7-7.1)	0.9 (0.4-1.5)	0.7 (0.3-1.1)	-24.9 (-46.8-2.7)
	Bahrain	18.9 (8-31.3)	12.8 (5.1-22.6)	-32 (-52.6–3.9)	0.8 (0.3-1.5)	1.1 (0.4-2.1)	36.1 (-11.1-100.6)	19.7 (8.3-32.7)	14 (5.6-24.4)	-29.1 (-50.5-0.7)	1.2 (0.5-2)	0.9 (0.4-1.6)	-26 (-47.3-2.8)
	Egypt	7.5 (3.3-11.7)	9.3 (4.1-16.1)	24.2 (-13.4-76.6)	0.3 (0.1-0.5)	0.6 (0.3-1.1)	123.4 (47.2-230.4)	7.7 (3.5-12.1)	9.9 (4.5-17.1)	27.8 (-11-81.8)	0.4 (0.2-0.7)	0.6 (0.2-1)	23.4 (-12.9-73.9)
	Iran (Islamic Republic of)	10.6 (4.3-17.2)	10.1 (4.5-16.6)	-5.3 (-28-26.3)	0.6 (0.2-1)	1.1 (0.4-1.8)	92.3 (43.9-157.7)	11.2 (4.6-18.1)	11.1 (5-18.4)	-0.4 (-23.5-32.6)	0.6 (0.3-1)	0.6 (0.3-1)	-3.5 (-26.8-29.7)
	Iraq	13.4 (5.3-22.1)	13.7 (5.7-22.7)	3 (-27.8-48.8)	0.5 (0.2-0.9)	0.9 (0.4-1.6)	66.2 (11.6-158.5)	13.9 (5.6-23)	14.6 (6-24.4)	5.4 (-26.3-52.2)	0.8 (0.3-1.4)	0.9 (0.4-1.5)	7.6 (-23.1-52.7)
	Jordan	18.1 (7.7-30.1)	14.4 (6.5-24.4)	-20.4 (-48.1-22.1)	0.8 (0.3-1.4)	1.3 (0.5-2.4)	66.4 (7.8-158.7)	18.9 (8-31.5)	15.8 (7.2-26.6)	-16.7 (-46.2-27.7)	1.1 (0.5-1.8)	0.9 (0.4-1.5)	-18.2 (-45.9-23.5)
	Kuwait	9.3 (3.8-15.6)	9 (4.1-15.6)	-3.2 (-29.2-31.1)	0.8 (0.3-1.4)	1.2 (0.5-2.2)	56.7 (10.6-125.9)	10.1 (4.1-17)	10.2 (4.5-17.7)	1.5 (-25.6-37.5)	0.6 (0.2-0.9)	0.6 (0.3-1)	8.4 (-21.9-45.8)
	Lebanon	26 (11.3-43.7)	33.1 (14-59.9)	27.4 (-13.9-87.4)	1.3 (0.5-2.2)	4.3 (1.7-8.1)	233 (118.9-404.7)	27.3 (11.7-45.8)	37.4 (15.9-67.8)	37.1 (-6.7-103.1)	1.6 (0.7-2.8)	2.1 (0.9-3.9)	29.4 (-13.1-93.7)
	Libya	11.5 (4.5-19.6)	10.3 (4.5-16.9)	-10.6 (-40.5-42.5)	0.6 (0.2-1)	0.8 (0.3-1.5)	46 (-8.6-147.3)	12.1 (4.7-20.6)	11.1 (4.8-18.2)	-7.9 (-38.9-47.3)	0.7 (0.3-1.1)	0.6 (0.2-1)	-11.7 (-40.3-42.1)
	Morocco	7.4 (3.2-12.1)	7.1 (2.9-11.9)	-3.8 (-33.9-32.5)	0.3 (0.1-0.4)	0.4 (0.2-0.7)	59.7 (6.3-132.6)	7.7 (3.3-12.6)	7.5 (3-12.6)	-1.7 (-32.7-35.2)	0.4 (0.2-0.7)	0.4 (0.2-0.7)	-2.2 (-32.1-36.1)
	Oman	8.2 (3.2-14)	5.8 (2.3-9.7)	-29.4 (-51.5-12.7)	0.4 (0.2-0.8)	0.7 (0.3-1.2)	48.8 (0.5-128.7)	8.7 (3.3-14.8)	6.5 (2.6-10.8)	-25.5 (-48.6-17.6)	0.5 (0.2-0.8)	0.4 (0.2-0.7)	-23.3 (-48.6-29.5)
	Palestine	23.2 (9.5-42.1)	20.5 (8.9-34.4)	-11.8 (-40.8-39.1)	1.1 (0.4-2)	1.4 (0.6-2.5)	36.3 (-8.2-119.4)	24.3 (9.9-43.8)	21.9 (9.4-36.6)	-9.7 (-39.2-42.2)	1.4 (0.6-2.6)	1.3 (0.6-2.2)	-8.9 (-38.9-43.9)
	Qatar	10.7 (4-19.3)	10.4 (3.9-18.5)	-3.4 (-35.2-43.9)	0.5 (0.2-1)	1.2 (0.5-2.3)	121 (44.1-240.4)	11.2 (4.2-20.3)	11.5 (4.4-20.7)	2.4 (-31.6-52.4)	0.7 (0.2-1.2)	0.7 (0.3-1.3)	8.4 (-26.9-58.2)
	Saudi Arabia	5.6 (1.5-11.5)	5.3 (2.2-9.5)	-5.7 (-45.4-124.9)	0.2 (0.1-0.5)	0.6 (0.3-1.2)	166.7 (49.2-535.9)	5.8 (1.6-11.9)	5.9 (2.5-10.6)	1.4 (-41.2-141.5)	0.3 (0.1-0.7)	0.3 (0.1-0.6)	-7.7 (-46.7-121.7)
	Sudan	8.8 (2.9-16.2)	9.7 (3.6-18.2)	11.3 (-23.3-62.4)	0.3 (0.1-0.5)	0.4 (0.2-0.9)	67.9 (8.7-155.9)	9 (3.1-16.8)	10.2 (3.7-19)	12.9 (-21.6-64.6)	0.5 (0.2-1)	0.6 (0.2-1.1)	14.9 (-18.4-66)
	Syrian Arab Republic	13.9 (5.4-24.9)	11.1 (4.6-20.3)	-19.9 (-49.7-35.2)	0.6 (0.2-1.1)	0.9 (0.4-1.8)	55.3 (-6-156.4)	14.5 (5.7-25.9)	12.1 (5-22)	-16.8 (-47.3-40.2)	0.8 (0.3-1.5)	0.7 (0.3-1.3)	-17.1 (-47.2-42.2)
	Tunisia	12.1 (4.9-20)	11.5 (4.8-20.2)	-5 (-38.5-49)	0.6 (0.2-1)	1.1 (0.4-2.1)	94.9 (21.9-205.3)	12.7 (5.1-21)	12.7 (5.3-22.2)	-0.4 (-35.6-55.4)	0.8 (0.3-1.3)	0.7 (0.3-1.3)	-4.9 (-38.2-49.7)
	Turkey	27.9 (11.6-46.3)	17.6 (7.7-29.4)	-36.8 (-56.9–9.6)	1.1 (0.4-2)	1.9 (0.8-3.4)	71.9 (15.3-154.5)	29 (12.1-48)	19.6 (8.5-32.7)	-32.6 (-53.9–3.1)	1.6 (0.7-2.7)	1.1 (0.5-1.8)	-33.5 (-54.3–4.2)
	United Arab Emirates	9.5 (3.8-19.3)	9.2 (3.4-21.3)	-2.9 (-37.8-40.4)	0.4 (0.2-0.9)	0.7 (0.2-1.6)	59.9 (0-146.3)	9.9 (4-20.1)	9.9 (3.6-22.9)	-0.2 (-36-45.4)	0.6 (0.2-1.2)	0.6 (0.2-1.3)	-1.3 (-36-42.6)
	Yemen	11.8 (4.5-20.8)	13 (5.2-21.9)	10.2 (-21.1-65.5)	0.4 (0.1-0.7)	0.5 (0.2-1)	49.7 (2.1-128.2)	12.1 (4.6-21.5)	13.5 (5.4-22.9)	11.4 (-20.2-66.6)	0.7 (0.3-1.2)	0.8 (0.3-1.3)	11.5 (-19.5-65.7)

^1^Years of Life Lost.

^2^Years Lived with Disability.

^3^Disability-Adjusted Life Years.

## Discussion

The PCa incidence and prevalence incessantly increased, while the death rate merely stagnated. The increase in the incidence was majorly due to a rise in the age-specific incidence rate. While YLLs and DALYs of PCa slightly increased, YLDs was doubled from 1990 to 2019. The PCa death and DALYs rate attributable to smoking decreased.

The steady increasing trends in death rates, despite the sharp increases in incidence and prevalence, could be associated with early detection *via* screening, or recent advances in surgical and non-surgical therapeutic approaches (19). However, compared with western regions, the burden of PCa is still low (3), which could be due to several factors ranging from lack of proper population-based registries to genetic differences and diet (20).

The burden of PCa varied significantly among the countries, which could be due to limited awareness and variable coverage of screenings in the region, along with the divergent development of cancer registries (21). There was a six-fold gap between Algeria, with the lowest PCa incidence, and Lebanon, which had the highest. In this regard, the difference between Lebanon and Algeria could partly be due to their healthcare system approaches towards the widespread use of prostate-specific antigen (PSA) screening tests (22, 23). This study's findings are consistent with a previous study on Lebanese national cancer registry. In this country, age structure, and prevalence of genetic and lifestyle risk factors might be other explanations in addition to improved screenings and diagnostics (24).

Controversies over the role of PSA testing in overdiagnosis (2) and reducing disease-specific mortality (25) have casted doubt on its liability to be a national screening strategy. Nevertheless, it has been used among many populations. Early detection by screenings needs updated healthcare systems and resources. Since there is limited data on the prevalence of PSA screening among countries in the region, further research is needed on the optimal strategies for early detection and prompt medical care. Moreover, the PSA screening utilization pattern among various groups needs to be investigated.

Patients aged>70 accounted for some 60% of all DALYs due to PCa, consistent with previous studies (26, 27). Most of the increase in incidence was attributable to a rise in the incidence rate. Although the incidence rates increased in all age-groups, they increased more among patients aged<70, resulting in higher expected mortality in the future.

Despite increased age-standardized incidence and prevalence, the death rate decreased in Saudi Arabia, Turkey, and United Arab Emirates. This finding might be reflecting the better management of PCa due to timely and efficient interventions which needs to be furthered studied and used as role models for the rest of the countries.

There were significant heterogeneities among countries in the burden of PCa attributable to smoking, which could be traced back to the time trends of smoking prevalence. The prevalence of smoking in Lebanon, the country with the highest DALYs for PCa attributable to smoking, has stagnated in recent decades (28). In comparison, the prevalence of smoking in Turkey, which was the most prosperous country in decreasing the burden of PCa attributable to smoking, has significantly decreased in the recent decades (29). Turkey remains as a country that has one of the highest attributable burdens to smoking that might also explain the higher burden of prostate cancer in this country. Countries need to reconsider their smoking elimination policies to keep up with the smoking reduction agenda of Sustainable Development Goals (30). Interestingly, in countries with decreased affordability of cigarettes such as Egypt and Turkey (31), the trend of attributable burden of PCa to smoking is not similar. Although smoking is the only risk factor recognized by GBD-2019, the burden attributable to this factor does not solely explain the PCa burden. Hence, other potential risk factors such as age (32), ethnicity (33), genetic factors (34), diet (35), hormone levels (36), and obesity (37) need to be investigated in future research.

HAQI was associated with PCa incidence and prevalence. In contrast, there were no associations between HAQI and DALYs, YLLs, and YLDs. The higher PCa incidence and prevalence among countries with higher HAQI could be due to more advanced screening and detection systems, especially considering the highly variable approaches towards management of PCa burden in the super-region (38). Nevertheless, the role of HAQI in the burden of PCa needs to be further assessed in future studies.

## Strengths and limitations

This study provides the opportunity to investigate the current situation and time trends of health metrics and measures in the region during the past three decades. Nevertheless, the overall quality of GBD estimates fundamentally relies on the accuracy of data sources used in the modeling. The reliability of registry data among countries with limited resources in this region with dynamic population due to immigrations and changes in ethnicities could be questionable. Thus, the GBD includes various modeling processes to overcome this limitation and presents metrics with 95% UIs. Due to insufficient evidence, smoking was the only attributable risk factor for PCa recognized by GBD-2019, which calls for further research to investigate other possible risk factors towards capturing a clearer picture of the PCa burden. Furthermore, the stage and histopathology of PCa which are determinatives of its prognosis were not considered in this study.

## Conclusions

PCa incidence and prevalence increased from 1990 to 2019; however, the death rate had a steadier pattern. The increase in the incidence was mostly due to the rise in the age-specific incidence rate, rather than population growth or aging. The burden of PCa attributable to smoking has decreased in the past 30 years.

## Data availability statement

Publicly available datasets were analyzed in this study. This data can be found here: https://vizhub.healthdata.org/gbd-results/.

## Ethics statement

The studies involving human participants were reviewed and approved by institutional review board of Endocrinology and Metabolism Research Institute at Tehran University of Medical Sciences (IR.TUMS.EMRI.REC.1400.028). Written informed consent for participation was not required for this study in accordance with the national legislation and the institutional requirements.

## GBD 2019 NAME Prostate Cancer Collaborators

Behzad Abbasi, Hassan Abidi, Eman Abu-Gharbieh, Muhammad Sohail Afzal, Araz Ramazan Ahmad, Sajjad Ahmad, Ali Ahmadi, Sepideh Ahmadi, Haroon Ahmed, Mostafa Akbarzadeh-Khiavi, Hamed Akhavizadegan, Hanadi Al Hamad, Fadwa Alhalaiqa Naji Alhalaiqa, Yousef Alimohamadi, Syed Mohamed Aljunid, Omar Almidani, Jalal Arabloo, Morteza Arab-Zozani, Seyyed Shamsadin Athari, Sina Azadnajafabad, Amirhossein Azari Jafari, Nayereh Baghcheghi, Nader Bagheri, Sara Bagherieh, Abdul-Monim Mohammad Batiha, Akshaya Srikanth Bhagavathula, Ali Bijani, Nadeem Shafique Butt, Reza Darvishi Cheshmeh Soltani, Ahmad Daryani, Mostafa Dianatinasab, Iman El Sayed, Muhammed Elhadi, Ali Fatehizadeh, Masood Fereidoonnezhad, Masoud Foroutan, Maryam Gholamalizadeh, Pouya Goleij, Mohamad Golitaleb, Mohammed Ibrahim Mohialdeen Gubari, Nima Hafezi-Nejad, Arvin Haj-Mirzaian, Samer Hamidi, Shafiul Haque, Khezar Hayat, Mohammad-Salar Hosseini, Mowafa Househ, Elham Jamshidi, Amirreza Javadi Mamaghani, Farahnaz Joukar, Ali Kabir, Rohollah Kalhor, Amirali Karimi, Yousef Saleh Khader, Javad Khanali, Behzad Kiani, Hamid Reza Koohestani, Somayeh Livani, Farzan Madadizadeh, Ahmad R Mafi, Ata Mahmoodpoor, Keivan Majidzadeh-A, Reza Malekzadeh, Ahmad Azam Malik, Fariborz Mansour-Ghanaei, Seyed Farzad Maroufi, Entezar Mehrabi Nasab, Seyyedmohammadsadeq Mirmoeeni, Yousef Mohammad, Esmaeil Mohammadi, Saeed Mohammadi, Abdollah Mohammadian-Hafshejani, Sara Momtazmanesh, Rahmatollah Moradzadeh, Paula Moraga, Mohammadreza Naghipour, Zuhair S Natto, Seyed Aria Nejadghaderi, Maryam Noori, Ali Nowroozi, Hassan Okati-Aliabad, Reza Pakzad, Zahra Zahid Piracha, Faheem Hyder Pottoo, Alireza Rafiei, Vahid Rahmanian, Mahsa Rashidi, Mohammad-Mahdi Rashidi, Mohammad Sadegh Razeghinia, Mohsen Rezaeian, Umar Saeed, Maryam Sahebazzamani, Amirhossein Sahebkar, Abdallah M Samy, Muhammad Arif Nadeem Saqib, Brijesh Sathian, Sadaf G Sepanlou, Saeed Shahabi, Masood Ali Shaikh, Sara Sheikhbahaei, Reza Shirkoohi, Parnian Shobeiri, Muhammad Suleman, Amir Tiyuri, Irfan Ullah, Faezeh Vakhshiteh, Sahel Valadan Tahbaz, Seyed Hossein Yahyazadeh Jabbari, Fereshteh Yazdanpanah, Deniz Yuce, Mazyar Zahir, Maryam Zamanian, Iman Zare, Mohammad Zoladl.

## GBD 2019 NAME Prostate Cancer Collaborators Affiliations

Social Determinants of Health Research Center (J Khanali MD, M Rashidi MD), Department of Epidemiology (A Ahmadi PhD), School of Advanced Technologies in Medicine (S Ahmadi PhD), Cancer Research Center (M Gholamalizadeh PhD), Department of Pharmacology (A Haj-Mirzaian MD, Prof H Jamshidi PhD), Obesity Research Center (A Haj-Mirzaian MD), Department of Clinical Oncology (A R Mafi MD), School of Medicine (S Nejadghaderi MD), Shahid Beheshti University of Medical Sciences, Tehran, Iran; Non-Communicable Disease Research Center, Endocrinology and Metabolism Population Sciences Institute (S Azadnajafabad MD, J Khanali MD, S Maroufi MD, E Mohammadi MD, S Momtazmanesh MD, S Nejadghaderi MD, M Rashidi MD, P Shobeiri MD), Uro-oncology Research Center (B Abbasi MD), Department of Urology (H Akhavizadegan MD), Department of Epidemiology and Biostatistics (Y Alimohamadi PhD), School of Medicine (N Hafezi-Nejad MD, A Karimi MD, S Momtazmanesh MD, A Nowroozi B.Med.Sc.), Digestive Diseases Research Institute (Prof R Malekzadeh MD, S G Sepanlou MD), Faculty of Medicine (S Maroufi MD, E Mohammadi MD, P Shobeiri MD), Tehran Heart Center (E Mehrabi Nasab MD), Cancer Research Center (R Shirkoohi PhD), Cancer Biology Research Center (R Shirkoohi PhD), Nanotechnology Research Center (F Vakhshiteh PhD), Department of Pediatric Allergy and Immunology (F Yazdanpanah MD), Department of Pharmacology, School of Medicine (M Zahir MD), Tehran University of Medical Sciences, Tehran, Iran; Reproductive Biomedicine Research Center (B Abbasi MD), Royan Institution, Isfahan, Iran; Laboratory Technology Sciences Department (H Abidi PhD), Nursing (M Zoladl PhD), Yasuj University of Medical Sciences, Yasuj, Iran; Clinical Sciences Department (E Abu-Gharbieh PhD), University of Sharjah, Sharjah, United Arab Emirates; Department of Life Sciences (M S Afzal PhD), School of Sciences (M N Saqib PhD), University of Management and Technology, Lahore, Pakistan; College of Nursing (A R Ahmad PhD), International Relations & Diplomacy, Ranya - Al Sulaimaniyah, Iraq; International Relations & Diplomacy (A R Ahmad PhD), Tishk International University, Erbil, Iraq; Department of Health and Biological Sciences (S Ahmad PhD), Abasyn University, Peshawar, Pakistan; Department of Epidemiology and Biostatistics (A Ahmadi PhD, A Mohammadian-Hafshejani PhD), Basic Health Sciences Institute (N Bagheri PhD), Shahrekord University of Medical Sciences, Shahrekord, Iran; Department of Biosciences (H Ahmed PhD), COMSATS Institute of Information Technology, Islamabad, Pakistan; Liver and Gastrointestinal Diseases Research Center (M Akbarzadeh-Khiavi PhD), Student Research Committee (M Hosseini MD), Department of Parasitology (A Javadi Mamaghani PhD), Anesthesiology and Critical Care (Prof A Mahmoodpoor MD), Department of Pediatric Allergy and Immunology (F Yazdanpanah MD), Tabriz University of Medical Sciences, Tabriz, Iran; Geriatric and Long Term Care Department (H Al Hamad MD, B Sathian PhD), Rumailah Hospital (H Al Hamad MD), Hamad Medical Corporation, Doha, Qatar; Faculty of Nursing (F A N Alhalaiqa PhD, Prof A M Batiha PhD), Philadelphia University, Amman, Jordan; Psychological Sciences Association, Amman, Jordan (F A N Alhalaiqa PhD); Pars Hospital (Y Alimohamadi PhD), Health Management and Economics Research Center (J Arabloo PhD), Minimally Invasive Surgery Research Center (A Kabir MD), Student Research Committee (M Noori MD), Epidemiology (A Tiyuri MSc), Iran University of Medical Sciences, Tehran, Iran; Department of Health Policy and Management (Prof S M Aljunid PhD), Kuwait University, Kuwait, Kuwait; International Centre for Casemix and Clinical Coding (Prof S M Aljunid PhD), National University of Malaysia, Bandar Tun Razak, Malaysia; Department of Urology (O Almidani MD), Cleveland Clinic Abu Dhabi, Abu Dhabi, United Arab Emirates; Nuffield Department of Surgical Sciences (O Almidani MD), University of Oxford, Oxford, Oxford, UK; Social Determinants of Health Research Center (M Arab-Zozani PhD), Department of Epidemiology (A Tiyuri MSc), Birjand University of Medical Sciences, Birjand, Iran; Department of Immunology (S Athari PhD), Zanjan University of Medical Sciences, Zanjan, Iran; School of Medicine (A Azari Jafari MD, S Mirmoeeni MD), Shahroud University of Medical Sciences, Shahroud, Iran; Department of Nursing (N Baghcheghi PhD), Social Determinants of Health Research Center (H Koohestani PhD), Saveh University of Medical Sciences, Saveh, Iran; School of Medicine (S Bagherieh BSc), Department of Environmental Health Engineering (A Fatehizadeh PhD), Isfahan University of Medical Sciences, Isfahan, Iran; Department of Social and Clinical Pharmacy (A S Bhagavathula PharmD), Charles University, Hradec Kralova, Czech Republic; Institute of Public Health (A S Bhagavathula PharmD), United Arab Emirates University, Al Ain, United Arab Emirates; Social Determinants of Health Research Center (A Bijani PhD), Babol University of Medical Sciences, Babol, Iran; Department of Family and Community Medicine (N S Butt PhD), Rabigh Faculty of Medicine (A A Malik PhD), Department of Dental Public Health (Z S Natto DrPH), King Abdulaziz University, Jeddah, Saudi Arabia; Environmental Health Unit (R Darvishi Cheshmeh Soltani PhD), Department of Nursing (M Golitaleb PhD), Department of Epidemiology (R Moradzadeh PhD, M Zamanian PhD), Arak University of Medical Sciences, Arak, Iran; Toxoplasmosis Research Center (Prof A Daryani PhD), Department of Immunology (Prof A Rafiei PhD), Molecular and Cell Biology Research Center (Prof A Rafiei PhD), Mazandaran University of Medical Sciences, Sari, Iran; Department of Epidemiology (M Dianatinasab MSc), Maastricht University, Maastricht, Netherlands; Department of Epidemiology (M Dianatinasab MSc), Non-communicable Disease Research Center (Prof R Malekzadeh MD, S G Sepanlou MD), Health Policy Research Center (S Shahabi PhD), Shiraz University of Medical Sciences, Shiraz, Iran; Biomedical Informatics and Medical Statistics Department (I El Sayed PhD), Alexandria University, Alexandria, Egypt; Faculty of Medicine (M Elhadi MD), University of Tripoli, Tripoli, Libya; Medicinal Chemistry (M Fereidoonnezhad PhD), Ahvaz Jundishapur University of Medical Sciences, Ahvaz, Iran; Department of Medical Parasitology (M Foroutan PhD), Faculty of Medicine (M Foroutan PhD), Abadan University of Medical Sciences, Abadan, Iran; Department of Genetics (P Goleij MSc), Sana Institute of Higher Education, Sari, Iran; Department of Family and Community Medicine (M I M Gubari PhD), University Of Sulaimani, Sulaimani, Iraq; Department of Radiology and Radiological Science (N Hafezi-Nejad MD, S Sheikhbahaei MD), Johns Hopkins University, Baltimore, MD, USA; School of Health and Environmental Studies (Prof S Hamidi DrPH), Hamdan Bin Mohammed Smart University, Dubai, United Arab Emirates; Research & Scientific Studies Unit (S Haque PhD), Jazan University, Jazan, Saudi Arabia; Institute of Pharmaceutical Sciences (K Hayat MS), University of Veterinary and Animal Sciences, Lahore, Pakistan; Department of Pharmacy Administration and Clinical Pharmacy (K Hayat MS), Xian Jiaotong University, Xian, China; College of Science and Engineering (Prof M Househ PhD), Hamad Bin Khalifa University, Doha, Qatar; Functional Neurosurgery Research Center (E Jamshidi PharmD), Shahid Beheshti University of Medical Sciences, Tehram, Iran; Division of Pulmonary Medicine (E Jamshidi PharmD), University of Lausanne (UNIL), Lausanne, Switzerland; Department of Parasitology (A Javadi Mamaghani PhD), Shahid Beheshti University of Medical Sciences, tehran, Iran; Gastrointestinal and Liver Diseases Research Center (F Joukar PhD, Prof F Mansour-Ghanaei MD, M Naghipour PhD), Caspian Digestive Disease Research Center (F Joukar PhD, Prof F Mansour-Ghanaei MD), Caspian Digestive Diseases Research Center (M Naghipour PhD), Guilan University of Medical Sciences, Rasht, Iran; Institute for Prevention of Non-communicable Diseases (R Kalhor PhD), Health Services Management Department (R Kalhor PhD), Qazvin University of Medical Sciences, Qazvin, Iran; Department of Public Health (Prof Y S Khader PhD), Jordan University of Science and Technology, Irbid, Jordan; Department of Medical Informatics (B Kiani PhD), Applied Biomedical Research Center (A Sahebkar PhD), Biotechnology Research Center (A Sahebkar PhD), Mashhad University of Medical Sciences, Mashhad, Iran; Radiology Department (S Livani MD), Infectious Diseases Research Center (S Mohammadi PhD), Golestan University of Medical Sciences, Gorgan, Iran; Department of Biostatistics and Epidemiology, School of public health (F Madadizadeh PhD), Yazd University of medical sciences, Yazd, Iran; Cancer Genetics Department (Prof K Majidzadeh-A PhD), Motamed Cancer Institute, Breast Cancer Research Center, Tehran, Iran; University Institute of Public Health (A A Malik PhD), The University of Lahore, Lahore, Pakistan; Internal Medicine Department (Y Mohammad MD), King Saud University, Riyadh, Saudi Arabia; Computer, Electrical, and Mathematical Sciences and Engineering Division (P Moraga PhD), King Abdullah University of Science and Technology, Thuwal, Saudi Arabia; Oral health policy and epidemiology (Z S Natto DrPH), Harvard University, Boston, USA; Health Promotion Research Center (H Okati-Aliabad PhD), Zahedan University of Medical Sciences, Zahedan, Iran; Department of Epidemiology (R Pakzad PhD), Ilam University of Medical Sciences, Ilam, Iran; Department of Public Health (Z Z Piracha PhD), Health Services Academy, Islamabad, Pakistan; Department of Pharmacology (F H Pottoo PhD), Imam Abdulrahman Bin Faisal University, Dammam, Saudi Arabia; Department of Community Medicine (V Rahmanian PhD), Jahrom University of Medical Sciences, Jahrom, Iran; Department of Clinical Science (M Rashidi DVM), Islamic Azad University, Garmsar, Iran; Department of Immunology and Laboratory Sciences (M Razeghinia MSc), Department of Medical Laboratory Sciences (M Sahebazzamani MSc), Sirjan School of Medical Sciences, Sirjan, Iran; Department of Immunology (M Razeghinia MSc), Kerman University of Medical Sciences, Kerman, Iran; Department of Epidemiology and Biostatistics (Prof M Rezaeian PhD), Department of Medical Biochemistry (M Sahebazzamani MSc), Rafsanjan University of Medical Sciences, Rafsanjan, Iran; Research and Development (Prof U Saeed PhD), Islamabad Diagnostic Center Pakistan, Islamabad, Pakistan; Biological Production Division (Prof U Saeed PhD), National Institute of Health, Islamabad, Pakistan; Department of Entomology (A M Samy PhD), Ain Shams University, Cairo, Egypt; Research Development Coordination Section (M N Saqib PhD), Pakistan Health Research Council, Islamabad, Pakistan; Faculty of Health & Social Sciences (B Sathian PhD), Bournemouth University, Bournemouth, Hampshire, UK; Independent Consultant, Karachi, Pakistan (M A Shaikh MD); Center for Biotechnology and Microbiology (M Suleman PhD), University of Swat, Mingora, Pakistan; School of Life Sciences (M Suleman PhD), Xiamen University, Xiamen, China; Department of Allied Health Sciences (I Ullah PhD), Iqra National University, Peshawar, Pakistan; Pakistan Council for Science and Technology (I Ullah PhD), Ministry of Science and Technology, Islamabad, Pakistan; Clinical Cancer Research Center (S Valadan Tahbaz PhD, S Yahyazadeh Jabbari MD), Milad General Hospital, Tehran, Iran; Department of Microbiology (S Valadan Tahbaz PhD), Faculty of Medicine (M Zahir MD), Islamic Azad University, Tehran, Iran; Cancer Institute (D Yuce MD), Hacettepe University, Ankara, Turkey; Research and Development Department (I Zare BSc), Sina Medical Biochemistry Technologies, Shiraz, Iran.

## Author contributions

All authors listed have made a substantial, direct, and intellectual contribution to the work and approved it for publication (**Supplementary Datasheet 2**). Author's are credited with: Providing data or critical feedback on data sources, Developing methods or computational machinery, Providing critical feedback on methods or results, Drafting the work or revising is critically for important intellectual content, and Managing the overall research enterprise.

## Funding

The GBD study is funded by Bill & Melinda Gates Foundation.

## Acknowledgments

S Aljundi acknowledges the Department of Health Policy and Management, Faculty of Public Health, Kuwait University and International Centre for Casemix and Clinical Coding, Faculty of Medicine, National University of Malaysia for their support. A M Samy acknowledges the support from the Egyptian Fulbright Mission Program and being a member of the Egyptian Young Academy of Sciences and Technology. S Haque is acknowledges support from the Deanship of Scientific Research, Jazan University, Saudi Arabia for providing the access of Saudi Digital Library for this research study.

## Conflict of interest

The authors declare that the research was conducted in the absence of any commercial or financial relationships that could be construed as a potential conflict of interest.

## Publisher’s note

All claims expressed in this article are solely those of the authors and do not necessarily represent those of their affiliated organizations, or those of the publisher, the editors and the reviewers. Any product that may be evaluated in this article, or claim that may be made by its manufacturer, is not guaranteed or endorsed by the publisher.

## Supplementary material

Supplementary Table 1 Data sources of GBD study 2019 for prostate cancer in North Africa and Middle East.

Supplementary Table 2 All-ages incidence, prevalence and death due to prostate cancer in 1990 and 2019 and their percent changes.

Supplementary Table 3 Incidence trends of prostate cancer between 1990 and 2019 among countries in North Africa and Middle East.

Supplementary Table 4 All-ages YLLs, YLDs, and DALYs due to prostate cancer in 1990 and 2019 and their percent changes.

Supplementary Table 5 All-ages YLLs, YLDs, and DALYs of prostate cancer attributable to smoking in 1990 and 2019 and their percent changes.

Supplementary Figure 1 Time trends of age-standardized and all-ages rates of incidence, prevalence, deaths, and DALYs due to prostate cancer among countries in North Africa and Middle East in 1990 and 2019.

Supplementary Figure 2 Time trends of age-standardized and all-ages rates of incidence, prevalence, deaths, and DALYs due to prostate cancer in North Africa and Middle East in 1990 and 2019.

Supplementary Figure 3 Rates of incidence, prevalence, deaths, YLLs, YLDs, and DALYs due to prostate cancer among age groups in countries in North Africa and Middle East in 1990 and 2019.

Supplementary Figure 4 Age-standardized rates of incidence **(A)**, prevalence **(B)**, deaths **(C)**, and DALYs **(D)** due to prostate cancer among countries in North Africa and Middle East disaggregated by HAQI.
