# Supplementary material for: The burden of prostate cancer in North Africa and Middle East, 1990–2019: Findings from the global burden of disease study
Source: Front Oncol. 2022 Sep 13;12:961086. doi: 10.3389/fonc.2022.961086 (PMC9513750; doi:10.3389/fonc.2022.961086)

Supplementary Table 1

Title	Time period covered	Coverage type	Geography	Data type	Secondary data type	Provider	Dataset URL
Algeria - Algiers Cancer Registry Report 2003	01/2003 to 12/2003	Subnational	Algeria, Alger	Disease registry		National Institute of Public Health (Algeria)	http://www.ands.dz/insp/registre-tumeurs.htm
Algeria - Algiers Cancer Registry Report 2004	01/2004 to 12/2004	Subnational	Algeria, Alger	Disease registry		National Institute of Public Health (Algeria)	http://www.ands.dz/insp/registre-tumeurs.htm
Algeria - Algiers Cancer Registry Report 2006	01/2006 to 12/2006	Subnational	Algeria, Alger	Disease registry		National Institute of Public Health (Algeria)	http://www.ands.dz/insp/registre-tumeurs.htm
Algeria - Algiers Cancer Registry Report 2007	01/2007 to 12/2007	Subnational	Algeria, Alger	Disease registry		National Institute of Public Health (Algeria)	http://www.ands.dz/insp/registre-tumeurs.htm
Algeria - Batna Cancer Registy Report 2000-2006	01/2000 to 12/2006	Subnational	Algeria, Batna	Disease registry		University Hospital of Batna	http://www.sante.dz/chu-batna/
Algeria - Oran Cancer Registry Report 2005	01/2005 to 12/2005	Subnational	Algeria, Oran	Disease registry		Oran Cancer Registry (Algeria)	
Algeria - Oran Cancer Registry Report 2006	01/2006 to 12/2006	Subnational	Algeria, Oran	Disease registry		Oran Cancer Registry (Algeria)	
Algeria - Setif Cancer Registry 1986-1989 - CI5	01/1986 to 12/1989		Algeria, SÃ©tif	Disease registry			
Algeria - Setif Cancer Registry 1990-1993 - CI5	01/1990 to 12/1993		Algeria, SÃ©tif	Disease registry			
Algeria - Setif Cancer Registry 1998-2002 - CI5	01/1998 to 12/2002		Algeria, SÃ©tif	Disease registry			
Algeria - Setif Cancer Registry 2003-2007 - CI5	01/2003 to 12/2007		Algeria, SÃ©tif	Disease registry			
Algeria - Study of Causes of Death, TAHINA 2002	01/2002 to 12/2002	Country	Algeria	Report	Verbal autopsy	National Institute of Public Health (Algeria)	http://www.ands.dz/insp/tahina.html

Title	Time period covered	Coverage type	Geography	Data type	Secondary data type	Provider	Dataset URL
Algeria Tumour Registry of Algiers 1993-1997 - CI5	01/1993 to 12/1997		Algeria, Alger	Disease registry			
Bahrain Cancer Registry 1998-2002 - CI5	01/1998 to 12/2002		Bahrain	Disease registry			
Bahrain Cancer Registry 2003-2007 - CI5	01/2003 to 12/2007		Bahrain	Disease registry			
Cancer in Kuwait: magnitude of the problem	01/1974 to 12/2007		Bahrain, Kuwait, Oman, Qatar, Saudi Arabia, United Arab Emirates	Scientific literature			
Cancer incidence in Jordan, 1996-2005	01/1996 to 12/2005		Jordan	Scientific literature			
Cancer Incidence in Oman 2013	01/2013 to 12/2013	Country	Oman	Disease registry		Ministry of Health (Oman)	https://internal.moh.gov.om/en/web/general-directorate-of-primary-health-care/resources?p_p_id=2_WAR_kaleodesignerportlet&p_p_lifecycle=0
Cancer Incidence in Postwar Lebanon: Findings from the First National Population-based Registry, 1998	01/1998 to 12/1998		Lebanon	Scientific literature			
Cancer Occurrence in Ardabil: Results of a Population-Based Cancer Registry from Iran	01/1996 to 12/1999	Subnational	Iran (Islamic Republic of), ArdabÄ«l	Scientific literature			
Declining incidence of esophageal cancer in the Turkmen Plain, eastern part of the Caspian Littoral of Iran: A retrospective cancer surveillance	01/1996 to 12/2000	Subnational	Iran (Islamic Republic of), GolestÄ�	Scientific literature			
Egypt - Aswan National Cancer Registry Report 2008	01/2008 to 12/2008	Subnational	Egypt, AswÄ�	Disease registry		National Cancer Registry Program of Egypt	http://www.cancerregistry.gov.eg/Default.aspx
Egypt - Damietta National Cancer Registry Report 2009	01/2009 to 12/2009	Subnational	Egypt, DumyÄ�Å£	Disease registry		National Cancer Registry Program of Egypt	http://www.cancerregistry.gov.eg/Default.aspx

Title	Time period covered	Coverage type	Geography	Data type	Secondary data type	Provider	Dataset URL
Egypt - Damietta National Cancer Registry Statistics 2010	01/2010 to 12/2010	Subnational	Egypt, DumyÄ�Å£	Disease registry		National Cancer Registry Program of Egypt	http://www.cancerregistry.gov.eg/
Egypt - El-Minia National Cancer Registry Report 2009	01/2009 to 12/2009	Subnational	Egypt, Al MinyÄ�	Disease registry		National Cancer Registry Program of Egypt	http://www.cancerregistry.gov.eg/Default.aspx
Egypt - Gharbiah Cancer Registry 1999-2002 - CI5	01/1999 to 12/2002		Egypt, Al GharbÄ«yah	Disease registry			
Egypt - Gharbiah Cancer Registry 2003-2007 - CI5	01/2003 to 12/2007		Egypt, Al GharbÄ«yah	Disease registry			
Iran - Ardabil Cancer Registry Extracts 1985-2008	01/1985 to 12/2008	Subnational	Iran (Islamic Republic of), ArdabÄ«l	Disease registry			
Iran - Golestan Cancer Registry 2005-2007 - CI5	01/2005 to 12/2007	Subnational	Iran (Islamic Republic of), GolestÄ�	Disease registry			
Iran - Golestan Cancer Registry Incidence Data 2006-2008	01/2006 to 12/2008	Subnational	Iran (Islamic Republic of), GolestÄ�	Disease registry			
Iran National Cancer Registry 2000	01/2000 to 12/2000	Country	Iran (Islamic Republic of)	Disease registry	Subnationally representative		
Iran National Cancer Registry 2001	01/2001 to 12/2001	Country	Iran (Islamic Republic of)	Disease registry	Subnationally representative		
Iran National Cancer Registry 2002	01/2002 to 12/2002	Country	Iran (Islamic Republic of)	Disease registry	Subnationally representative		
Iran National Cancer Registry 2003	01/2003 to 12/2003	Country	Iran (Islamic Republic of)	Disease registry	Subnationally representative		

Title	Time period covered	Coverage type	Geography	Data type	Secondary data type	Provider	Dataset URL
Iran National Cancer Registry 2004	01/2004 to 12/2004	Country	Iran (Islamic Republic of)	Disease registry	Subnationally representative		
Iran National Cancer Registry 2005	01/2005 to 12/2005	Country	Iran (Islamic Republic of)	Disease registry	Subnationally representative		
Iran National Cancer Registry 2006	01/2006 to 12/2006	Country	Iran (Islamic Republic of)	Disease registry	Subnationally representative		
Iran National Cancer Registry 2007	01/2007 to 12/2007	Country	Iran (Islamic Republic of)	Disease registry	Subnationally representative		
Iran National Cancer Registry 2008	01/2008 to 12/2008	Country	Iran (Islamic Republic of)	Disease registry	Subnationally representative		
Iran National Cancer Registry 2009	01/2009 to 12/2009	Country	Iran (Islamic Republic of)	Disease registry	Subnationally representative		
Iran National Cancer Registry 2010	01/2010 to 12/2010	Country	Iran (Islamic Republic of)	Disease registry	Subnationally representative		
Iran National Cancer Registry Report 2003-2004	03/2003 to 02/2004	Country	Iran (Islamic Republic of)	Disease registry			
Iran National Cancer Registry Report 2004-2005	03/2004 to 02/2005	Country	Iran (Islamic Republic of)	Disease registry			
Iran National Cancer Registry Report 2005-2006	03/2005 to 02/2006	Country	Iran (Islamic Republic of)	Disease registry			
Iran National Cancer Registry Report 2006-2007	03/2006 to 02/2007	Country	Iran (Islamic Republic of)	Disease registry			
Iran National Cancer Registry Report 2009-2010	03/2009 to 03/2010	Country	Iran (Islamic Republic of)	Disease registry			

Title	Time period covered	Coverage type	Geography	Data type	Secondary data type	Provider	Dataset URL
Iraq Cancer Registry 2011	01/2011 to 12/2011	Country	Iraq	Disease registry			
Jordan Cancer Incidence Report 2001	01/2001 to 12/2001	Country	Jordan	Disease registry		Ministry of Health (Jordan)	http://www.moh.gov.jo/EN/Pages/Periodic-Newsletters.aspx
Jordan Cancer Incidence Report 2002	01/2002 to 12/2002	Country	Jordan	Disease registry		Ministry of Health (Jordan)	http://www.moh.gov.jo/EN/Pages/Periodic-Newsletters.aspx
Jordan Cancer Incidence Report 2003	01/2003 to 12/2003	Country	Jordan	Disease registry		Ministry of Health (Jordan)	http://www.moh.gov.jo/EN/Pages/Periodic-Newsletters.aspx
Jordan Cancer Incidence Report 2004	01/2004 to 12/2004	Country	Jordan	Disease registry		Ministry of Health (Jordan)	http://www.moh.gov.jo/EN/Pages/Periodic-Newsletters.aspx
Jordan Cancer Incidence Report 2005	01/2005 to 12/2005	Country	Jordan	Disease registry		Ministry of Health (Jordan)	http://www.moh.gov.jo/EN/Pages/Periodic-Newsletters.aspx
Jordan Cancer Incidence Report 2006	01/2005 to 12/2006	Country	Jordan	Disease registry		Ministry of Health (Jordan)	http://www.moh.gov.jo/EN/Pages/Periodic-Newsletters.aspx
Jordan Cancer Incidence Report 2007	01/2006 to 12/2007	Country	Jordan	Disease registry		Ministry of Health (Jordan)	http://www.moh.gov.jo/EN/Pages/Periodic-Newsletters.aspx
Jordan Cancer Incidence Report 2008	01/2007 to 12/2008	Country	Jordan	Disease registry		Ministry of Health (Jordan)	http://www.moh.gov.jo/EN/Pages/Periodic-Newsletters.aspx
Jordan Cancer Incidence Report 2009	01/2008 to 12/2009	Country	Jordan	Disease registry		Ministry of Health (Jordan)	http://www.moh.gov.jo/EN/Pages/Periodic-Newsletters.aspx
Jordan Cancer Incidence Report 2011	01/2011 to 12/2011	Country	Jordan	Disease registry		Ministry of Health (Jordan)	http://www.moh.gov.jo/Pages/viewpage.aspx?pageID=248
Jordan Cancer Incidence Report 2012	01/2012 to 12/2012	Country	Jordan	Disease registry		Ministry of Health (Jordan)	http://www.moh.gov.jo/Pages/viewpage.aspx?pageID=248
Kuwait Cancer Registry 1979-1982 - CI5	01/1979 to 12/1982		Kuwait	Disease registry			

Title	Time period covered	Coverage type	Geography	Data type	Secondary data type	Provider	Dataset URL
Kuwait Cancer Registry 1983-1987 - CI5	01/1983 to 12/1987		Kuwait	Disease registry			
Kuwait Cancer Registry 2003-2007 - CI5	01/2003 to 12/2007		Kuwait	Disease registry			
Lebanon National Cancer Registry Tables 2005	01/2005 to 12/2005	Country	Lebanon	Disease registry		Ministry of Public Health (Lebanon)	http://www.moph.gov.lb/Prevention/Surveillance/Pages/Cancer.aspx
Lebanon National Cancer Registry Tables 2006	01/2006 to 12/2006	Country	Lebanon	Disease registry		Ministry of Public Health (Lebanon)	http://www.moph.gov.lb/Prevention/Surveillance/Pages/Cancer.aspx
Lebanon National Cancer Registry Tables 2007	01/2007 to 12/2007	Country	Lebanon	Disease registry		Ministry of Public Health (Lebanon)	http://www.moph.gov.lb/Prevention/Surveillance/Pages/Cancer.aspx
Lebanon Statistical Bulletin 2013	01/2007 to 12/2013	Country	Lebanon	Report	Epi surveillance, Financial, Subnationally representative	Ministry of Public Health (Lebanon)	https://www.moph.gov.lb/ar/publications
Libya - Benghazi Cancer Registry 2003-2005 - CI5	01/2003 to 12/2005		Libya, BanghÄzÄ«	Disease registry			
Morocco - Cancer Registry of Greater Casablanca 2004	01/2004 to 12/2004	Subnational	Morocco, Grand Casablanca	Disease registry	Subnationally representative, Urban-rural representative	Lalla Salma Association to Fight Against Cancer (Morocco)	http://www.contrelecancer.ma/en/
Morocco - Cancer Registry of Greater Casablanca Region 2005 2006 2007	01/2005 to 12/2007	Subnational	Morocco, Grand Casablanca	Disease registry	Subnationally representative	Lalla Salma Association to Fight Against Cancer (Morocco)	http://www.contrelecancer.ma/en/documents/registre-des-cancers-de-la-region-du-grand-casab-2/

Title	Time period covered	Coverage type	Geography	Data type	Secondary data type	Provider	Dataset URL
Oman - Cancer Incidence in Oman 2001	01/2001 to 12/2001	Country	Oman	Disease registry		Ministry of Health (Oman)	http://www.moh.gov.om/en/nv_menu.php?o=reports/report.htm
Oman - Cancer Incidence in Oman 2002	01/2002 to 12/2002	Country	Oman	Disease registry		Ministry of Health (Oman)	http://www.moh.gov.om/en/nv_menu.php?o=reports/report.htm
Oman - Cancer Incidence in Oman 2003	01/2003 to 12/2003	Country	Oman	Disease registry		Ministry of Health (Oman)	http://www.moh.gov.om/en/nv_menu.php?o=reports/report.htm
Oman - Cancer Incidence in Oman 2004	01/2004 to 12/2004	Country	Oman	Disease registry		Ministry of Health (Oman)	http://www.moh.gov.om/en/nv_menu.php?o=reports/report.htm
Oman - Cancer Incidence in Oman 2005	01/1996 to 12/2005	Country	Oman	Disease registry		Ministry of Health (Oman)	http://www.moh.gov.om/en/nv_menu.php?o=reports/report.htm
Oman - Cancer Incidence in Oman 2006	01/1996 to 12/2006	Country	Oman	Disease registry		Ministry of Health (Oman)	http://www.moh.gov.om/en/nv_menu.php?o=reports/report.htm
Oman - Cancer Incidence in Oman 2007	01/1997 to 12/2007	Country	Oman	Disease registry		Ministry of Health (Oman)	http://www.moh.gov.om/en/nv_menu.php?o=reports/report.htm
Oman - Cancer Incidence in Oman 2008	01/2008 to 12/2008	Country	Oman	Disease registry		Ministry of Health (Oman)	http://www.moh.gov.om/en/nv_menu.php?o=reports/report.htm
Oman - Cancer Incidence in Oman 2011	01/2011 to 12/2011	Country	Oman	Disease registry		Ministry of Health (Oman)	http://www.moh.gov.om/en/nv_menu.php?o=reports/report.htm

Title	Time period covered	Coverage type	Geography	Data type	Secondary data type	Provider	Dataset URL
Oman - Cancer Incidence in Oman 2012	01/2012 to 12/2012	Country	Oman	Disease registry		Ministry of Health (Oman)	https://www.moh.gov.om/en/web/statistics/annual-reports
Oman - Cancer Incidence in Oman Report of 2009	01/1997 to 12/2009	Country	Oman	Disease registry		Ministry of Health (Oman)	http://www.moh.gov.om/en/nv_menu.php?o=reports/report.htm
Oman - Cancer Incidence in Oman Report of 2010	01/1998 to 12/2010	Country	Oman	Disease registry		Ministry of Health (Oman)	http://www.moh.gov.om/en/nv_menu.php?o=reports/report.htm
Oman Cancer Registry 1993-1997 - CI5	01/1993 to 12/1997		Oman	Disease registry			
Oman Cancer Registry 1998-2001 - CI5	01/1998 to 12/2001		Oman	Disease registry			
Palestine Health Status Annual Report 1999	01/1996 to 12/1999	Country	Palestine	Report		Ministry of Health (Palestine)	http://www.moh.ps/
Palestine Health Status Annual Report 2000	01/2000 to 12/2000	Country	Palestine	Report		Ministry of Health (Palestine)	http://www.moh.ps/
Palestine Health Status Annual Report 2001	01/1997 to 12/2001	Country	Palestine	Report	Epi surveillance, Subnationally representative	Ministry of Health (Palestine)	http://www.moh.ps/
Palestine Health Status Annual Report 2010	01/2010 to 12/2010	Country	Palestine	Report		Ministry of Health (Palestine)	https://www.site.moh.ps/index/Books/BookType/2/Language/ar
Palestine Health Status Annual Report 2011	01/2002 to 12/2011	Country	Palestine	Report	Epi surveillance	Ministry of Health (Palestine)	https://www.site.moh.ps/index/Books/BookType/2/Language/ar

Title	Time period covered	Coverage type	Geography	Data type	Secondary data type	Provider	Dataset URL
Palestine Health Status Annual Report 2012	01/2002 to 12/2012	Country	Palestine	Report	Epi surveillance, Subnationally representative	Ministry of Health (Palestine)	https://www.site.moh.ps/index/Books/BookType/2/Language/ar
Palestine Health Status Annual Report 2013	01/2007 to 12/2013	Country	Palestine	Report	Epi surveillance, Subnationally representative	Ministry of Health (Palestine)	https://www.site.moh.ps/index/Books/BookType/2/Language/ar
Palestine Health Status Annual Report 2014	01/2008 to 12/2014	Country	Palestine	Report	Epi surveillance, Subnationally representative	Ministry of Health (Palestine)	https://www.site.moh.ps/index/Books/BookType/2/Language/ar
Palestine Health Status Annual Report 2015	01/2009 to 12/2015	Country	Palestine	Report	Epi surveillance, Subnationally representative	Ministry of Health (Palestine)	https://www.site.moh.ps/index/Books/BookType/2/Language/ar
Palestine Health Status Annual Report 2016	01/2009 to 12/2016	Country	Palestine	Report	Epi surveillance, Subnationally representative	Ministry of Health (Palestine)	https://www.site.moh.ps/index/Books/BookType/2/Language/ar
Palestine Health Status Annual Report 2017	01/1988 to 12/2017	Country	Palestine	Report	Epi surveillance, Subnationally representative	Ministry of Health (Palestine)	https://www.site.moh.ps/index/Books/BookType/2/Language/ar
The Epidemiology and Trend of Cancer in Jordan, 2000-2013	01/2000 to 12/2013		Jordan	Scientific literature			

Supplementary Table 2

Location		Incidence			Prevalence			Deaths		
		**1990 (95% UI)**	**2019 (95% UI)**	**Percent changes (95% UI)**	**1990 (95% UI)**	**2019 (95% UI)**	**Percent changes (95% UI)**	**1990 (95% UI)**	**2019 (95% UI)**	**Percent changes (95% UI)**
**North Africa and Middle East**		9552.1 (7684.4-11523.6)	47473.6 (36988.5-55846.8)	397 (322.1-501.4)	55690.7 (45332.9-65113.6)	348924 (271316.9-407425.5)	526.5 (433.9-647.4)	6647.8 (5380.2-8230.6)	19088.9 (15241.1-22498.5)	187.1 (147.6-246.7)
**Countries**	Afghanistan	359.5 (268.4-490.2)	585.3 (438.5-744.4)	62.8 (22.5-112)	1312.9 (1027.3-1692.8)	2648.8 (1959.5-3351.8)	101.7 (48.2-168.5)	351.4 (256.2-488.3)	505.2 (377.5-661.8)	43.8 (9.5-83.3)
	Algeria	680.9 (497.3-888.2)	2844.7 (2104.5-3955.3)	317.8 (200.2-464.6)	4108.7 (3079.2-5297.1)	20966.6 (15497.5-28937.4)	410.3 (258.1-596.4)	468 (349.2-608.1)	1279.9 (960.1-1739.3)	173.5 (102.1-258.4)
	Bahrain	15.4 (11.6-19.3)	165.8 (116.4-236.1)	974.8 (607.6-1524.6)	101.9 (78.6-128)	1411.1 (986.5-2006.1)	1285.5 (793-1965.7)	8.6 (6.4-10.6)	36.3 (27.2-47.7)	323.5 (201.6-490.7)
	Egypt	856.3 (726.5-1062.7)	3800.4 (2623.9-5430.7)	343.8 (215.7-542.6)	4650.1 (3887.8-5503.7)	25772.3 (17595.5-37084.1)	454.2 (291.1-687.5)	658.8 (559.9-853.6)	1807.7 (1293-2508.6)	174.4 (99.6-283.8)
	Iran (Islamic Republic of)	1778.7 (1352.6-2186.8)	10423.6 (7299.2-12189.4)	486 (365.5-653.2)	12637.8 (9501.6-15417.1)	79026.6 (55832.2-93309.9)	525.3 (393.1-701.4)	917.4 (725.3-1150.3)	3916.3 (2912.3-4438.7)	326.9 (249.1-435.7)
	Iraq	357.3 (249.2-496.6)	1917.4 (1425.7-2465.9)	436.6 (277.5-676.9)	1919.2 (1406.7-2561.9)	14028.3 (10357.6-17976.3)	630.9 (417.6-949.3)	267.5 (189.9-381.4)	861.4 (651.3-1158)	222 (136.3-343.6)
	Jordan	73.7 (57.2-95.4)	738.9 (520.7-996.6)	902.4 (622.2-1283.5)	471.8 (367.5-610.1)	5740.5 (4105-7754.8)	1116.7 (779.3-1610.7)	46.4 (36.2-58.7)	256.5 (179.9-334.4)	452.4 (314.7-639.2)
	Kuwait	53.2 (40.4-67.7)	428.9 (313.1-596.8)	705.8 (505.3-951.1)	445.3 (343.8-558.7)	3572.2 (2623.2-4969.4)	702.1 (498.5-953.8)	16.2 (12.3-20.7)	105.8 (78-138.6)	551.7 (422.6-703.6)
	Lebanon	294.3 (225.5-373.9)	1722.5 (1112.2-2422.6)	485.3 (306.9-727.7)	1764 (1371.5-2228.2)	12734.4 (8470.7-17699.8)	621.9 (396.3-926.8)	182.5 (135.8-229.7)	587.9 (363.6-858)	222.2 (124.2-353.7)
	Libya	111 (70.3-150.7)	453 (328-605.6)	308.3 (174.6-548.2)	694.4 (465.4-914.6)	3205.5 (2318.3-4283.7)	361.6 (211-617.5)	72.9 (47.1-99.5)	207.9 (156.1-271.4)	185.1 (101.9-338.3)
	Morocco	484 (361-608.1)	2134 (1517.4-2792.8)	340.9 (214.9-478.9)	2522.9 (1946.5-3128.2)	14093.5 (10035.9-18512.3)	458.6 (301.1-635.8)	388.6 (283.7-505.8)	1184.8 (871.1-1563.8)	204.9 (127.3-292.8)
	Oman	41.8 (27.3-58.7)	246.2 (182.1-342.5)	488.4 (311.3-731.6)	303.6 (207.5-419.8)	2085 (1514.9-2894.6)	586.8 (382.7-870.4)	20.6 (13.6-29)	54.1 (44.2-70)	162.6 (87.9-301.5)
	Palestine	81.6 (56-113.4)	294.4 (198.6-377.9)	260.7 (150.6-447.2)	455.9 (323.7-627.9)	2171.7 (1527.6-2764.2)	376.4 (234.3-609.2)	57.7 (39.3-79.9)	122.5 (80.2-155.3)	112.3 (50.4-227.1)
	Qatar	8.4 (6.1-12.1)	224.1 (140.7-362.9)	2575.3 (1586.1-4001)	65.7 (48.9-94.5)	1989.5 (1258.6-3207.9)	2926 (1834.9-4448.2)	3.5 (2.7-4.8)	31.7 (21.9-45.3)	797.1 (522.9-1214.3)
	Saudi Arabia	341.4 (203.9-532.3)	2303.2 (1655.5-3462.7)	574.7 (328.9-1303.6)	2239.1 (1468.1-3312.2)	19565.3 (14181.7-29201)	773.8 (464.9-1574.1)	225.9 (135-357)	480.4 (363.8-719.4)	112.7 (45.1-336.8)
	Sudan	389 (274.2-535.7)	1291.3 (892.6-1748)	232 (145.2-361.9)	1777.4 (1328.3-2379.8)	7514 (5109.8-10230.2)	322.8 (210.6-482.9)	343.6 (241.7-479)	866.8 (610.2-1168.1)	152.2 (94.8-242.5)
	Syrian Arab Republic	284.7 (194.8-433.5)	1168.2 (799.9-1858.3)	310.3 (167-553.6)	1733.3 (1290.1-2397.7)	8738.6 (6093.4-13697)	404.2 (228-683.6)	194.8 (125.2-320.5)	451.6 (307.6-732.8)	131.8 (58.4-269.1)
	Tunisia	266.8 (196-352.7)	1266.3 (878.1-1796.9)	374.7 (213.2-622.5)	1714.1 (1291.9-2221.2)	9575.2 (6765.7-13545.7)	458.6 (278.2-745.2)	168.5 (123.9-221.4)	462.7 (330.2-648.3)	174.7 (87.5-312.7)
	Turkey	2865.7 (2004-3660.6)	14001.8 (9792.1-18451.5)	388.6 (248.7-572.7)	15603.4 (11104.3-20078.7)	103771.1 (72808.4-138053.8)	565.1 (375.8-830.9)	2101.7 (1491.4-2662.5)	5185.3 (3701.8-6591.5)	146.7 (82.3-228.4)
	United Arab Emirates	32.4 (19.8-54.9)	624.4 (323.5-1195.9)	1824.7 (1093.7-2813.1)	249.6 (159.9-407.6)	5415.5 (2897.2-10121.5)	2069.8 (1263.3-3193.1)	14.6 (9-24.6)	126.7 (69.6-241)	768.6 (478.4-1160.8)
	Yemen	169.5 (111.4-243)	791.1 (567.6-1069.7)	366.6 (240.9-591.6)	882 (623-1237.1)	4543.7 (3280.3-6230.7)	415.2 (272.8-666.1)	134 (85.9-194.3)	538 (394.3-713.8)	301.4 (199-476.3)

Supplementary Table 3

Location		New cases		Expected new cases in 2019		1990 - 2019 new cases change cause			1990 - 2019 new cases overall change
		**1990**	**2019**	**Population growth**	**Population growth + Aging**	**% Population growth**	**% Age structure change**	**% Incidence rate change**	
**North Africa and Middle East**		9552	47474	17090	25100	78.9%	83.9%	234.2%	397%
**Countries**	Afghanistan	360	585	1250	486	247.7%	-212.6%	27.7%	62.8%
	Algeria	681	2845	1129	2059	65.8%	136.6%	115.4%	317.8%
	Bahrain	15	166	47	96	204.8%	320.7%	449.2%	974.8%
	Egypt	856	3800	1543	2090	80.2%	63.9%	199.7%	343.8%
	Iran (Islamic Republic of)	1779	10424	2545	5332	43.1%	156.7%	286.3%	486%
	Iraq	357	1917	859	987	140.3%	36%	260.4%	436.6%
	Jordan	74	739	232	390	214.6%	215.1%	472.7%	902.4%
	Kuwait	53	429	125	220	135.2%	178.9%	391.7%	705.8%
	Lebanon	294	1722	453	705	54%	85.7%	345.6%	485.3%
	Libya	111	453	174	289	57%	103.6%	147.6%	308.3%
	Morocco	484	2134	694	1119	43.4%	87.8%	209.7%	340.9%
	Oman	42	246	109	117	160.6%	18.5%	309.3%	488.4%
	Palestine	82	294	198	207	142.1%	11.8%	106.7%	260.7%
	Qatar	8	224	60	89	618.7%	337.8%	1618.8%	2575.3%
	Saudi Arabia	341	2303	794	963	132.5%	49.5%	392.7%	574.7%
	Sudan	389	1291	789	792	102.8%	0.8%	128.4%	232%
	Syrian Arab Republic	285	1168	305	667	7%	127.2%	176.1%	310.3%
	Tunisia	267	1266	359	653	34.7%	110.2%	229.8%	374.7%
	Turkey	2866	14002	3886	7542	35.6%	127.6%	225.4%	388.6%
	United Arab Emirates	32	624	180	322	454.1%	439.7%	930.9%	1824.7%
	Yemen	170	791	386	527	127.7%	83.2%	155.7%	366.6%

Supplementary Table 4

Location		YLLs[1]			YLDs[2]			DALYs[3]		
		**1990 (95% UI)**	**2019 (95% UI)**	**Percent changes (95% UI)**	**1990 (95% UI)**	**2019 (95% UI)**	**Percent changes (95% UI)**	**1990 (95% UI)**	**2019 (95% UI)**	**Percent changes (95% UI)**
**North Africa and Middle East**		120603.2 (98236.2-146994.6)	317372.1 (249856.6-372106.1)	163.2 (126.9-216.6)	5158.3 (3592.8-6999.2)	28390.3 (19167-38271.9)	450.4 (364.8-572)	125761.5 (102033.9-153135.7)	345762.4 (270783-404981.7)	174.9 (136.2-229.8)
**Countries**	Afghanistan	6441 (4826.3-8748.2)	9123.8 (6883.5-11558.3)	41.7 (7.4-81.9)	156.9 (101.1-230.5)	268 (172.4-382.2)	70.8 (23.1-138.8)	6597.9 (4945.1-8969.5)	9391.9 (7112.3-11931.7)	42.3 (8.2-82.8)
	Algeria	8380.4 (6111.5-10902.9)	20553.4 (15170.6-27904.7)	145.3 (78.1-228.9)	366.1 (232.4-528.4)	1650.5 (1075.2-2486.4)	350.8 (209.2-530.2)	8746.5 (6408.5-11387.9)	22203.9 (16364.3-30443.9)	153.9 (83.5-240.3)
	Bahrain	169.3 (129-206.2)	730.6 (534.3-1001)	331.6 (194.8-527.9)	9.2 (6.2-13.2)	103.2 (62.1-161.3)	1027.8 (615.1-1582.2)	178.4 (136.5-217.5)	833.8 (607.3-1152.1)	367.3 (220.1-574.2)
	Egypt	12299.8 (10428.5-15288.3)	33227.4 (23558.1-46517.7)	170.1 (94.5-284.7)	470.4 (327.5-641.8)	2367.3 (1432.8-3716)	403.2 (232.3-627.6)	12770.2 (10828.2-15910)	35594.7 (25029.7-49721.9)	178.7 (100.7-295.4)
	Iran (Islamic Republic of)	18240.4 (14374.6-22355)	61523.5 (45527.8-69323.1)	237.3 (179.3-316.6)	1050 (696.1-1503)	6204.1 (4043.2-8647.2)	490.9 (367.9-660.1)	19290.3 (15181.8-23620.3)	67727.5 (50159.7-76614.3)	251.1 (189.6-333.1)
	Iraq	4550.5 (3206.5-6482)	15057.8 (11256.5-19536.3)	230.9 (138.5-369.6)	186.3 (117.6-275.3)	1099.4 (687.3-1613.4)	490 (295.2-787.1)	4736.8 (3336.5-6744.6)	16157.2 (12158.9-20976.4)	241.1 (144.5-383.6)
	Jordan	839.5 (648.5-1070.6)	4402.9 (3079.9-5760.7)	424.4 (287.6-596.5)	41.3 (27.4-58.6)	448.9 (270-665.1)	988.1 (669.6-1447.7)	880.8 (683.6-1123.6)	4851.8 (3416-6323.5)	450.8 (305.9-637.1)
	Kuwait	329.9 (248.9-419.6)	1663.2 (1231.5-2202.3)	404.2 (294.4-524.6)	33.9 (22-48.7)	263.5 (166.8-396.8)	677.3 (458.4-959.9)	363.8 (275.4-456.5)	1926.8 (1415.3-2598.4)	429.7 (314.4-556.9)
	Lebanon	3114.9 (2367.3-3862.5)	8756.1 (5506.4-12457.6)	181.1 (94.5-292)	165.1 (111.5-240.5)	1049.3 (626.9-1605.1)	535.6 (331.1-805.6)	3280 (2503.2-4101.9)	9805.4 (6234.9-13905)	198.9 (107.3-315.9)
	Libya	1231.7 (794-1661.5)	3460 (2554.1-4519.1)	180.9 (97.2-331.7)	60.6 (35.5-90.3)	268.4 (172.6-396.6)	342.9 (192.3-612.7)	1292.3 (834.5-1742.7)	3728.5 (2736.4-4884.2)	188.5 (102.1-343.7)
	Morocco	6973.1 (5222.1-8765.3)	20244.9 (14661-26472.4)	190.3 (112.4-276.8)	243 (161.6-338.7)	1195.4 (742.2-1738.5)	391.9 (244-576.1)	7216.1 (5406.4-9071.8)	21440.3 (15509.8-27935.5)	197.1 (117.8-285.1)
	Oman	424.6 (279.8-599.3)	1069.1 (850.2-1422.7)	151.8 (81.6-262.8)	26 (15.2-39.4)	160.4 (101.6-245.6)	516.1 (330.3-769.7)	450.6 (295.7-631.3)	1229.5 (967.4-1649.4)	172.8 (97.3-289.5)
	Palestine	953.2 (652.6-1306.2)	2176.8 (1466.8-2752.3)	128.4 (60.7-247.8)	43.3 (26.5-68.3)	172.8 (108.4-247.1)	299.5 (176.7-519.5)	996.4 (679.2-1375.5)	2349.7 (1590.4-2972.7)	135.8 (65.6-258.7)
	Qatar	78.7 (59-110)	704.4 (468.8-1044.6)	794.8 (503.1-1219.5)	5.3 (3.4-8)	143.1 (79.3-248.8)	2596.6 (1570.5-4025.3)	84 (63-116.1)	847.5 (555.3-1277.1)	908.6 (570.5-1406.3)
	Saudi Arabia	4144.5 (2512.3-6487.3)	9885 (7442-14908.6)	138.5 (59.3-394.8)	188.5 (103.4-300.6)	1466.5 (939.3-2306.7)	678 (390-1510.1)	4333 (2609.7-6789.5)	11351.5 (8493-16850.3)	162 (73.5-441.2)
	Sudan	6251.7 (4420.5-8654.2)	14704.3 (10191.7-19926.2)	135.2 (77.6-222)	188.3 (118.6-285.9)	684 (417-1023.4)	263.2 (155.7-423.4)	6440 (4570.4-8889.6)	15388.3 (10624.7-20876.2)	138.9 (80.7-227.5)
	Syrian Arab Republic	3464.2 (2336.9-5354.7)	7991.3 (5446.6-12789.4)	130.7 (54.4-264.8)	157.2 (97.5-247.4)	717.8 (428.6-1187.7)	356.5 (189.9-642.1)	3621.4 (2443.3-5598.9)	8709 (5997.1-13816.2)	140.5 (62.3-276.8)
	Tunisia	2926.5 (2165.1-3853)	7449 (5306.8-10553.2)	154.5 (71.1-278.9)	149.4 (95.4-215.4)	773 (478.2-1182.1)	417.5 (245.9-693.9)	3075.8 (2284.1-4036.1)	8222 (5835.2-11754.5)	167.3 (80.4-297.4)
	Turkey	36637.7 (25971.3-46046.2)	80979.6 (57964.8-104215.8)	121 (64-194.9)	1504.4 (954.2-2234.5)	8488.4 (5292.5-12427.4)	464.2 (294.4-695.5)	38142.2 (26980.5-48165)	89468.1 (63878.9-116480.8)	134.6 (73.4-214.5)
	United Arab Emirates	357.3 (224.7-588.8)	3588.4 (1931.7-6690.5)	904.3 (553.1-1388.2)	20.4 (11.2-35.7)	412.4 (194.1-833.6)	1921 (1148-2963.3)	377.7 (237.6-621.3)	4000.9 (2152.7-7465.6)	959.2 (589.1-1458.1)
	Yemen	2713.4 (1770.9-3864)	9758.1 (7088.2-13097.5)	259.6 (162.7-424.8)	89.2 (52.1-137.6)	425 (263.6-639.9)	376.4 (232.2-621.3)	2802.6 (1830.6-3999.6)	10183.1 (7403.2-13688.7)	263.3 (166.2-428.6)

[1]Years of Life Lost

[2]Years Lived with Disability

[3]Disability-Adjusted Life Years

Supplementary Table 5

Location		YLLs[1]			YLDs[2]			DALYs[3]		
		1990 (95% UI)	2019 (95% UI)	Percent changes (95% UI)	1990 (95% UI)	2019 (95% UI)	Percent changes (95% UI)	1990 (95% UI)	2019 (95% UI)	Percent changes (95% UI)
**North Africa and Middle East**		10166.6 (4379.1-16031.6)	22421.7 (10204.8-35816.3)	120.5 (75.5-180.3)	457.2 (185.8-772.6)	2198.8 (946.8-3714.7)	380.9 (273.7-514.3)	10623.8 (4591.1-16900)	24620.5 (11060.4-39136.1)	131.7 (84-196.6)
**Countries**	Afghanistan	183 (71.3-329.6)	358.9 (141.7-628.7)	96.1 (34.8-179.9)	4.5 (1.7-8.7)	10.8 (4.1-19.8)	142.5 (63-273.8)	187.5 (73-336.5)	369.7 (146.4-648)	97.2 (35.6-181.2)
	Algeria	700.3 (302.7-1160.8)	1484.7 (651.6-2507.2)	112 (47.2-208.9)	30.7 (12.5-54.2)	121.4 (51-217.6)	295.5 (162.5-479.3)	731 (315.5-1204.5)	1606.1 (705.3-2703.7)	119.7 (53-216)
	Bahrain	12.2 (5.2-20.7)	43.1 (17.3-75.5)	251.8 (131-421.3)	0.7 (0.3-1.2)	6 (2.4-11.5)	808.8 (450.3-1292.2)	12.9 (5.5-21.7)	49 (19.5-86.4)	280.2 (148-468.2)
	Egypt	935.7 (416.7-1445.9)	2826.1 (1257.5-4937.2)	202 (110.2-335.2)	36.9 (15.4-64.2)	209.3 (83.5-384.4)	467.3 (273.2-755.3)	972.6 (431.9-1503.9)	3035.4 (1381.1-5295.1)	212.1 (117-347.8)
	Iran (Islamic Republic of)	1266.3 (516.8-2046.9)	3343.1 (1500.3-5499.9)	164 (100.4-253.6)	76.9 (32.1-137.1)	383.7 (158.2-664.1)	398.8 (268.1-570.1)	1343.3 (552.3-2170.8)	3726.8 (1665-6117.9)	177.4 (111.8-269.7)
	Iraq	446.6 (176.9-741.3)	1234 (510.7-2059.5)	176.3 (91.5-307.2)	18.9 (7.4-33.1)	93.1 (37.8-165.7)	391.9 (222.2-667.9)	465.5 (183.4-770.5)	1327.1 (546.3-2211.7)	185.1 (98.4-319.8)
	Jordan	94.6 (40-156.1)	400.6 (184.3-674.5)	323.3 (175.6-542.2)	4.8 (1.8-8.5)	43.5 (17.6-77.6)	803.5 (472.7-1311)	99.4 (41.8-164.6)	444.1 (202.4-741.7)	346.5 (188.9-579.8)
	Kuwait	27.2 (11.3-45.1)	106.1 (47.8-184.4)	290.1 (184.6-432.5)	2.9 (1.2-5.2)	18.3 (7.7-34)	524.1 (337.5-809.3)	30.1 (12.7-49.8)	124.5 (53.3-214.2)	312.9 (199.3-463.2)
	Lebanon	255.6 (113.8-422.9)	784.2 (330.4-1424.5)	206.9 (103.4-359.9)	14.3 (5.9-25.4)	100.9 (39.3-187)	606.7 (358.3-976.4)	269.8 (119.5-444.1)	885.1 (375.3-1610.7)	228 (118.2-385.4)
	Libya	99.6 (38.2-169.6)	233.1 (100.6-380.4)	134.1 (56.2-273.5)	5.3 (1.9-9.3)	20.3 (8.2-36.6)	285 (141.7-556.2)	104.8 (40.7-178.3)	253.4 (108.2-416.6)	141.7 (61.2-288.4)
	Morocco	450.5 (199.9-743.3)	979.7 (398.9-1633)	117.5 (46.7-199.2)	16.6 (6.9-29.3)	63.6 (23.8-113.4)	284.3 (150.4-463.7)	467 (205.3-770)	1043.3 (422.6-1751)	123.4 (50.7-208.2)
	Oman	22.5 (8.7-38.5)	38.4 (16.3-65.5)	71.2 (18.5-155.4)	1.5 (0.6-2.6)	6.2 (2.4-11.5)	320.5 (172.6-530.2)	23.9 (9.2-41)	44.6 (19.4-77.2)	86.4 (29.2-177.1)
	Palestine	82.3 (33.3-148.6)	181.2 (80.2-306.1)	120.1 (46.7-244.8)	3.9 (1.5-7.5)	15.4 (6.2-26.8)	289.6 (160.3-530.1)	86.3 (34.8-155.3)	196.6 (86.5-329.2)	127.9 (52.5-257.3)
	Qatar	4.3 (1.8-7.8)	35.3 (13.6-64.5)	713.2 (423.1-1159.9)	0.3 (0.1-0.6)	7.3 (2.7-15.1)	2347 (1333.3-3964.7)	4.6 (1.9-8.4)	42.6 (16.7-79)	818.7 (483.8-1311.1)
	Saudi Arabia	158.3 (42.5-320.5)	457.4 (187.6-834.2)	188.9 (66-606.6)	7.7 (2-15.7)	75 (30.1-143.5)	874.4 (418.9-2252.2)	166 (44.5-336)	532.4 (220.1-977.6)	220.7 (82.3-683.1)
	Sudan	390.8 (133.3-722.9)	850.3 (308.5-1605.1)	117.6 (48.6-221.9)	12.1 (4.1-23.2)	41.7 (14-81.6)	244.3 (119.7-428.9)	402.9 (136.8-744.3)	892 (323.8-1674.5)	121.4 (51.9-227.6)
	Syrian Arab Republic	341.6 (138.8-587.7)	637.9 (264.8-1145.1)	86.7 (17.6-207.1)	16.2 (6.7-28.8)	61.8 (24.8-117.3)	281.9 (128.2-536.5)	357.8 (144.9-619.4)	699.7 (289.7-1248.3)	95.6 (23.5-220.2)
	Tunisia	284.2 (114.4-472.3)	650.7 (270.8-1138.8)	129 (45.5-262.8)	14.8 (5.8-26.2)	70.2 (27.3-129.4)	373.6 (195.5-647.3)	299 (119.3-491.2)	721 (301-1267.7)	141.1 (53.8-281.2)
	Turkey	4161.6 (1764.1-6894.6)	6839.7 (2981-11450.6)	64.4 (13-136.7)	179.3 (70.9-314.8)	792.8 (326.8-1414.9)	342.2 (195.3-569.6)	4340.8 (1839.4-7203.4)	7632.4 (3358.6-12727.4)	75.8 (20.7-153.6)
	United Arab Emirates	17.8 (7.1-34.4)	188 (68.8-401.8)	957.2 (546-1547.9)	1 (0.4-2.1)	22 (7.1-48.9)	2010.3 (1076.8-3239.2)	18.8 (7.5-36.2)	210 (76.3-448)	1015.6 (585.4-1650.2)
	Yemen	224.8 (88.9-400.4)	726.4 (292.9-1229.9)	223.1 (127.9-386.1)	7.6 (2.9-14.3)	33.1 (12.9-59.7)	334.9 (190.6-577.5)	232.4 (92.2-414.9)	759.5 (305.2-1285.6)	226.8 (130.5-393.9)

[1]Years of Life Lost

[2]Years Lived with Disability

[3]Disability-Adjusted Life Years

Supplementary Figure 1

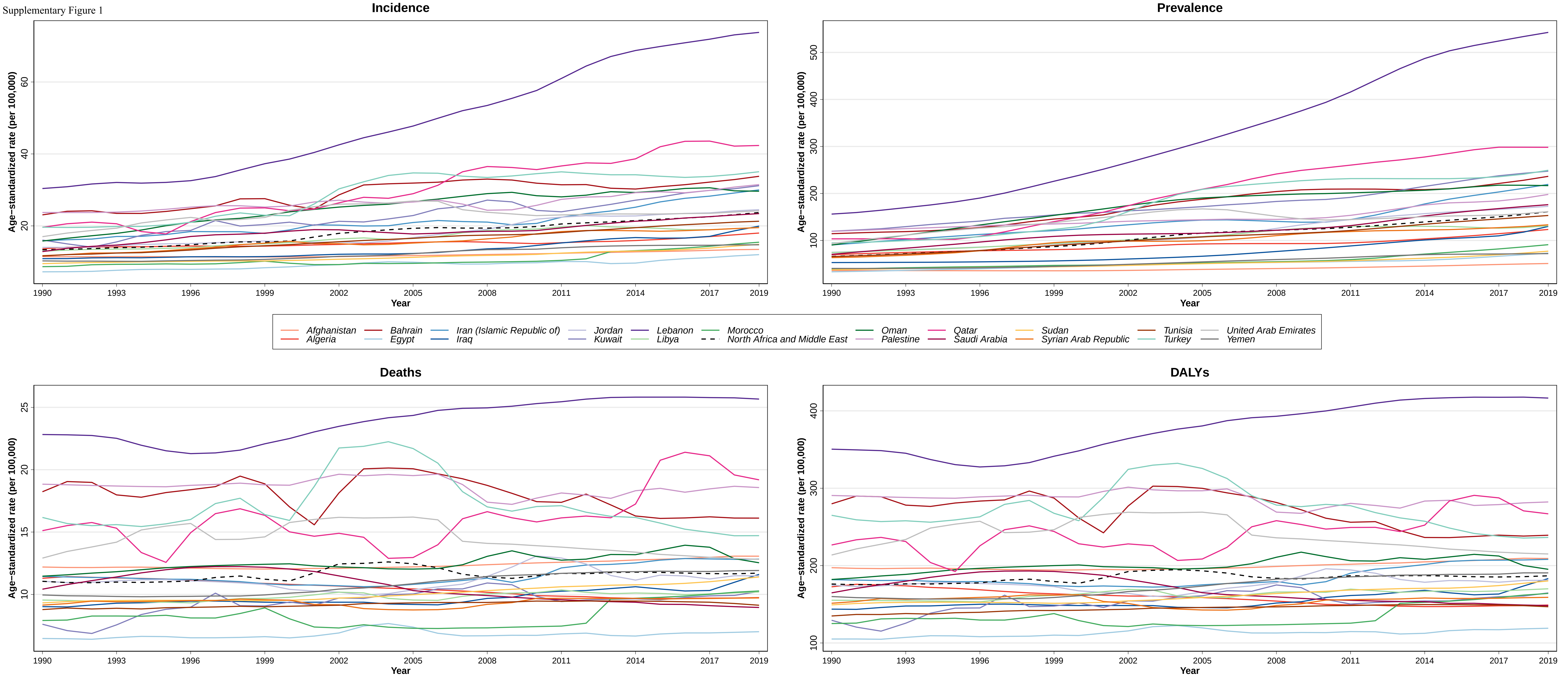

Supplementary Figure 2

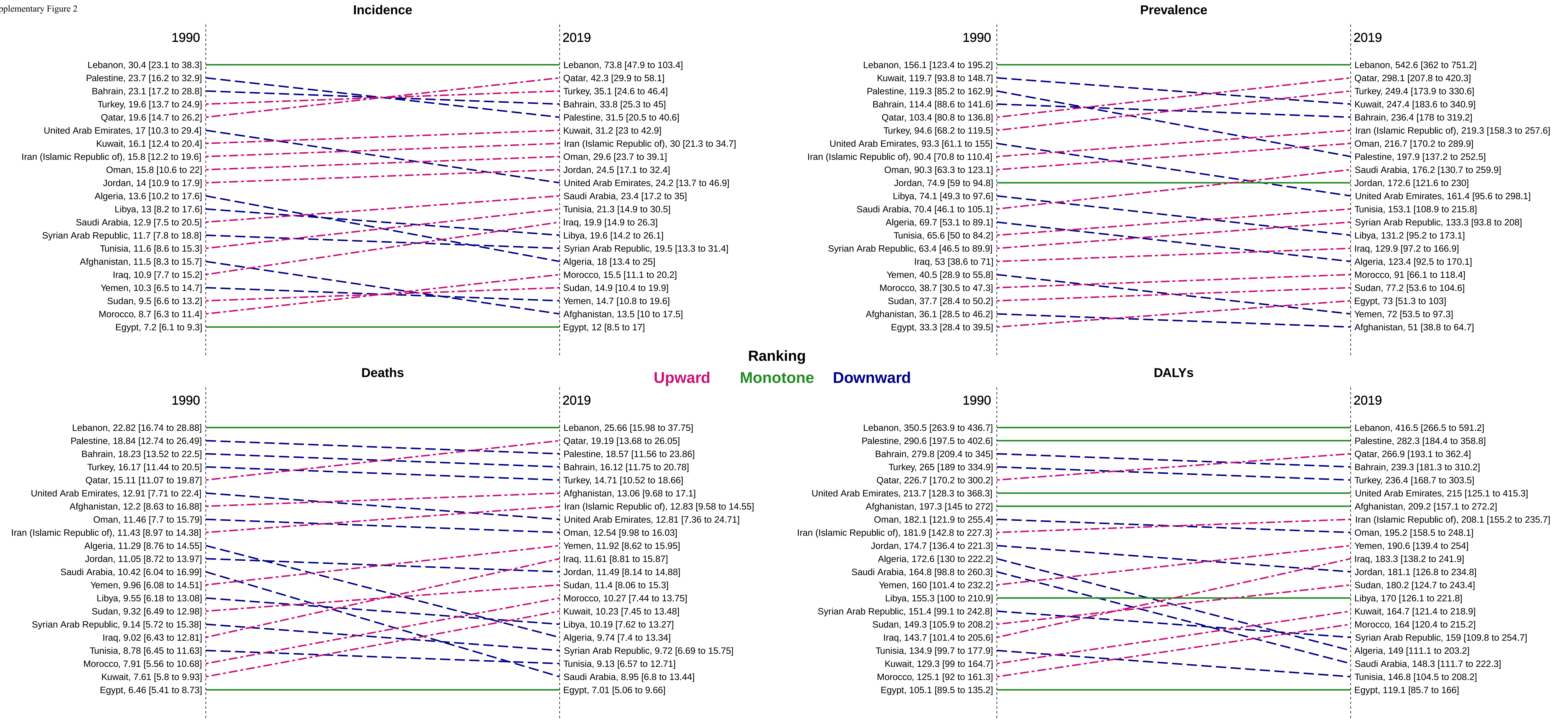

Supplementary Figure 3

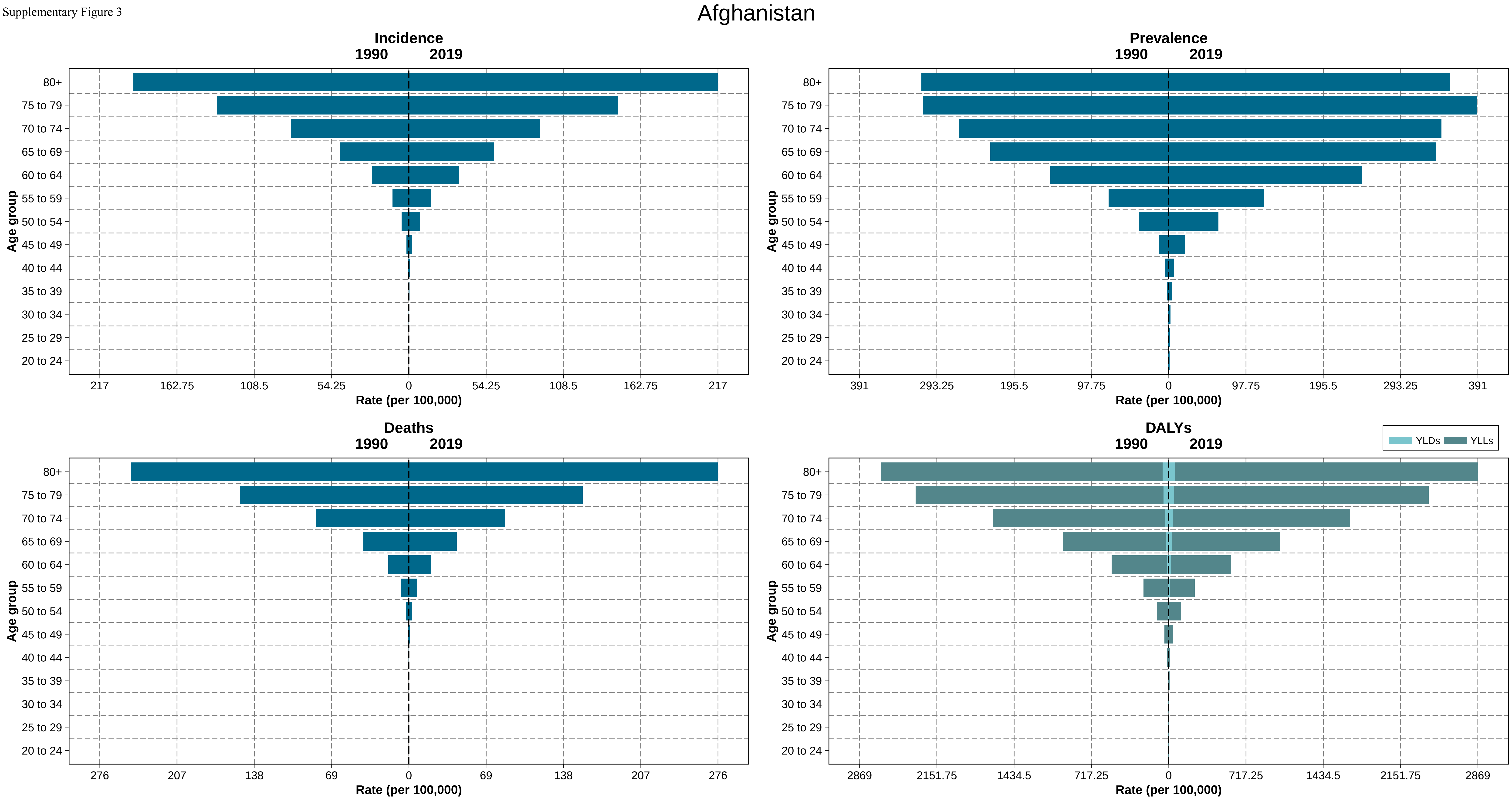

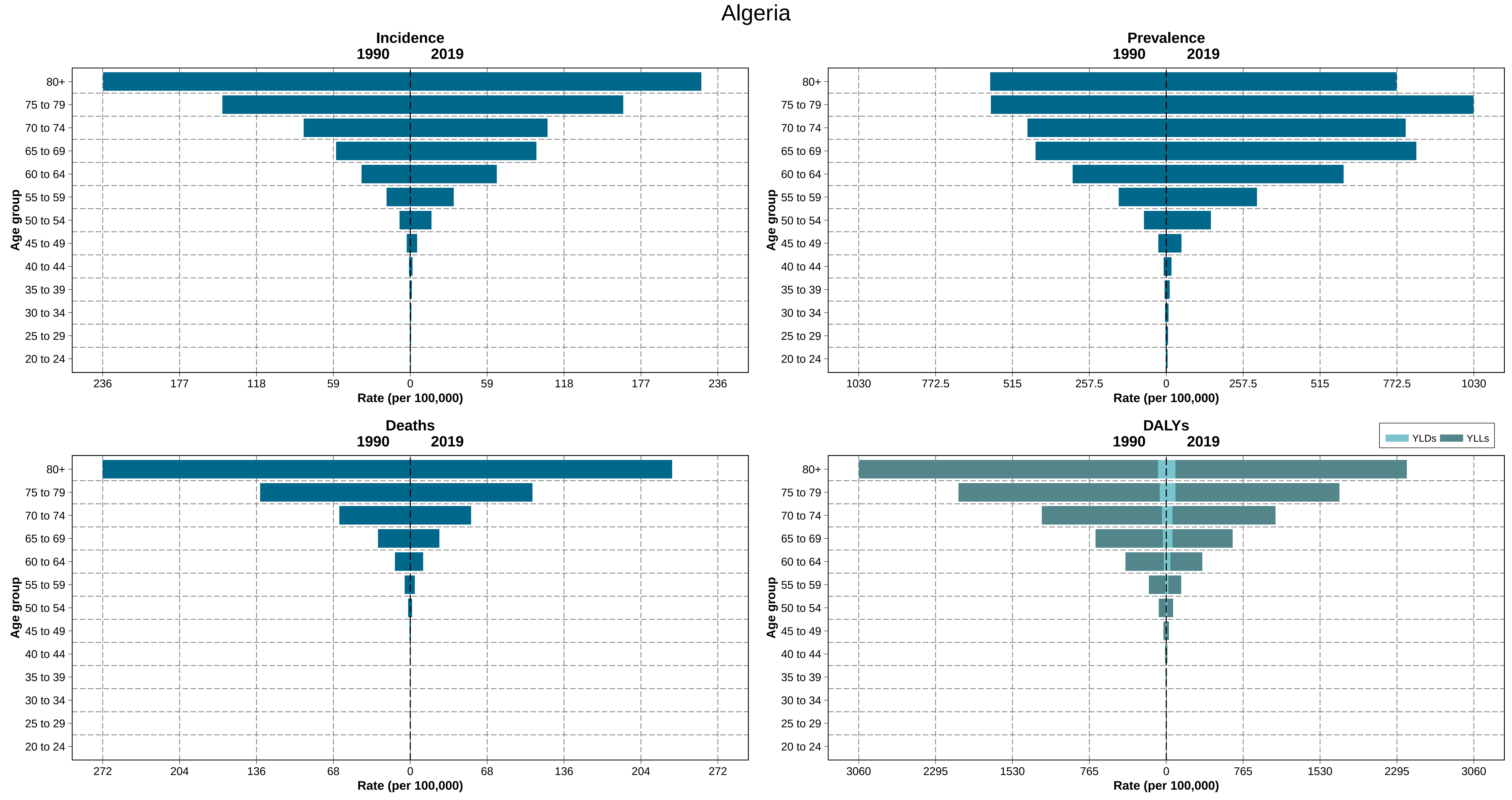
Algeria
Incidence
1990
2019
Prevalence
1990
2019
Deaths
1990
2019
DALYs
1990
2019
YLDs
YLLs
Age group
80+
75 to 79
70 to 74
65 to 69
60 to 64
55 to 59
50 to 54
45 to 49
40 to 44
35 to 39
30 to 34
25 to 29
20 to 24
236 177 118 59 0 59 118 177 236
1030 772.5 515 257.5 0 257.5 515 772.5 1030
272 204 136 68 0 68 136 204 272
3060 2295 1530 765 0 765 1530 2295 3060
Rate (per 100,000)

Bahrain

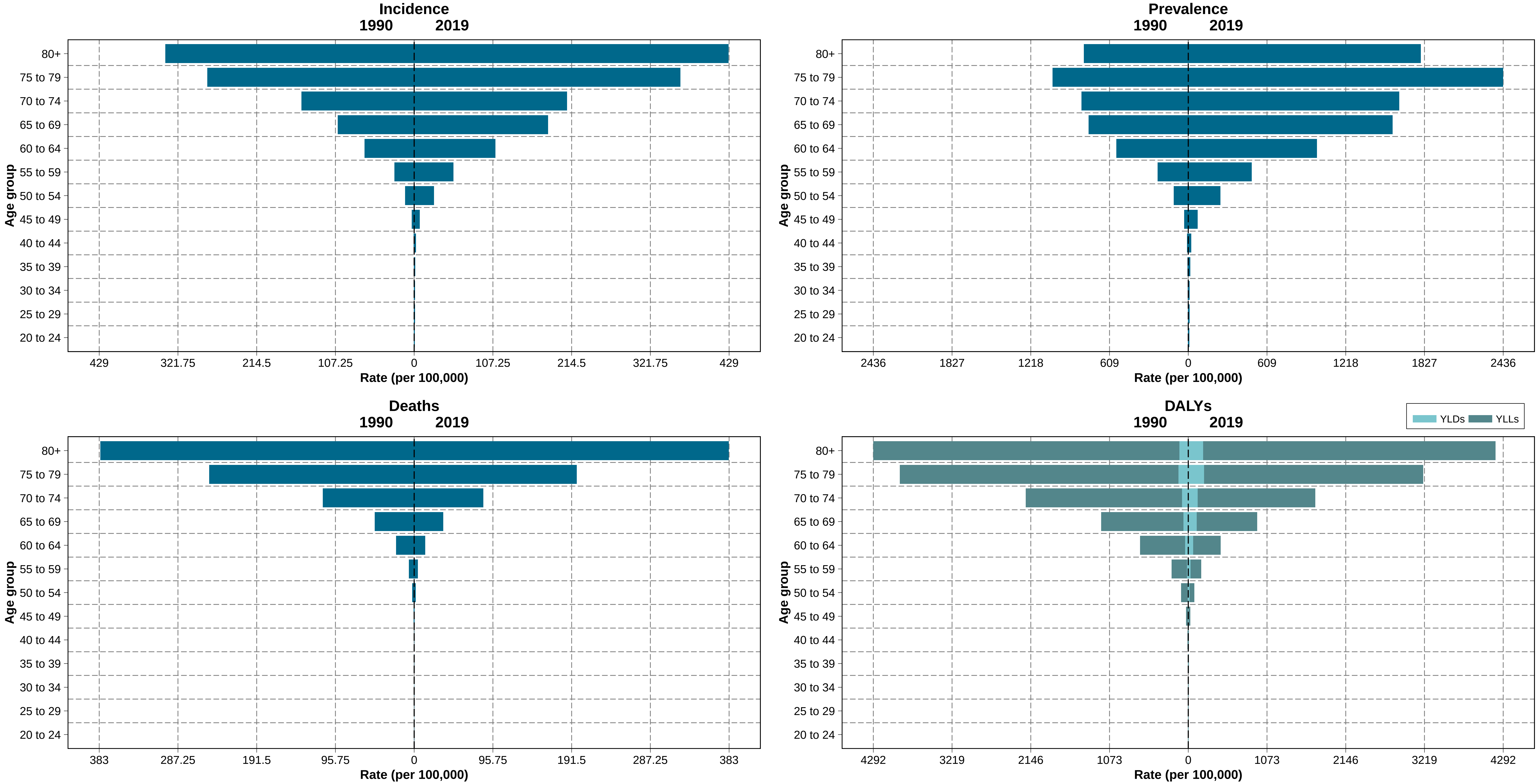

Incidence
1990
2019
Prevalence
1990
2019
Deaths
1990
2019
DALYs
1990
2019
YLDs
YLLs
80+
75 to 79
70 to 74
65 to 69
60 to 64
55 to 59
50 to 54
45 to 49
40 to 44
35 to 39
30 to 34
25 to 29
20 to 24
Age group
429
321.75
214.5
107.25
0
2436
1827
1218
609
383
287.25
191.5
95.75
4292
3219
2146
1073
Rate (per 100,000)

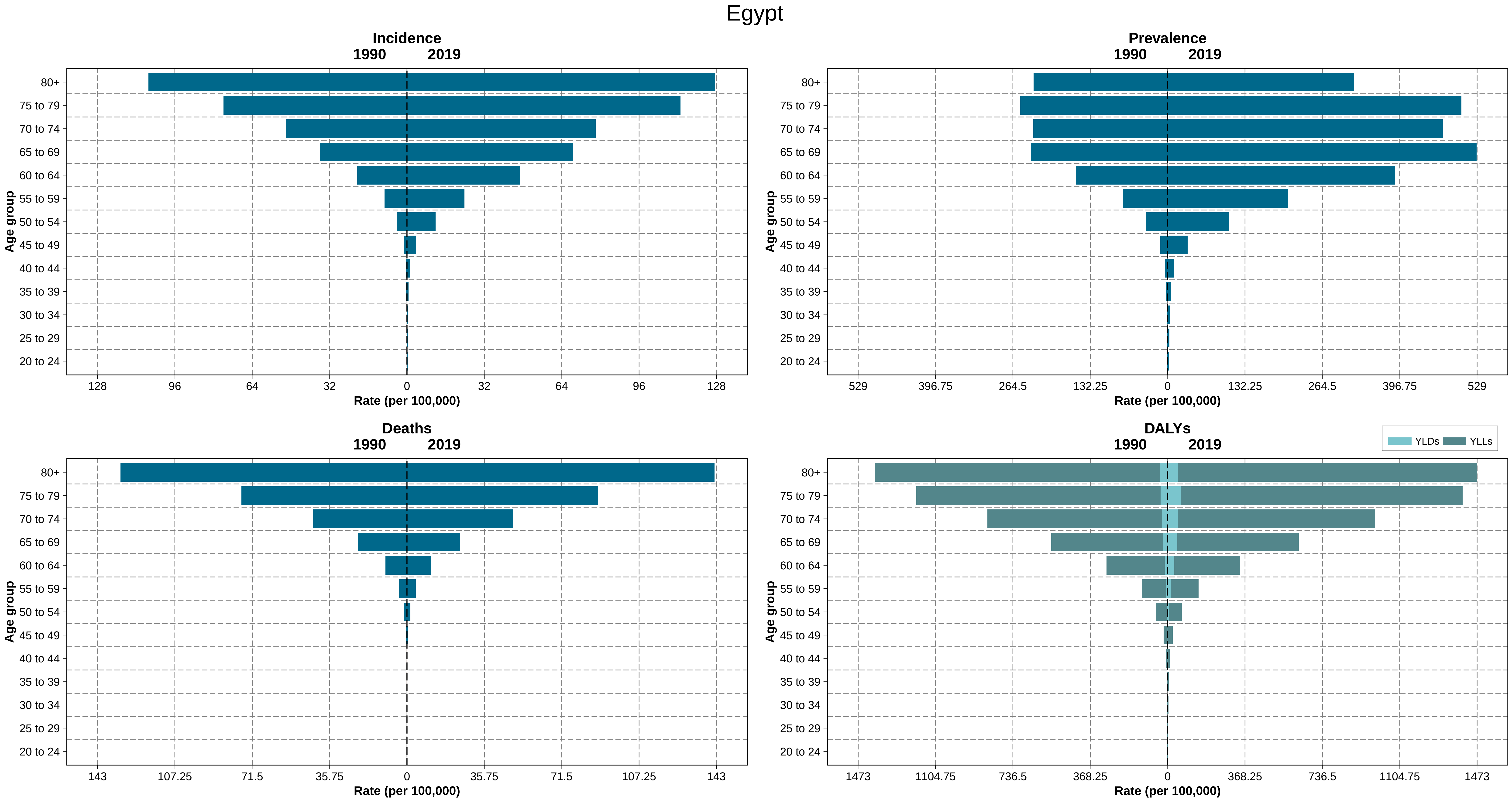

Egypt
Incidence
1990
2019
Prevalence
1990
2019
Deaths
1990
2019
DALYs
1990
2019
YLDs
YLLs
Age group
80+
75 to 79
70 to 74
65 to 69
60 to 64
55 to 59
50 to 54
45 to 49
40 to 44
35 to 39
30 to 34
25 to 29
20 to 24
128 96 64 32 0 32 64 96 128
529 396.75 264.5 132.25 0 132.25 264.5 396.75 529
143 107.25 71.5 35.75 0 35.75 71.5 107.25 143
1473 1104.75 736.5 368.25 0 368.25 736.5 1104.75 1473
Rate (per 100,000)

Iran (Islamic Republic of)

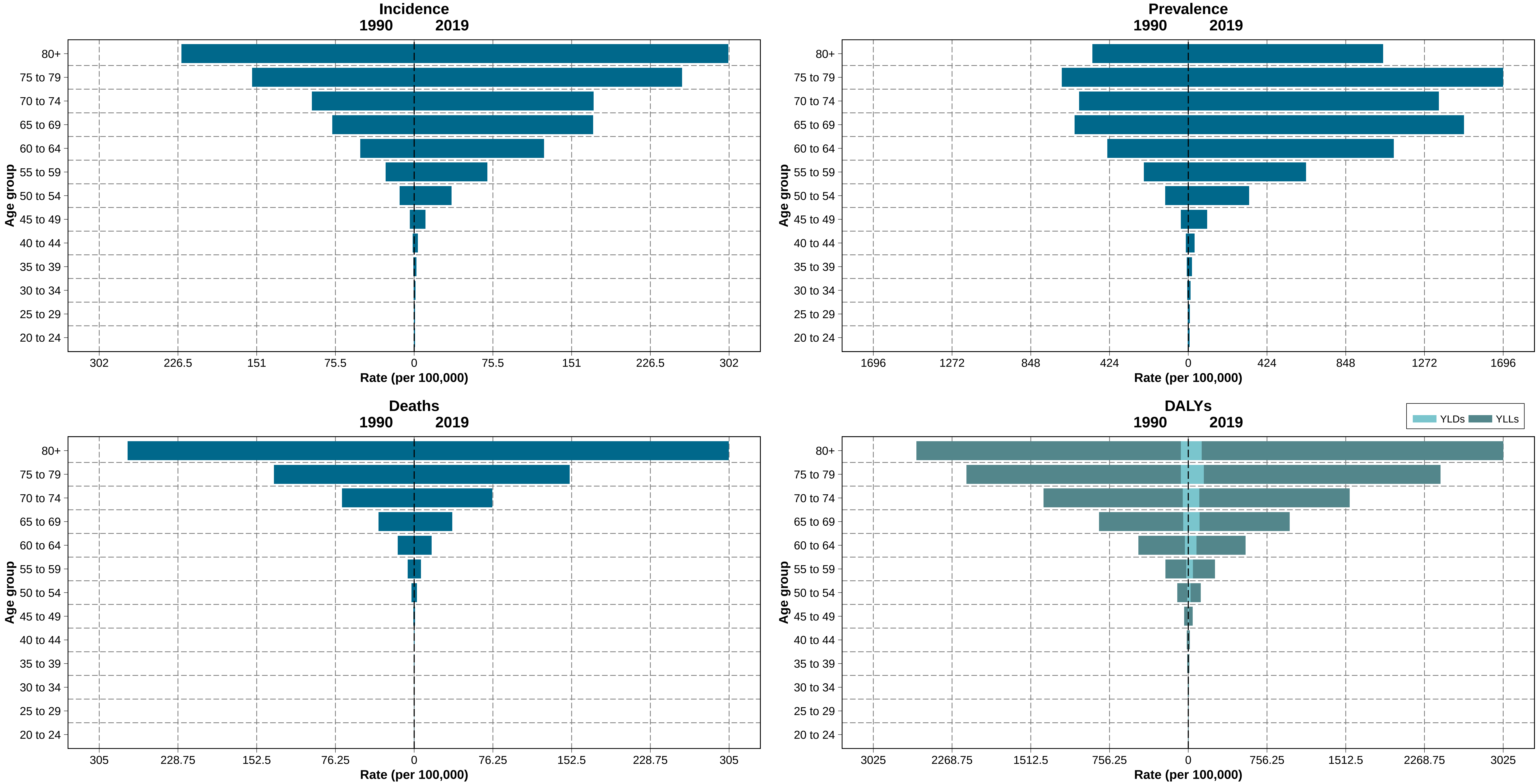

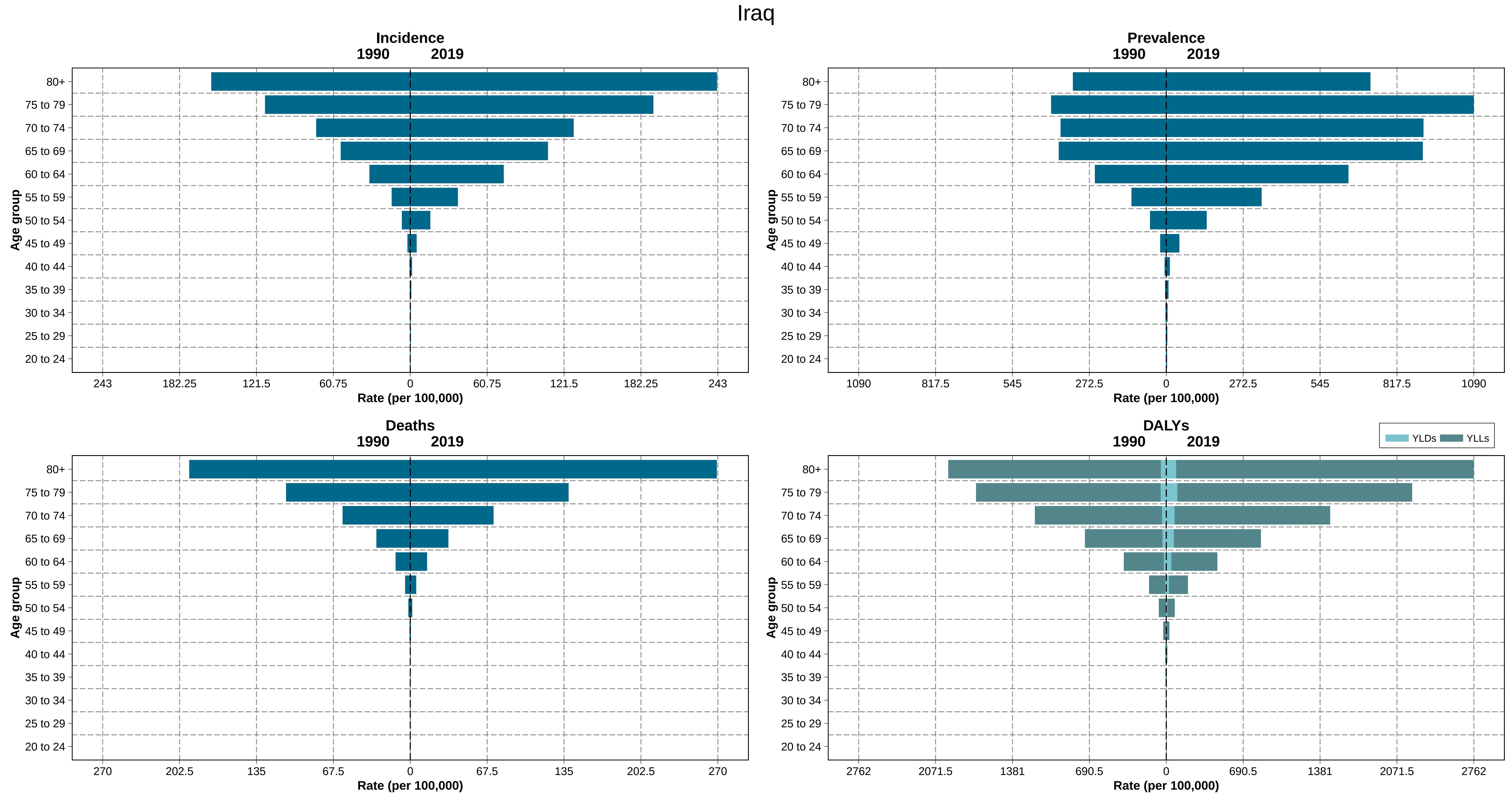

Iraq
Incidence
1990
2019
80+
75 to 79
70 to 74
65 to 69
60 to 64
55 to 59
50 to 54
45 to 49
40 to 44
35 to 39
30 to 34
25 to 29
20 to 24
Age group
243
182.25
121.5
60.75
0
60.75
121.5
182.25
243
Rate (per 100,000)
Prevalence
1990
2019
1090
817.5
545
272.5
0
272.5
545
817.5
1090
Rate (per 100,000)
Deaths
1990
2019
270
202.5
135
67.5
0
67.5
135
202.5
270
Rate (per 100,000)
DALYs
1990
2019
YLDs
YLLs
2762
2071.5
1381
690.5
0
690.5
1381
2071.5
2762
Rate (per 100,000)

Jordan

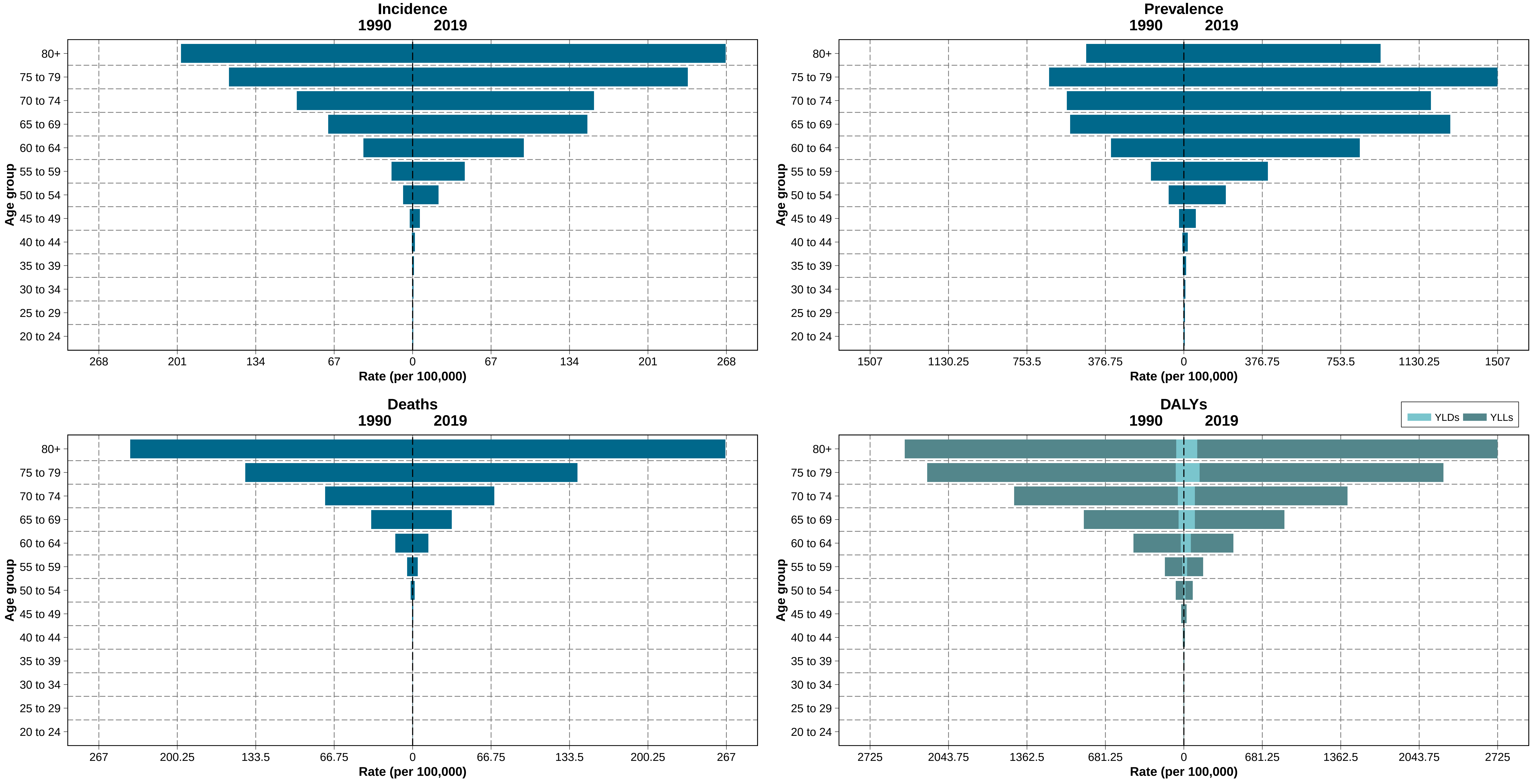

Incidence
1990
2019
80+
75 to 79
70 to 74
65 to 69
60 to 64
55 to 59
50 to 54
45 to 49
40 to 44
35 to 39
30 to 34
25 to 29
20 to 24
Age group
268
201
134
67
0
67
134
201
268
Rate (per 100,000)
Prevalence
1990
2019
1507
1130.25
753.5
376.75
0
376.75
753.5
1130.25
1507
Rate (per 100,000)
Deaths
1990
2019
267
200.25
133.5
66.75
0
66.75
133.5
200.25
267
Rate (per 100,000)
DALYs
1990
2019
YLDs
YLLs
2725
2043.75
1362.5
681.25
0
681.25
1362.5
2043.75
2725
Rate (per 100,000)

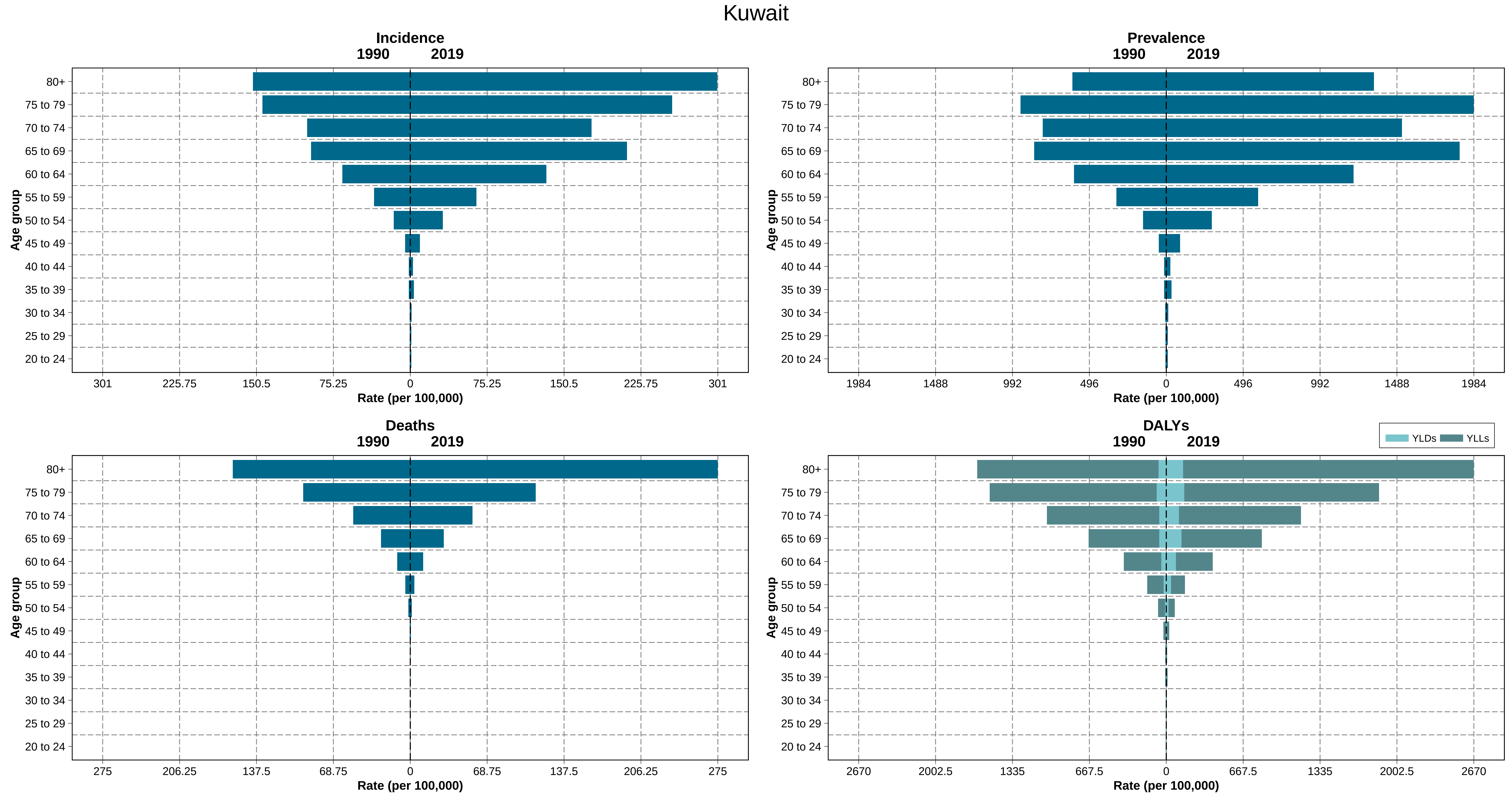
Kuwait
Incidence
1990
2019
Prevalence
1990
2019
Deaths
1990
2019
DALYs
1990
2019
YLDs
YLLs
80+
75 to 79
70 to 74
65 to 69
60 to 64
55 to 59
50 to 54
45 to 49
40 to 44
35 to 39
30 to 34
25 to 29
20 to 24
Age group
301
225.75
150.5
75.25
0
75.25
150.5
225.75
301
1984
1488
992
496
0
496
992
1488
1984
275
206.25
137.5
68.75
0
68.75
137.5
206.25
275
2670
2002.5
1335
667.5
0
667.5
1335
2002.5
2670
Rate (per 100,000)

Lebanon

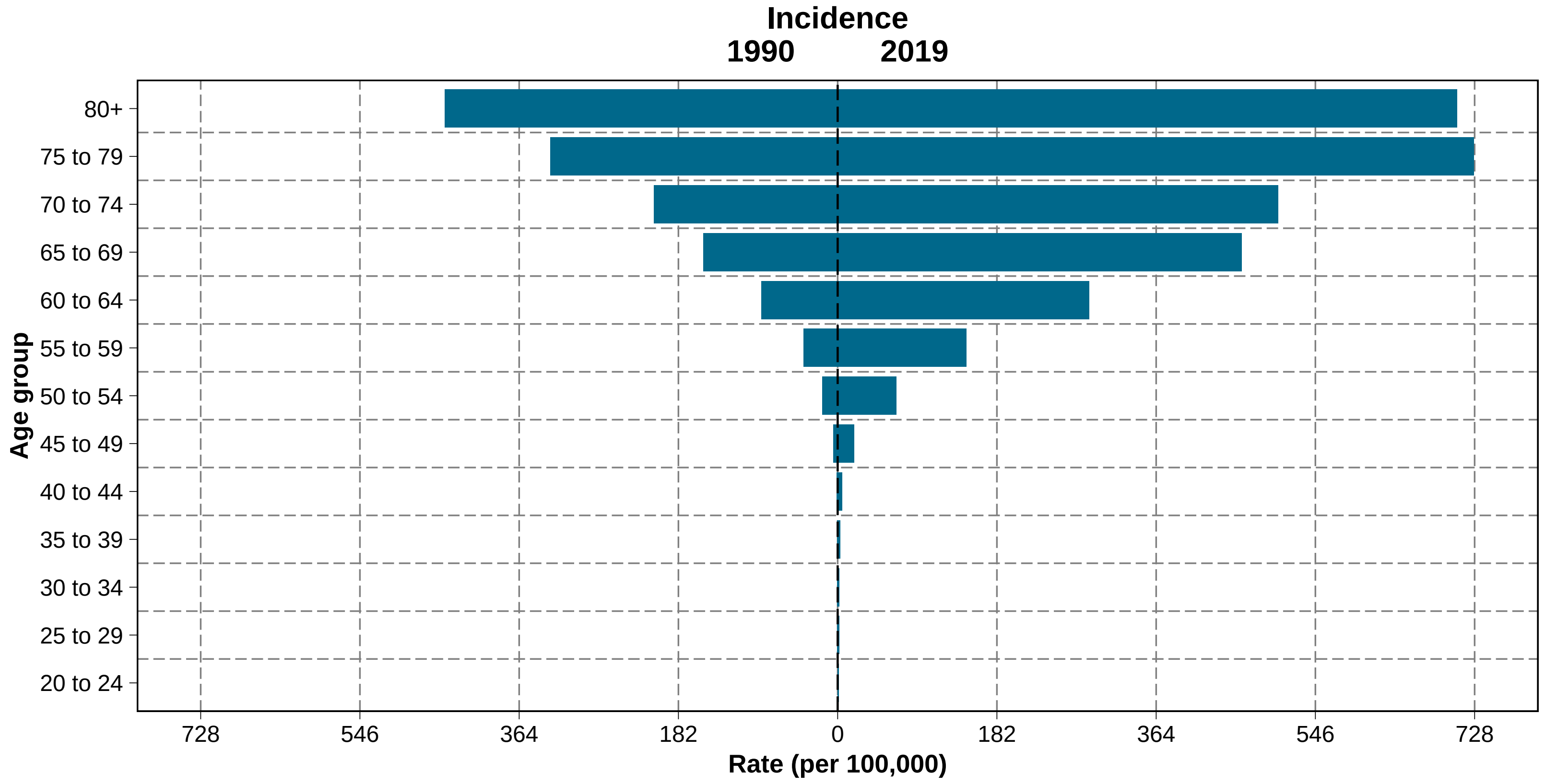

Incidence
1990
2019
80+
75 to 79
70 to 74
65 to 69
60 to 64
55 to 59
50 to 54
45 to 49
40 to 44
35 to 39
30 to 34
25 to 29
20 to 24
Age group
728
546
364
182
0
182
364
546
728
Rate (per 100,000)

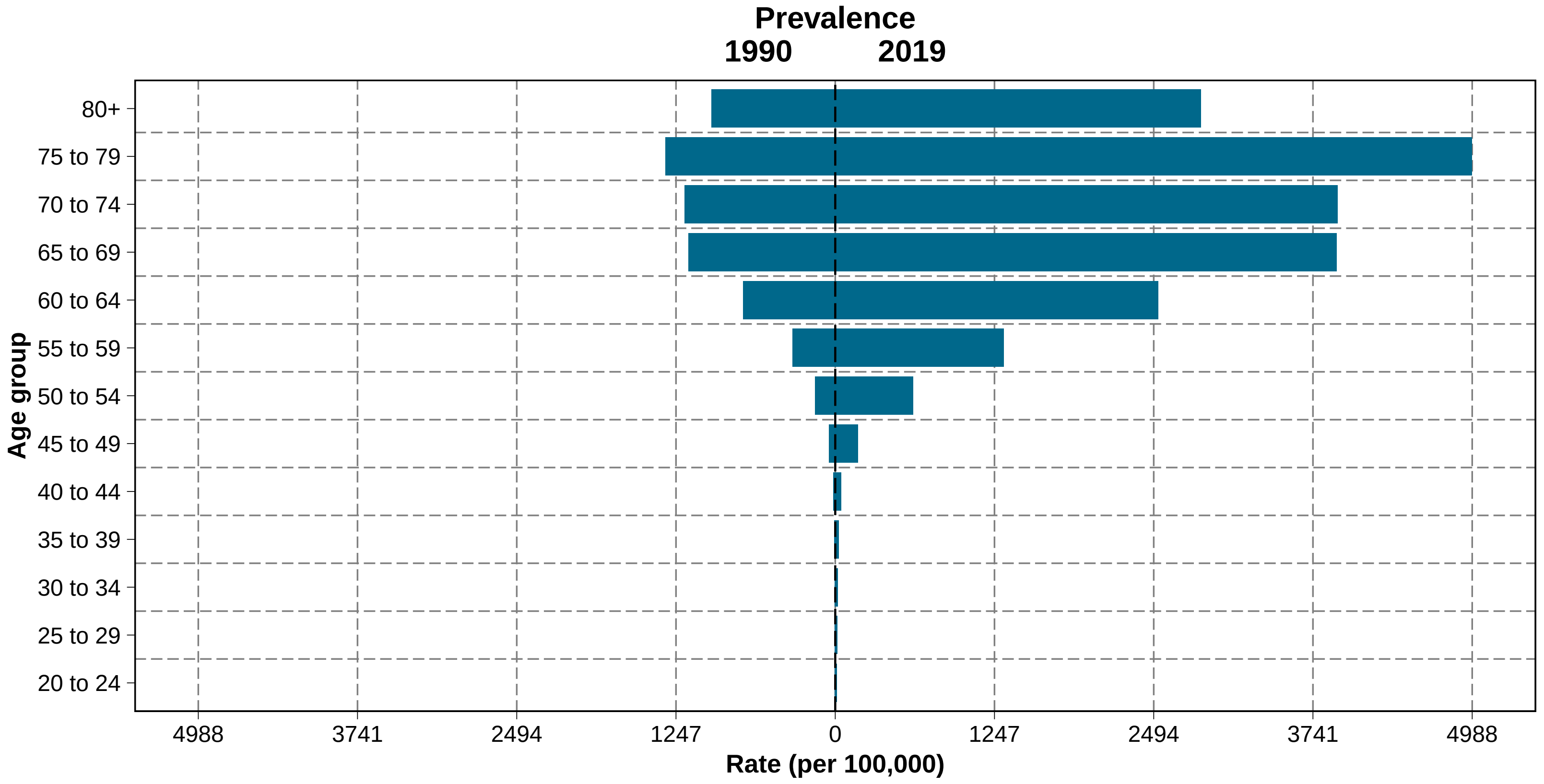

Prevalence
1990
2019
80+
75 to 79
70 to 74
65 to 69
60 to 64
55 to 59
50 to 54
45 to 49
40 to 44
35 to 39
30 to 34
25 to 29
20 to 24
Age group
4988
3741
2494
1247
0
1247
2494
3741
4988
Rate (per 100,000)

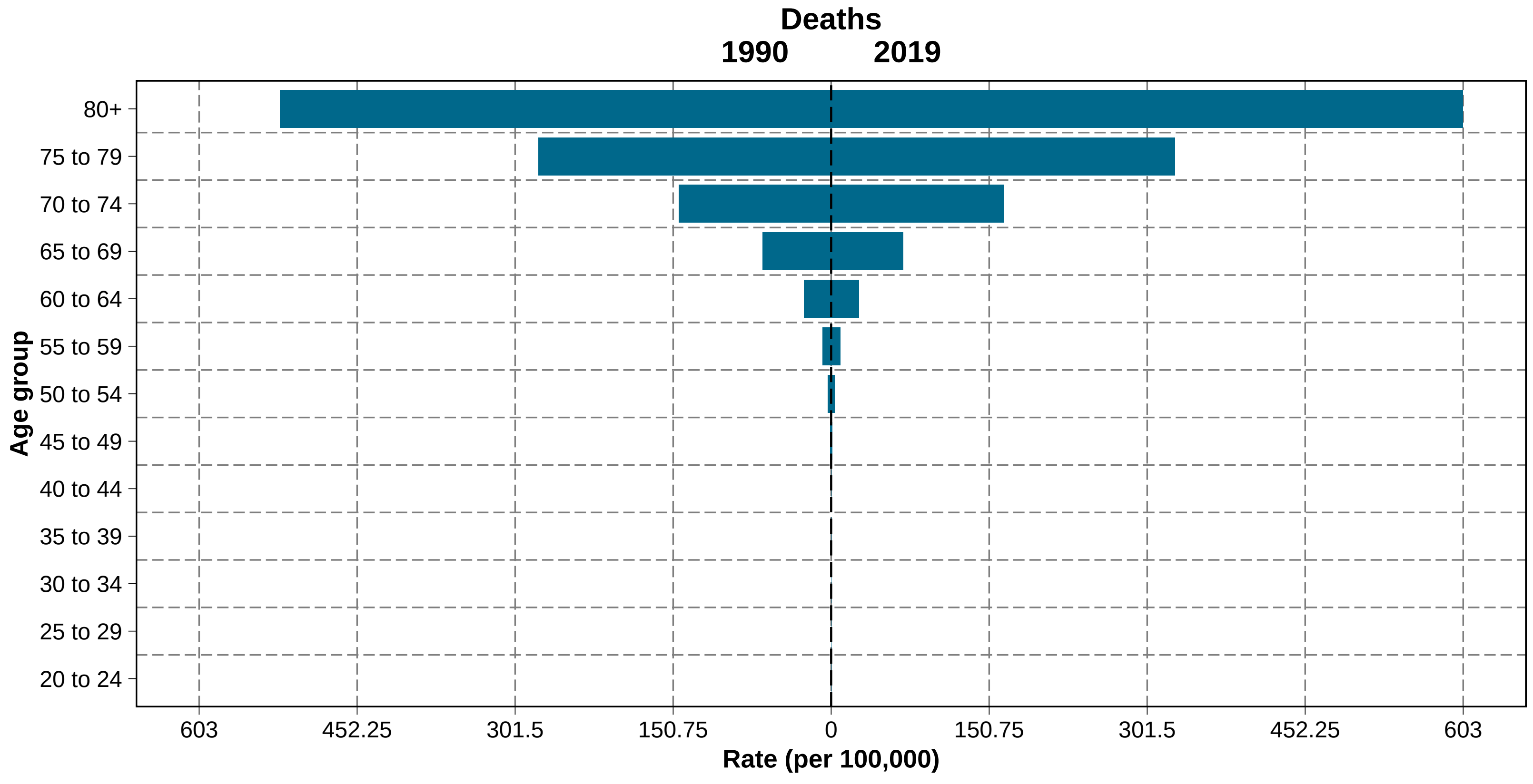

Deaths
1990
2019
80+
75 to 79
70 to 74
65 to 69
60 to 64
55 to 59
50 to 54
45 to 49
40 to 44
35 to 39
30 to 34
25 to 29
20 to 24
Age group
603
452.25
301.5
150.75
0
150.75
301.5
452.25
603
Rate (per 100,000)

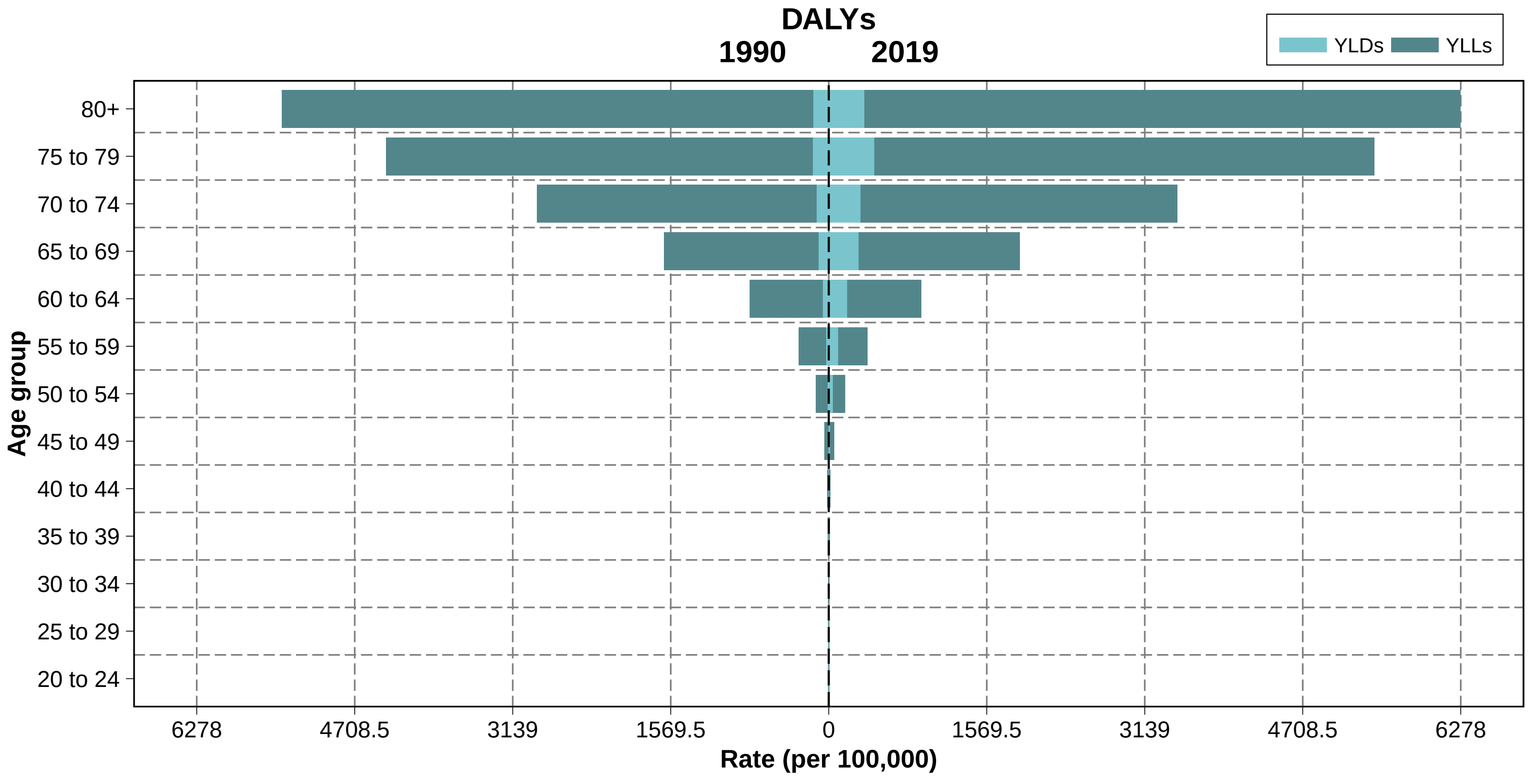

DALYs
1990
2019
YLDs
YLLs
80+
75 to 79
70 to 74
65 to 69
60 to 64
55 to 59
50 to 54
45 to 49
40 to 44
35 to 39
30 to 34
25 to 29
20 to 24
Age group
6278
4708.5
3139
1569.5
0
1569.5
3139
4708.5
6278
Rate (per 100,000)

Libya

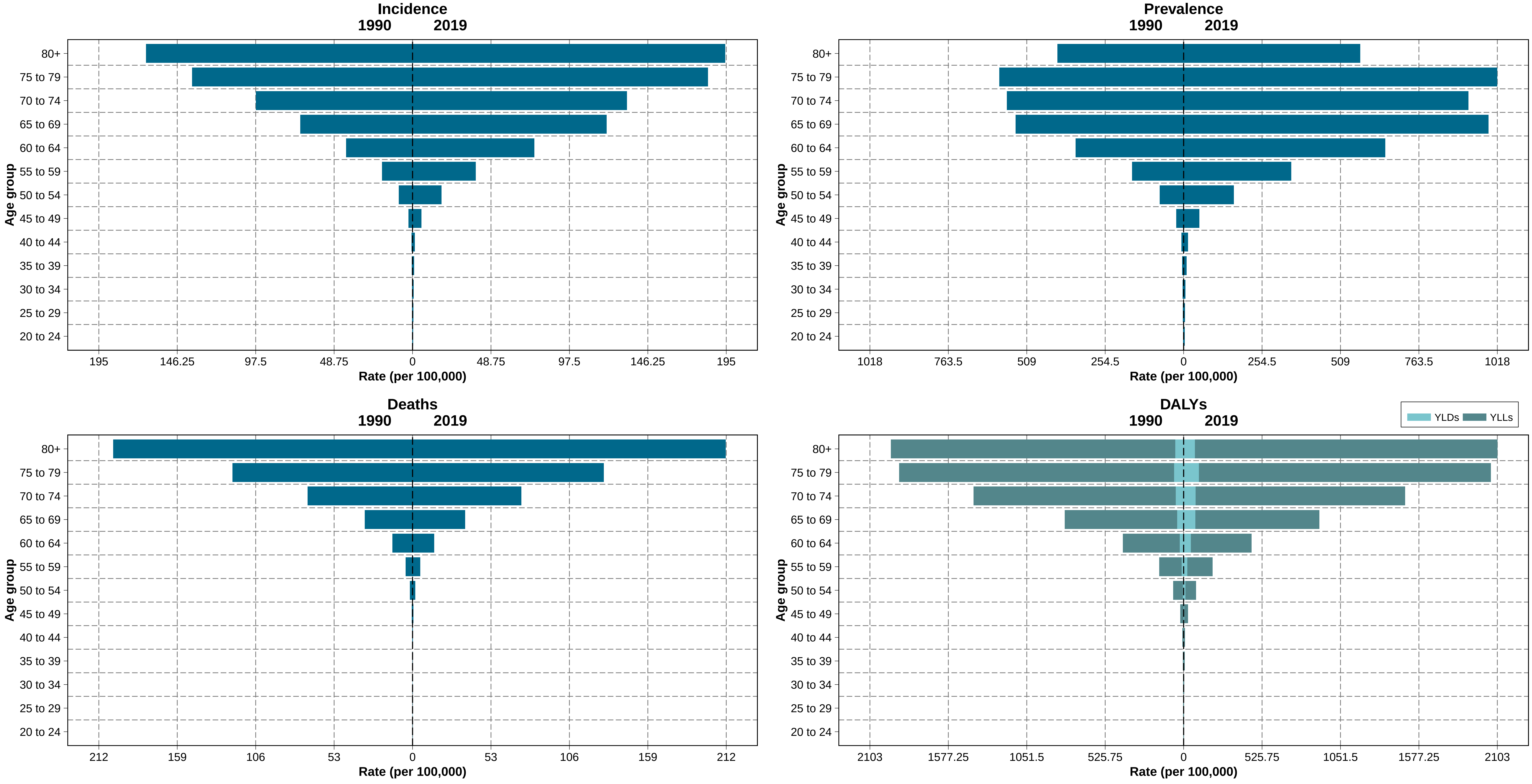
Incidence
1990
2019
Prevalence
1990
2019
Deaths
1990
2019
DALYs
1990
2019
YLDs
YLLs
Age group
80+
75 to 79
70 to 74
65 to 69
60 to 64
55 to 59
50 to 54
45 to 49
40 to 44
35 to 39
30 to 34
25 to 29
20 to 24
Rate (per 100,000)
195 146.25 97.5 48.75 0 48.75 97.5 146.25 195
1018 763.5 509 254.5 0 254.5 509 763.5 1018
212 159 106 53 0 53 106 159 212
2103 1577.25 1051.5 525.75 0 525.75 1051.5 1577.25 2103

Morocco

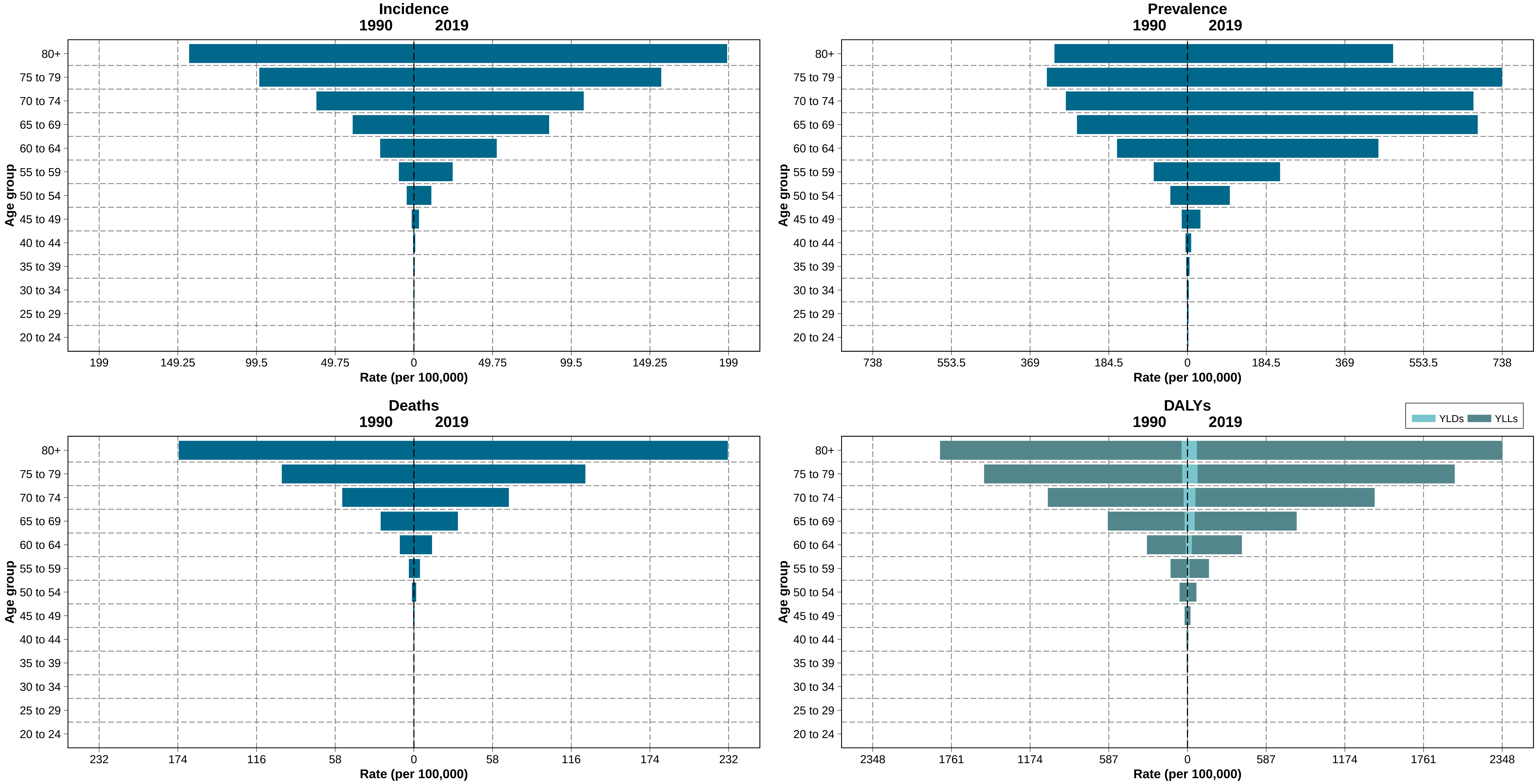

Incidence
1990
2019
80+
75 to 79
70 to 74
65 to 69
60 to 64
55 to 59
50 to 54
45 to 49
40 to 44
35 to 39
30 to 34
25 to 29
20 to 24
Age group
199
149.25
99.5
49.75
0
49.75
99.5
149.25
199
Rate (per 100,000)
Prevalence
1990
2019
738
553.5
369
184.5
0
184.5
369
553.5
738
Rate (per 100,000)
Deaths
1990
2019
232
174
116
58
0
58
116
174
232
Rate (per 100,000)
DALYs
1990
2019
YLDs
YLLs
2348
1761
1174
587
0
587
1174
1761
2348
Rate (per 100,000)

Oman

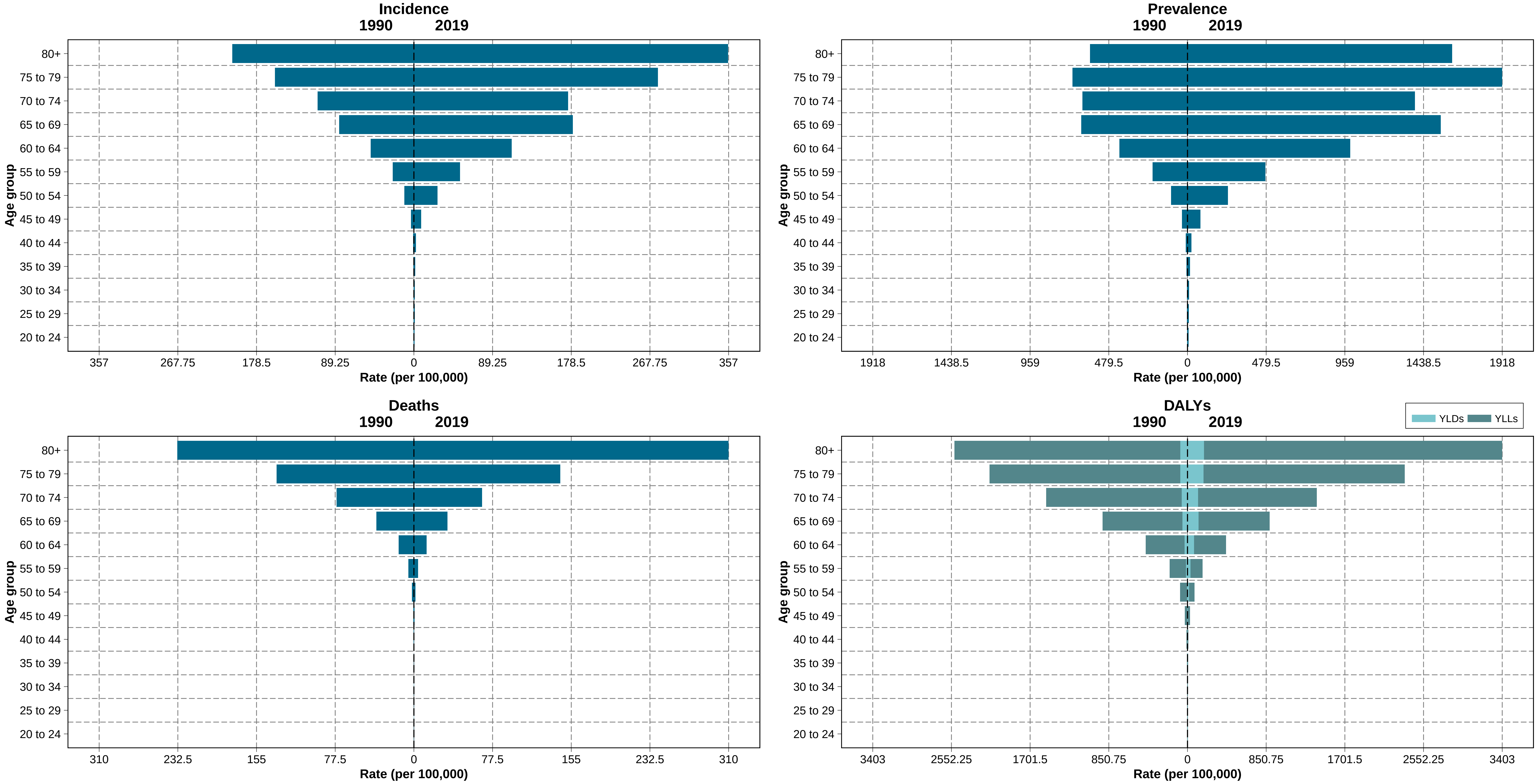
Incidence
1990
2019
Prevalence
1990
2019
Deaths
1990
2019
DALYs
1990
2019
YLDs
YLLs
Age group
80+
75 to 79
70 to 74
65 to 69
60 to 64
55 to 59
50 to 54
45 to 49
40 to 44
35 to 39
30 to 34
25 to 29
20 to 24
357
267.75
178.5
89.25
0
89.25
178.5
267.75
357
1918
1438.5
959
479.5
0
479.5
959
1438.5
1918
310
232.5
155
77.5
0
77.5
155
232.5
310
3403
2552.25
1701.5
850.75
0
850.75
1701.5
2552.25
3403
Rate (per 100,000)

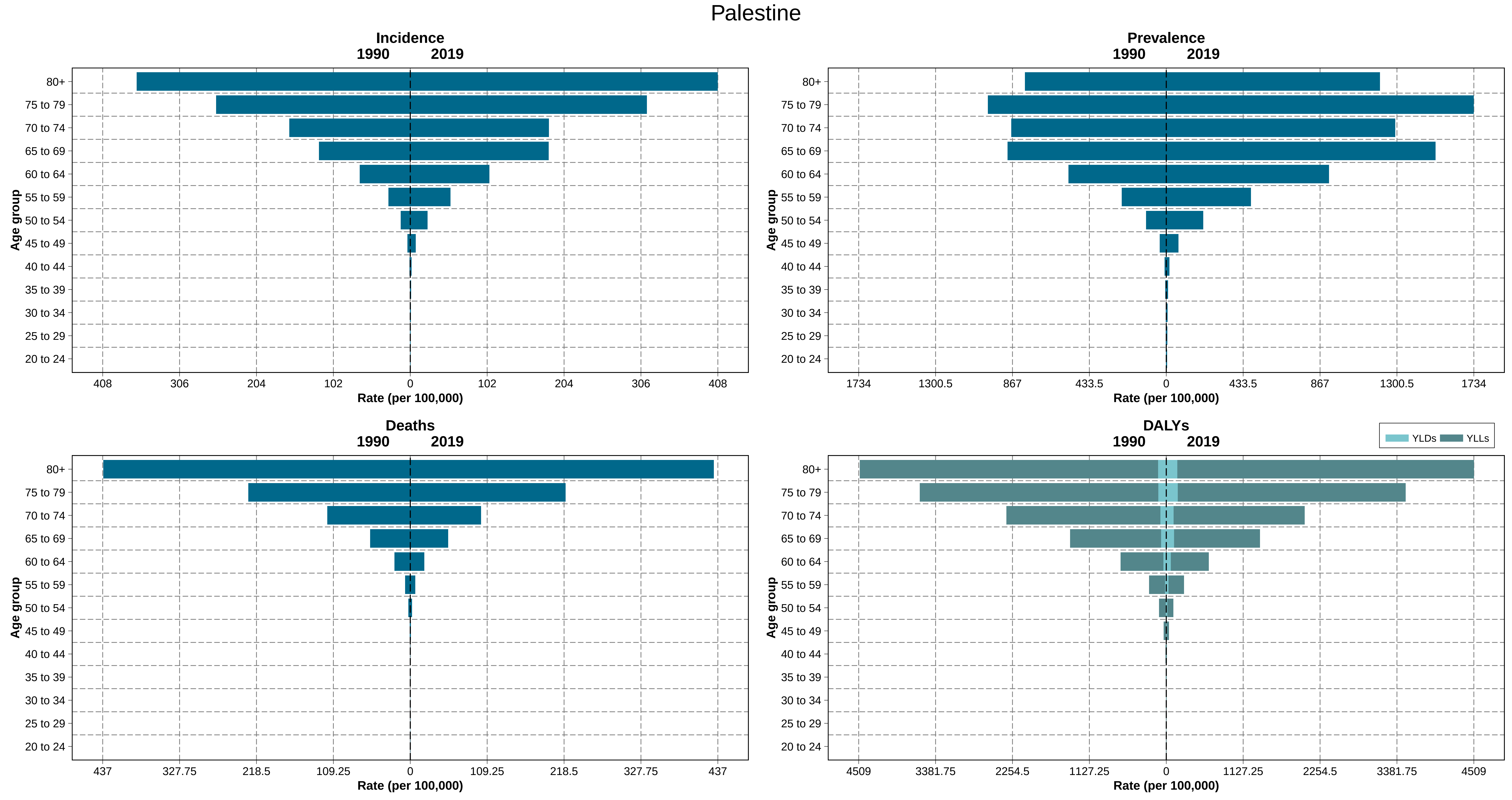
Palestine
Incidence
1990
2019
Prevalence
1990
2019
Deaths
1990
2019
DALYs
1990
2019
YLDs
YLLs
Age group
80+
75 to 79
70 to 74
65 to 69
60 to 64
55 to 59
50 to 54
45 to 49
40 to 44
35 to 39
30 to 34
25 to 29
20 to 24
408 306 204 102 0 102 204 306 408
1734 1300.5 867 433.5 0 433.5 867 1300.5 1734
437 327.75 218.5 109.25 0 109.25 218.5 327.75 437
4509 3381.75 2254.5 1127.25 0 1127.25 2254.5 3381.75 4509
Rate (per 100,000)

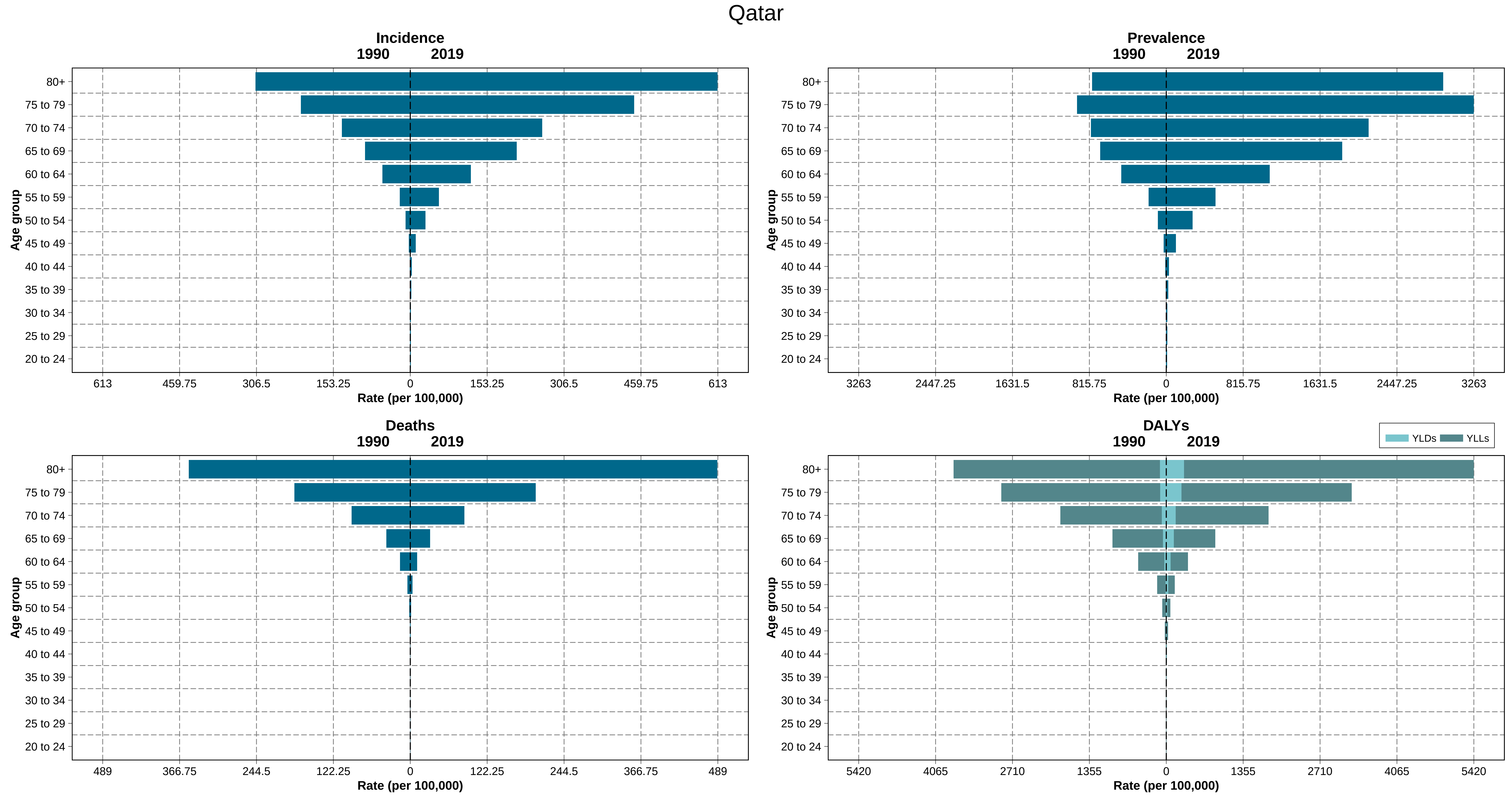
Qatar
Incidence
1990
2019
Prevalence
1990
2019
Deaths
1990
2019
DALYs
1990
2019
YLDs
YLLs
Age group
80+
75 to 79
70 to 74
65 to 69
60 to 64
55 to 59
50 to 54
45 to 49
40 to 44
35 to 39
30 to 34
25 to 29
20 to 24
613
459.75
306.5
153.25
0
3263
2447.25
1631.5
815.75
489
366.75
244.5
122.25
5420
4065
2710
1355
Rate (per 100,000)

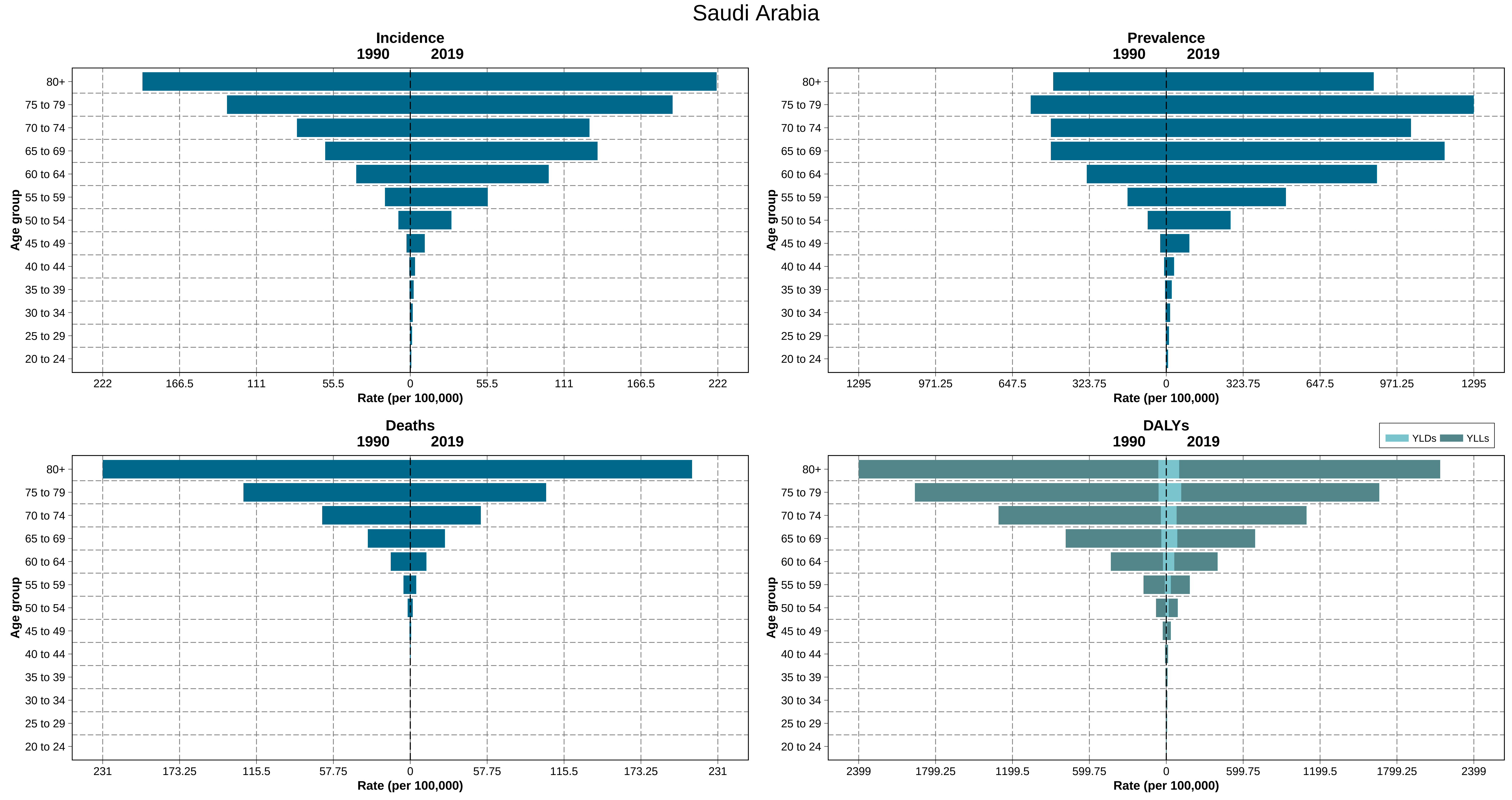

Saudi Arabia
Incidence
1990
2019
Prevalence
1990
2019
Deaths
1990
2019
DALYs
1990
2019
YLDs
YLLs
Age group
80+
75 to 79
70 to 74
65 to 69
60 to 64
55 to 59
50 to 54
45 to 49
40 to 44
35 to 39
30 to 34
25 to 29
20 to 24
222
166.5
111
55.5
0
55.5
111
166.5
222
1295
971.25
647.5
323.75
0
323.75
647.5
971.25
1295
231
173.25
115.5
57.75
0
57.75
115.5
173.25
231
2399
1799.25
1199.5
599.75
0
599.75
1199.5
1799.25
2399
Rate (per 100,000)

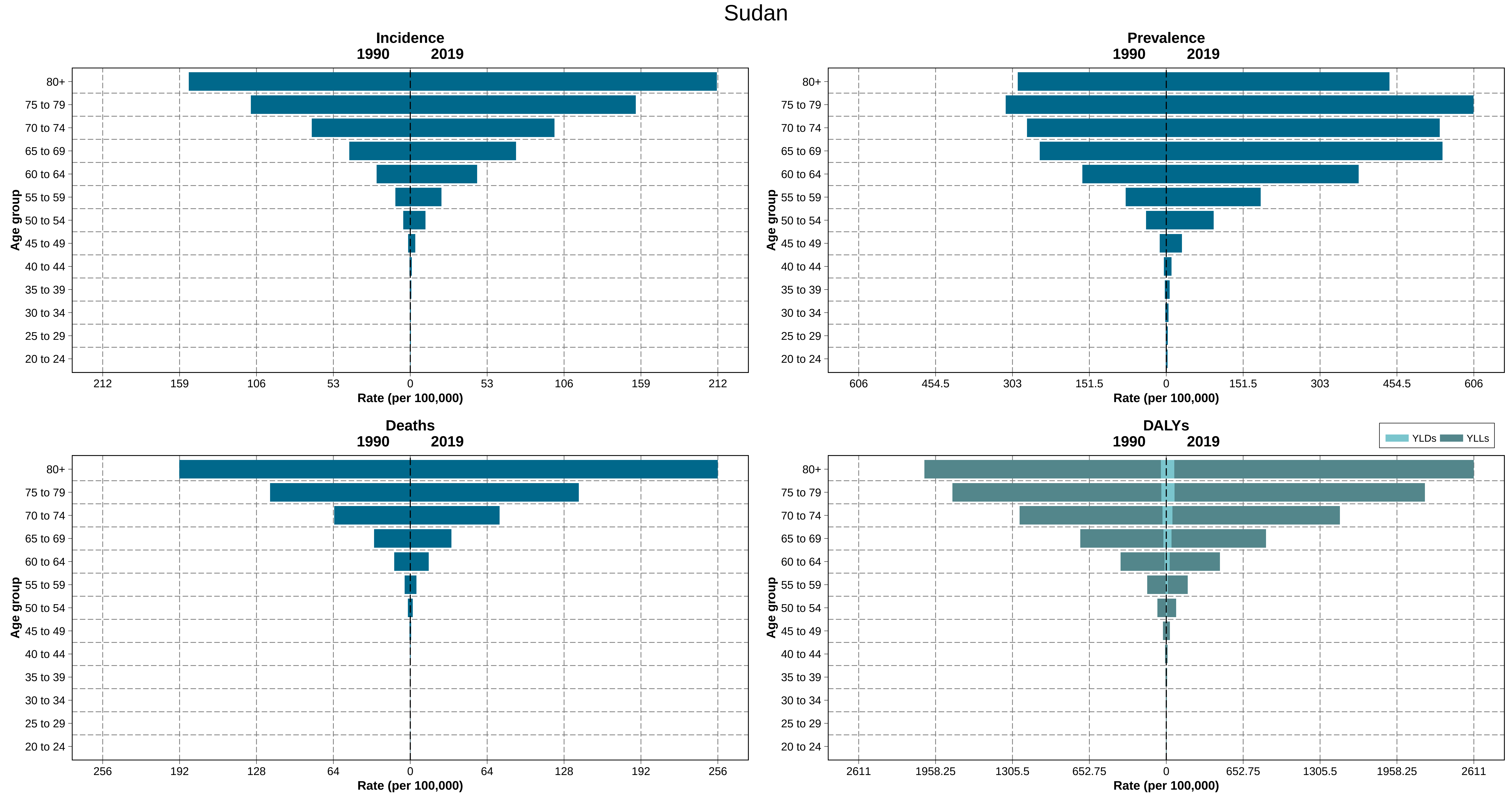

Sudan
Incidence
1990
2019
80+
75 to 79
70 to 74
65 to 69
60 to 64
55 to 59
50 to 54
45 to 49
40 to 44
35 to 39
30 to 34
25 to 29
20 to 24
Age group
212
159
106
53
0
53
106
159
212
Rate (per 100,000)
Prevalence
1990
2019
606
454.5
303
151.5
0
151.5
303
454.5
606
Rate (per 100,000)
Deaths
1990
2019
256
192
128
64
0
64
128
192
256
Rate (per 100,000)
DALYs
1990
2019
YLDs
YLLs
2611
1958.25
1305.5
652.75
0
652.75
1305.5
1958.25
2611
Rate (per 100,000)

Syrian Arab Republic

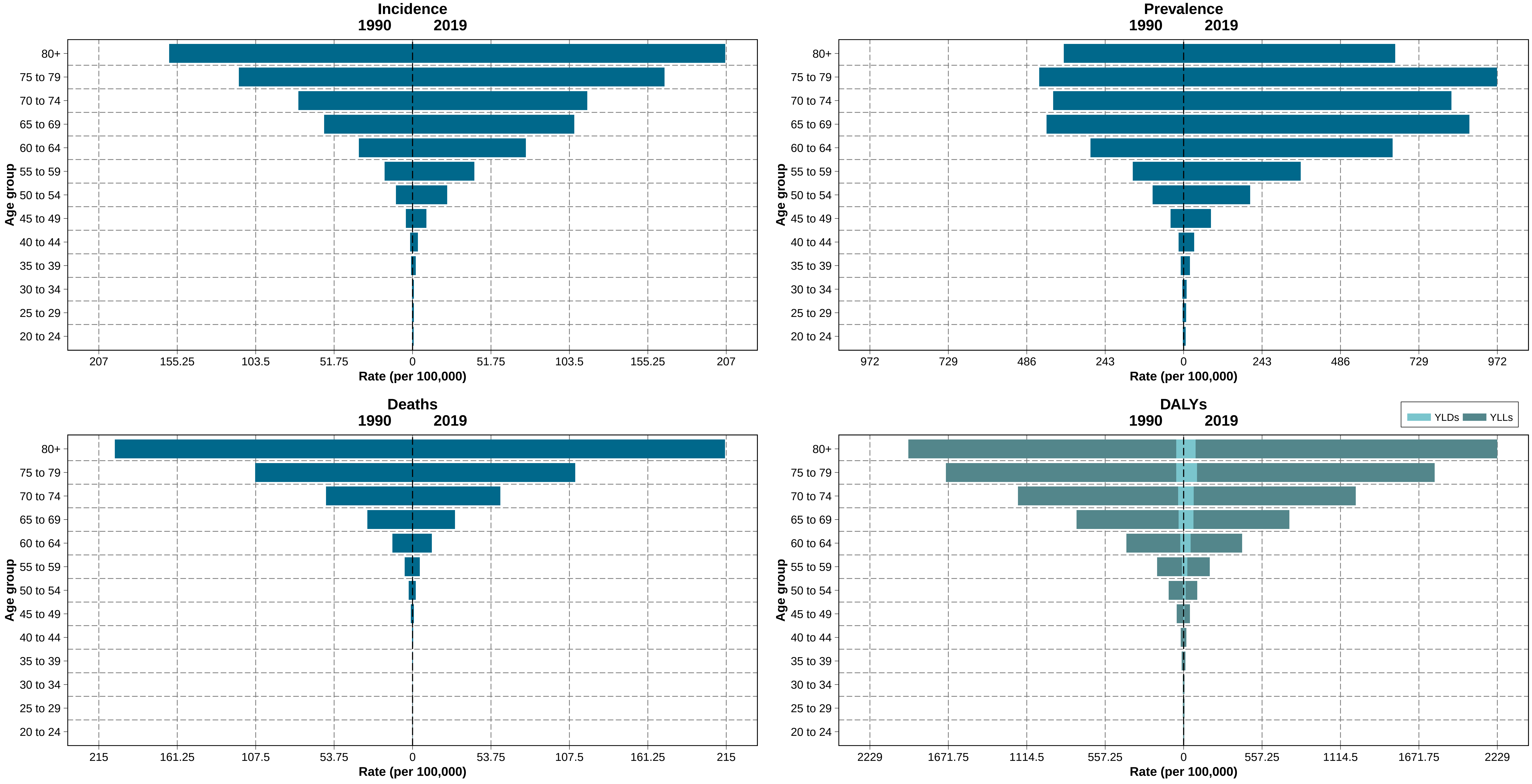

Tunisia

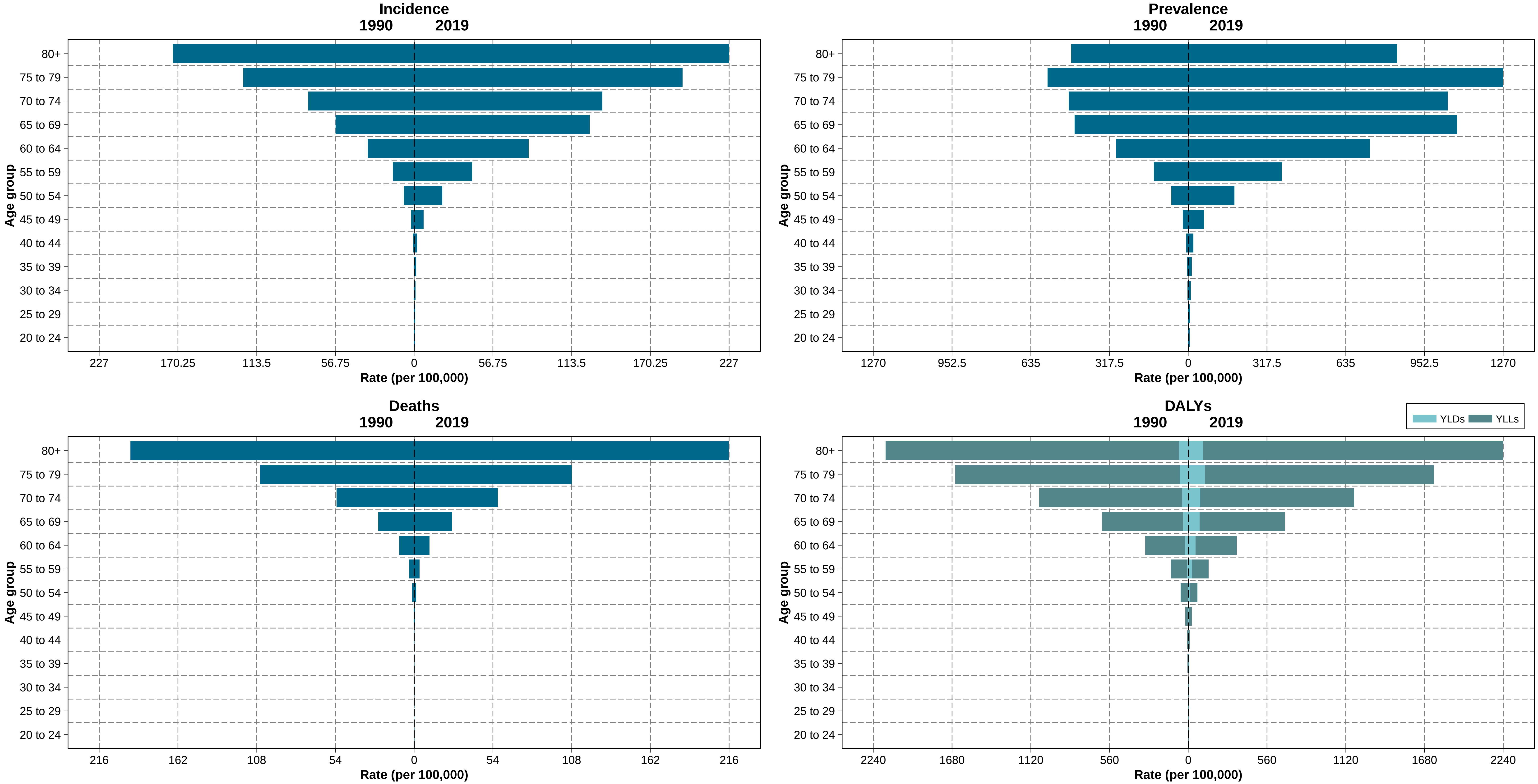

Incidence
1990
2019
80+
75 to 79
70 to 74
65 to 69
60 to 64
55 to 59
50 to 54
45 to 49
40 to 44
35 to 39
30 to 34
25 to 29
20 to 24
Age group
227
170.25
113.5
56.75
0
56.75
113.5
170.25
227
Rate (per 100,000)
Prevalence
1990
2019
1270
952.5
635
317.5
0
317.5
635
952.5
1270
Rate (per 100,000)
Deaths
1990
2019
216
162
108
54
0
54
108
162
216
Rate (per 100,000)
DALYs
1990
2019
YLDs
YLLs
2240
1680
1120
560
0
560
1120
1680
2240
Rate (per 100,000)

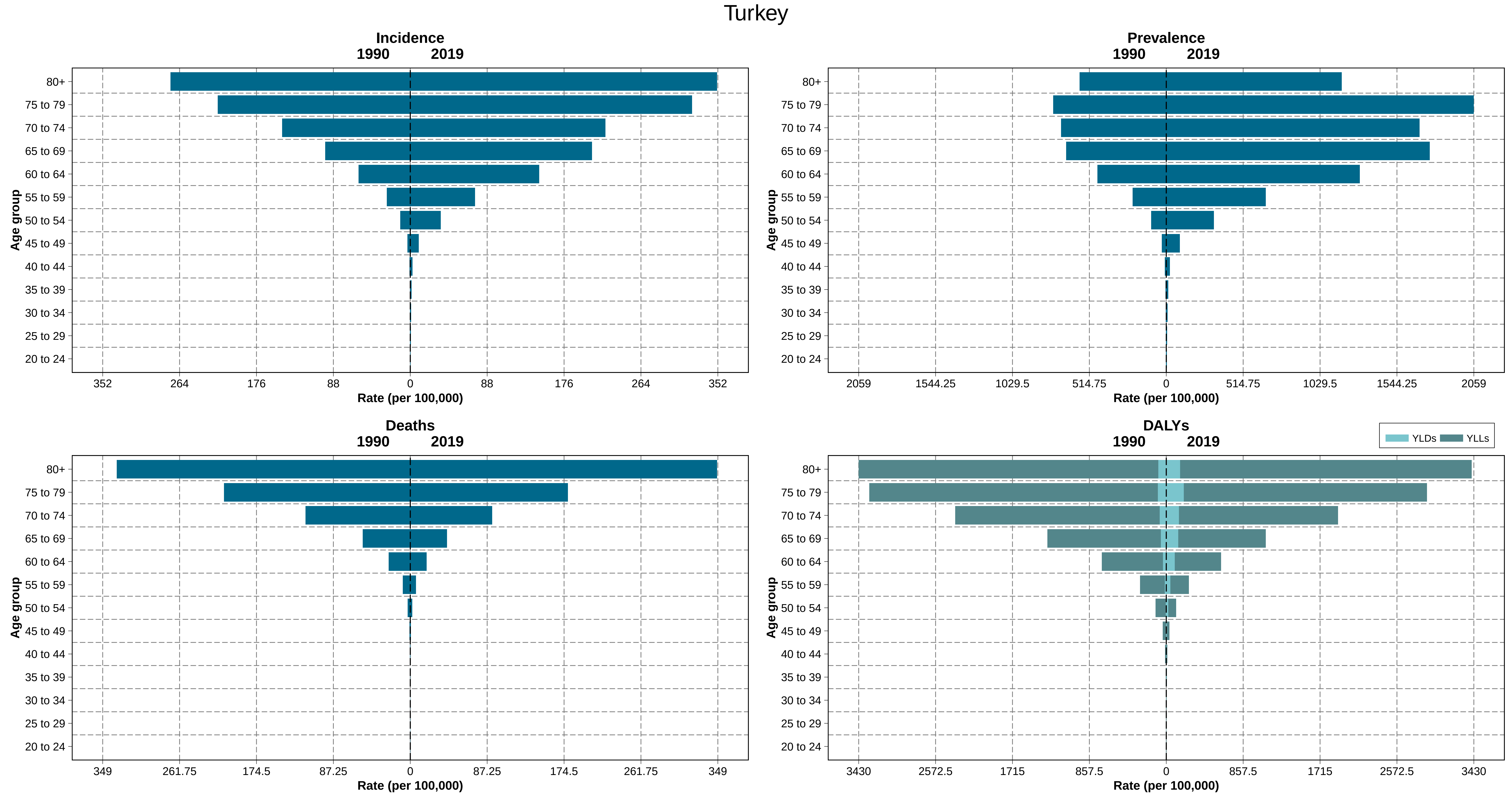

Turkey
Incidence
1990 2019
Prevalence
1990 2019
Deaths
1990 2019
DALYs
1990 2019
YLDs YLLs
Age group
80+
75 to 79
70 to 74
65 to 69
60 to 64
55 to 59
50 to 54
45 to 49
40 to 44
35 to 39
30 to 34
25 to 29
20 to 24
352 264 176 88 0 88 176 264 352
2059 1544.25 1029.5 514.75 0 514.75 1029.5 1544.25 2059
349 261.75 174.5 87.25 0 87.25 174.5 261.75 349
3430 2572.5 1715 857.5 0 857.5 1715 2572.5 3430
Rate (per 100,000)

United Arab Emirates

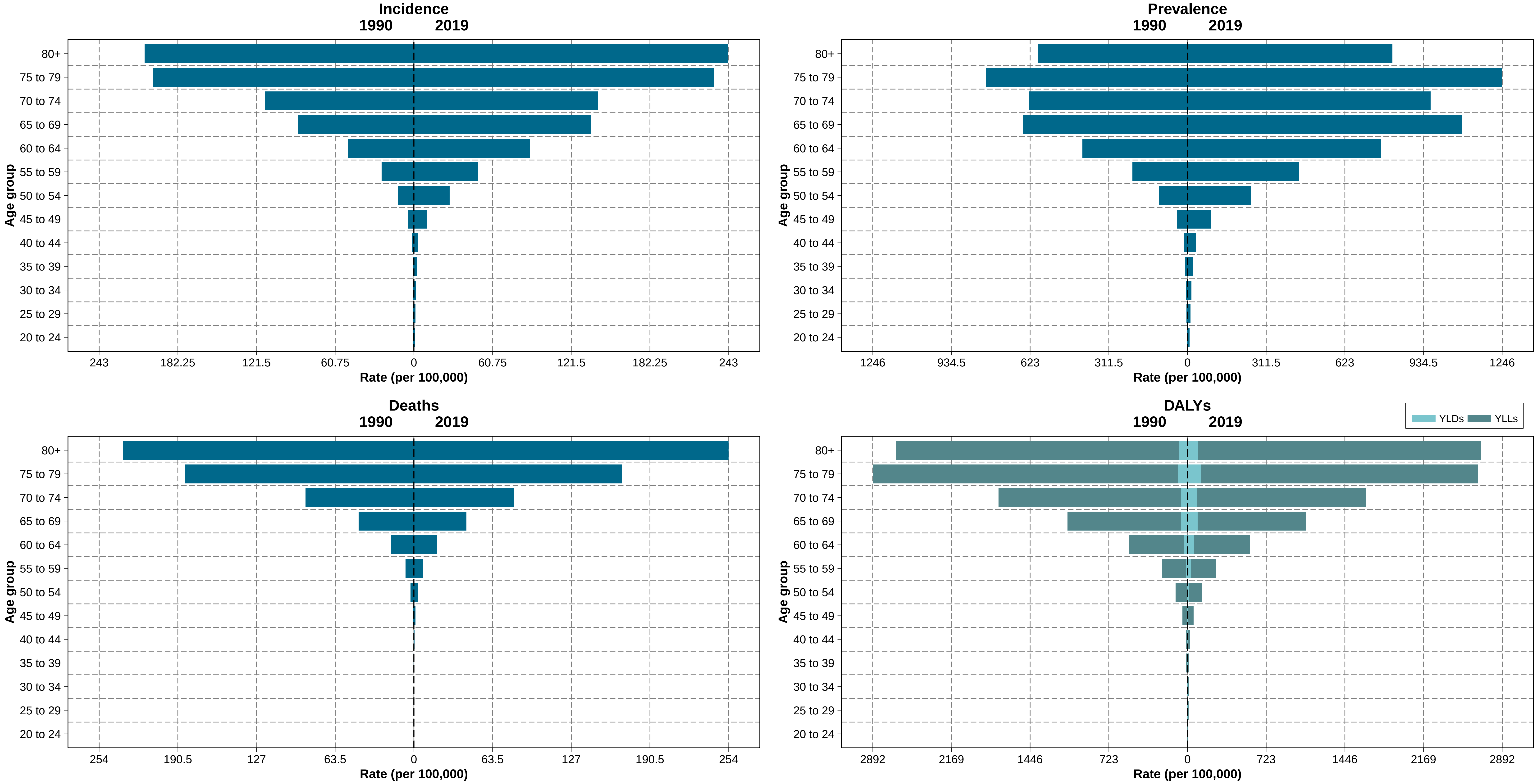

Yemen

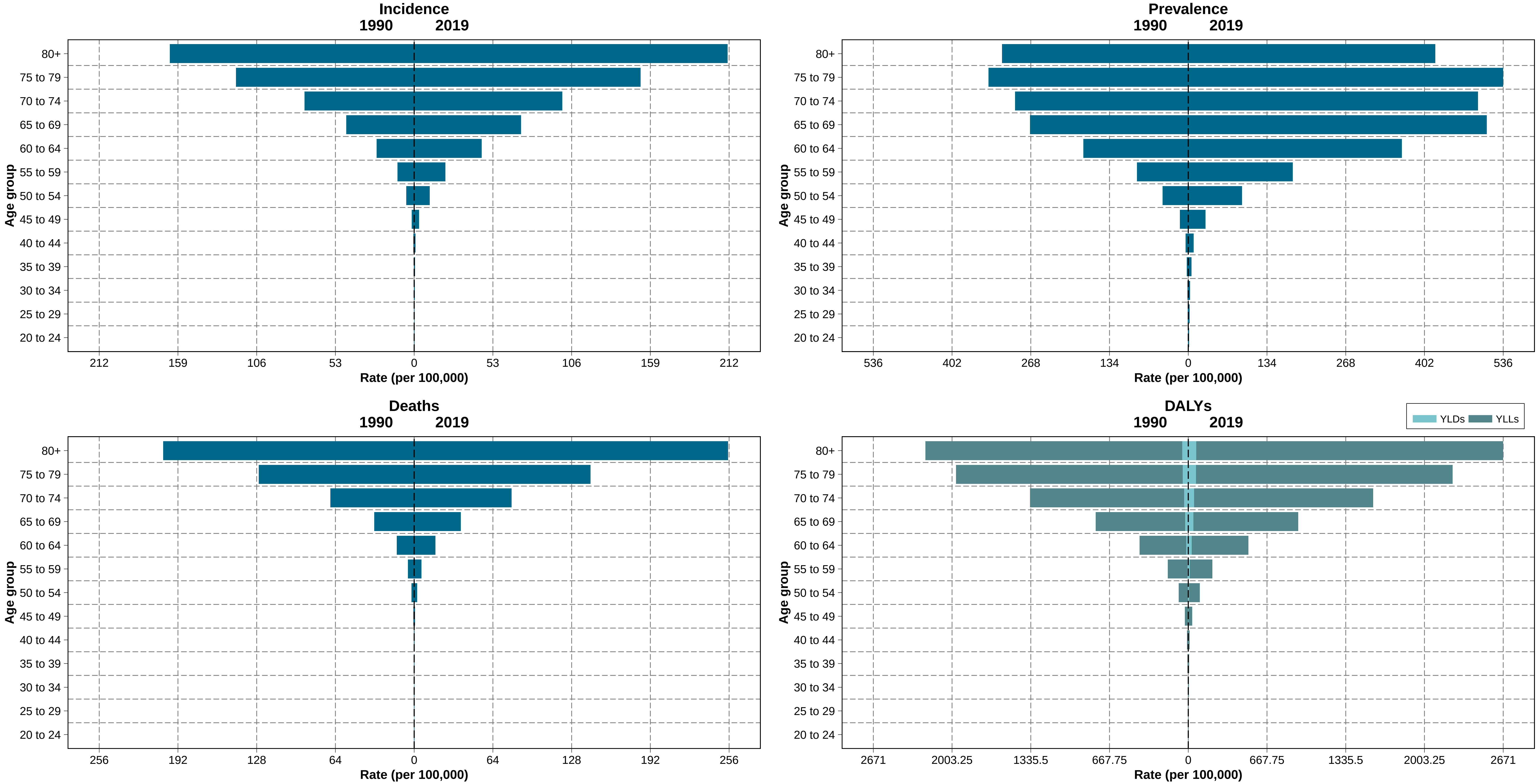

Incidence
1990
2019
Prevalence
1990
2019
Deaths
1990
2019
DALYs
1990
2019
YLDs
YLLs
Age group
80+
75 to 79
70 to 74
65 to 69
60 to 64
55 to 59
50 to 54
45 to 49
40 to 44
35 to 39
30 to 34
25 to 29
20 to 24
212 159 106 53 0 53 106 159 212
536 402 268 134 0 134 268 402 536
256 192 128 64 0 64 128 192 256
2671 2003.25 1335.5 667.75 0 667.75 1335.5 2003.25 2671
Rate (per 100,000)

Supplementary Figure 4 (a)

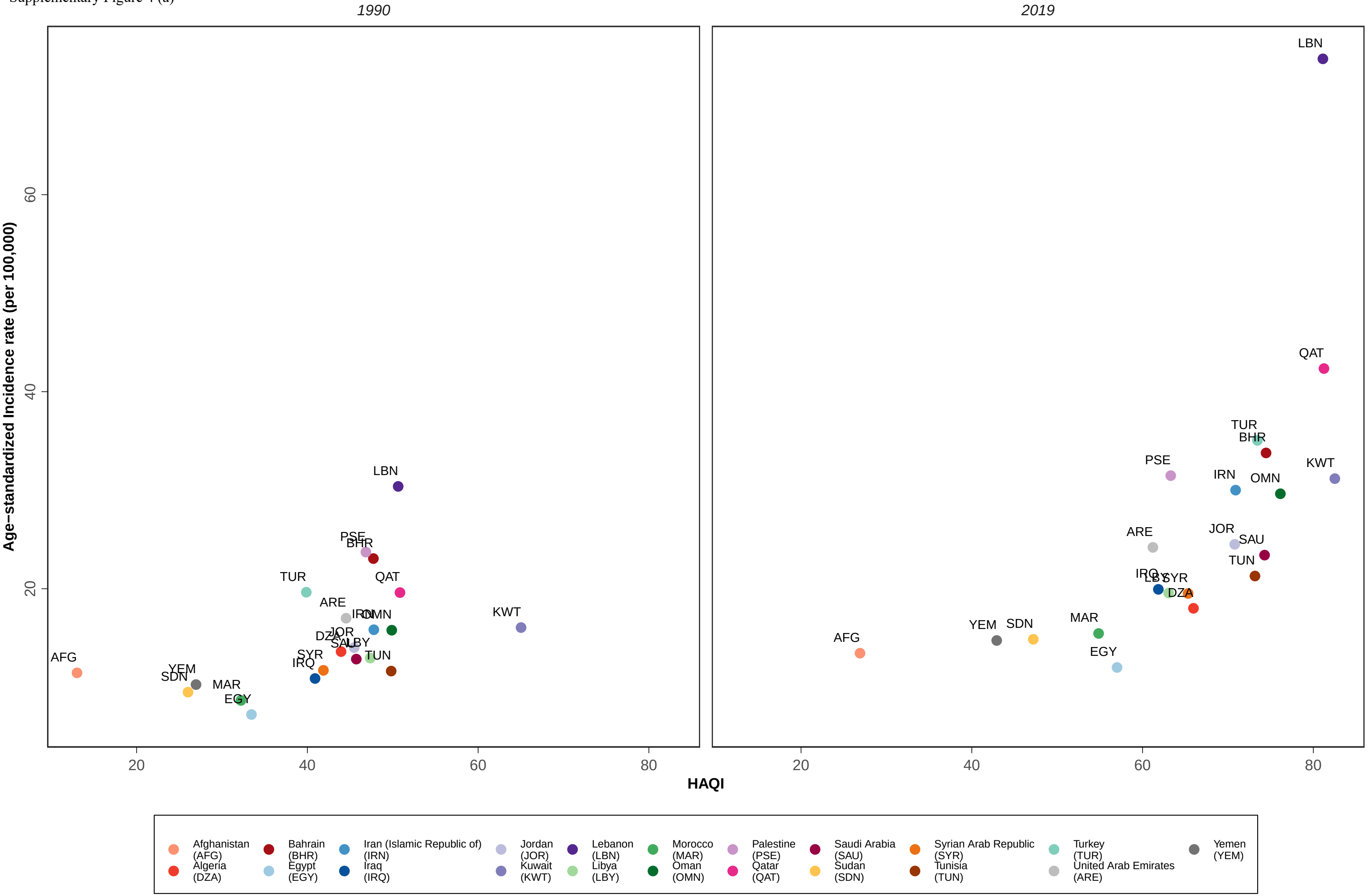

Supplementary Figure 4 (b)

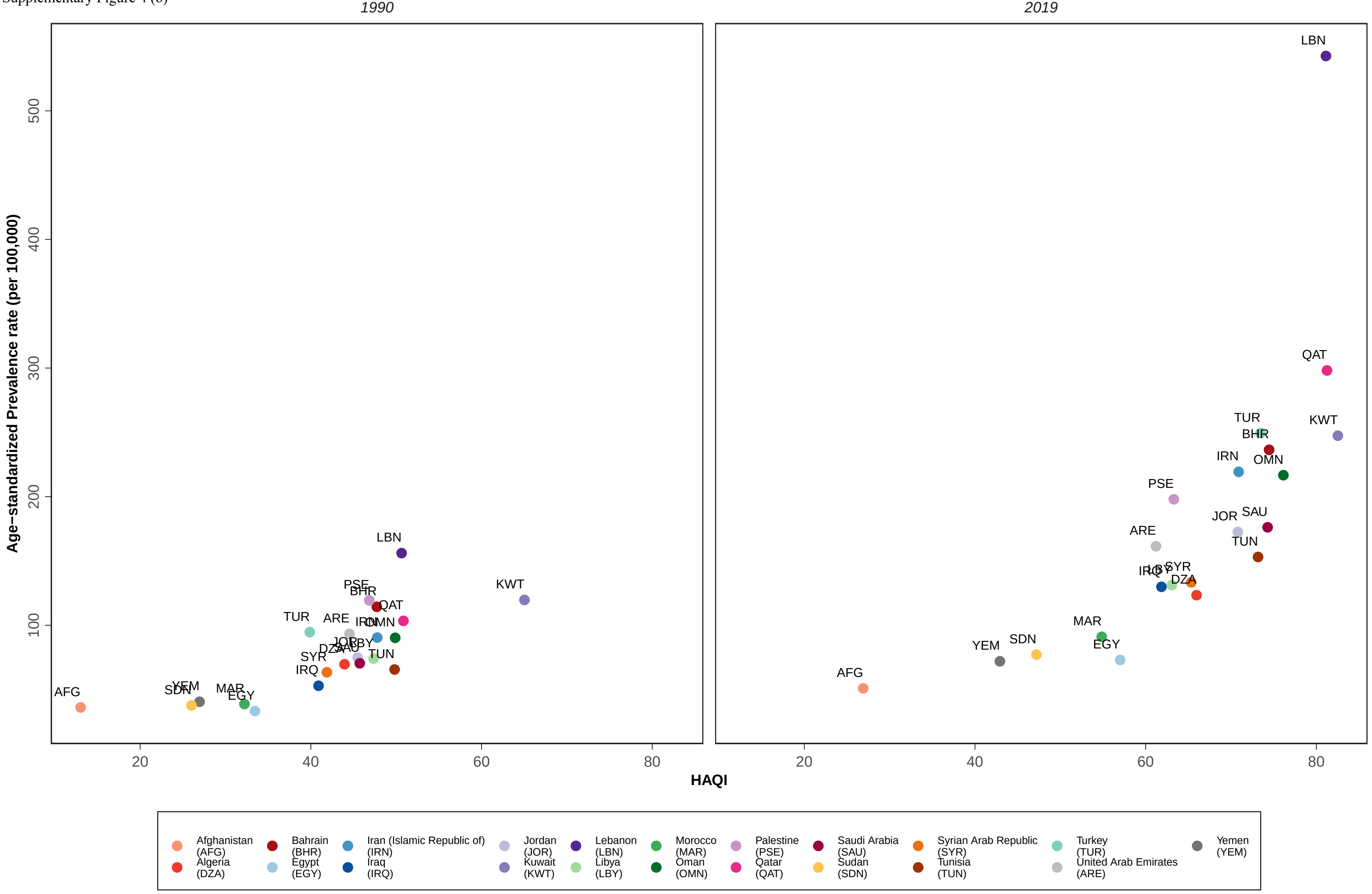

Supplementary Figure 4 (c)

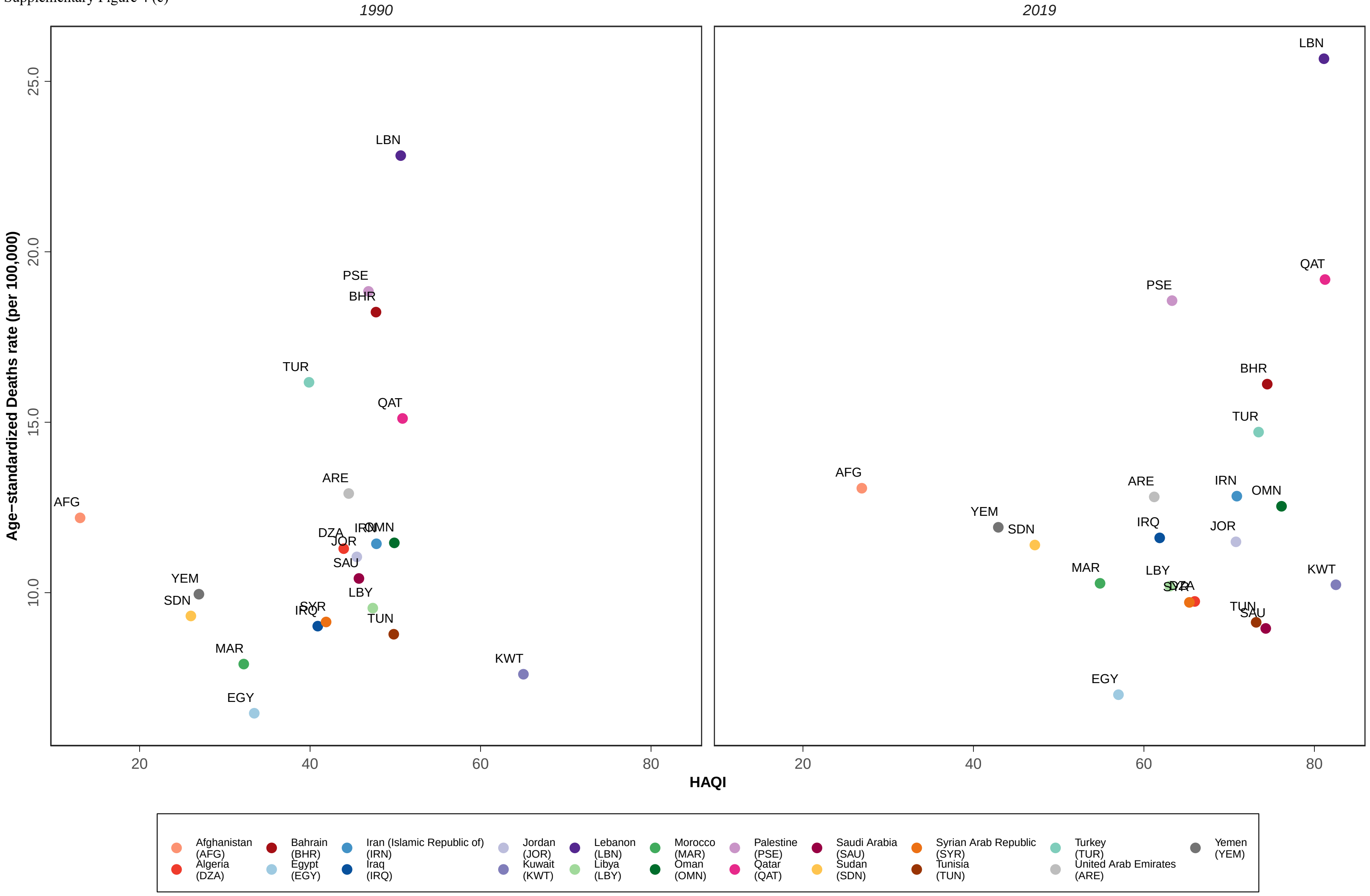

Supplementary Figure 4 (d)

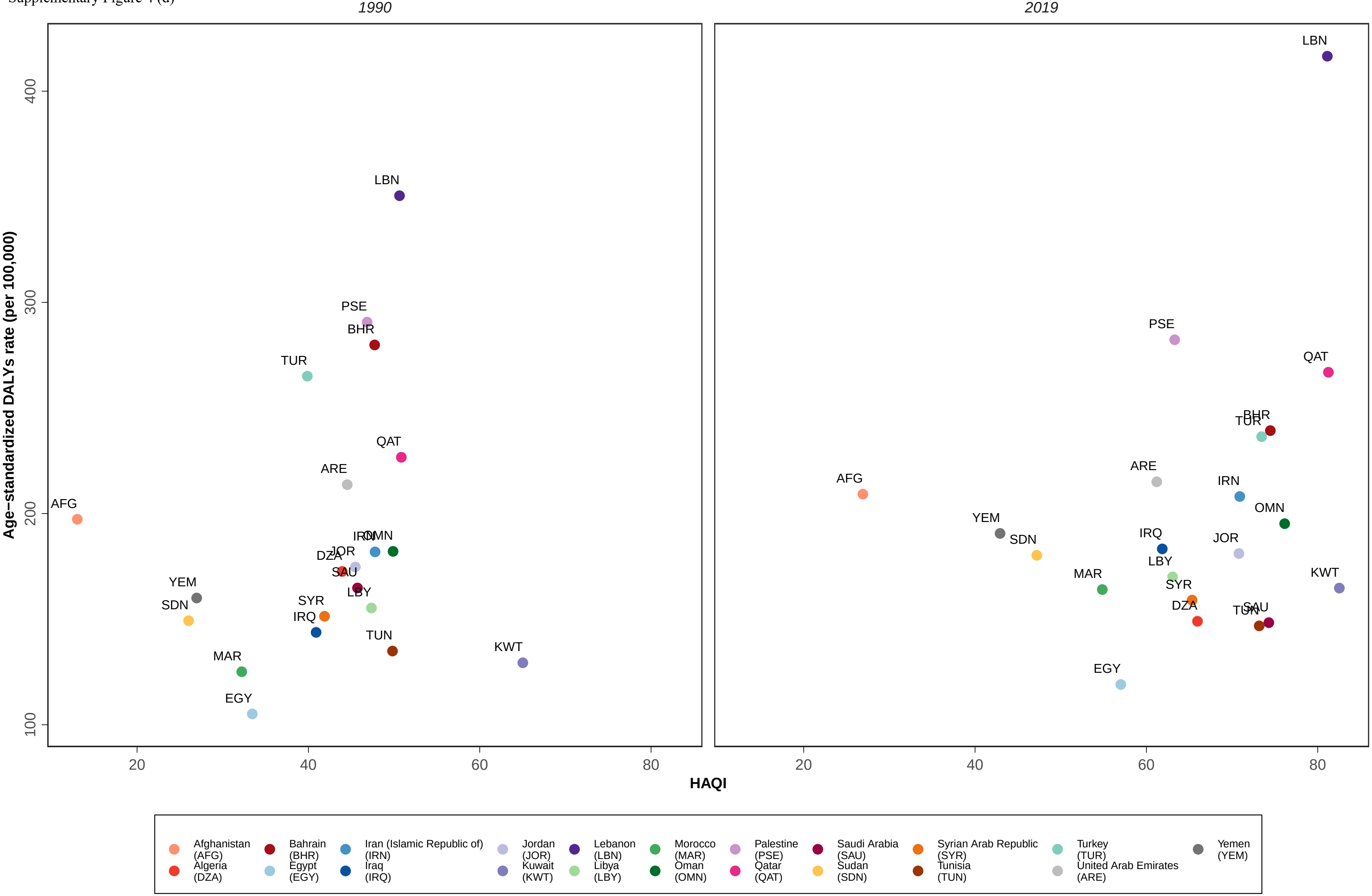

Supplement: Supplementary Table 1 — Data sources of GBD study 2019 for prostate cancer in North Africa and Middle East. [file DataSheet_1.pdf]
