# Supplementary material for: The burden of prostate cancer in North Africa and Middle East, 1990–2019: Findings from the global burden of disease study
Source: Front Oncol. 2022 Sep 13;12:961086. doi: 10.3389/fonc.2022.961086 (PMC9513750; doi:10.3389/fonc.2022.961086)
Supplement: Supplementary file 2 [file DataSheet_2.docx]

## Authors’ Contributions

Contributions to be included in appendix at the above location:

### Providing data or critical feedback on data sources

Mohsen Abbasi-Kangevari, Hassan Abidi, Sajjad Ahmad, Ali Ahmadi, Sepideh Ahmadi, Hanadi Al Hamad, Syed Aljunid, Jalal Arabloo, Seyyed Shamsadin Athari , Amirhossein Azari Jafari, Sara Bagherieh, Akshaya Bhagavathula, Ahmad Daryani, Mostafa Dianatinasab, Farshad Farzadfar, Ali Fatehizadeh, Seyyed-Hadi Ghamari, Pouya Goleij, Nima Hafezi-Nejad, Arvin Haj-Mirzaian, Shafiul Haque, Mowafa Househ, Amirreza Javadi Mamaghani, Farahnaz Joukar, Rohollah Kalhor, Yousef Khader, Ali-Asghar Kolahi, Somayeh Livani, Ahmad Mafi, Ata Mahmoodpoor, Fariborz Mansour-Ghanaei, Entezar Mehrabi Nasab, Seyyedmohammadsadeq Mirmoeeni, Ali H Mokdad, Mohsen Naghavi, Mohammadreza Naghipour, Zuhair Natto, Zahra Zahid Piracha, Alireza Rafiei, Mohammad Sadegh Razeghinia, Umar Saeed, Sahar Saeedi Moghaddam, Maryam Sahebazzamani, Abdallah M Samy, Brijesh Sathian, Masood Ali Shaikh, Sara Sheikhbahaei, Irfan Ullah, Sahel Valadan Tahbaz, Hossein Yahyazadeh, Fereshteh Yazdanpanah, Deniz Yuce, Iman Zare, and Mohammad Zoladl.

### Developing methods or computational machinery

Ali H Mokdad, Mohsen Naghavi, and Sahar Saeedi Moghaddam. .

### Providing critical feedback on methods or results

Behzad Abbasi, Mohsen Abbasi-Kangevari, Hassan Abidi, Muhammad Sohail Afzal, Sajjad Ahmad, Ali Ahmadi, Haroon Ahmed, Mostafa Akbarzadeh-Khiavi, Hanadi Al Hamad, Fadwa Alhalaiqa, Yousef Alimohamadi, Syed Aljunid, Omar Almidani, Alireza Ansari-Moghaddam, Jalal Arabloo, Morteza Arab-Zozani, Seyyed Shamsadin Athari , Sina Azadnajafabad, Mohammadreza Azangou-Khyavy, Amirhossein Azari Jafari, Nayereh Baghcheghi, Sara Bagherieh, Abdul-Monim Batiha, Akshaya Bhagavathula, Ali Bijani, Nadeem Shafique Butt, Ahmad Daryani, Mostafa Dianatinasab, Iman El Sayed, Muhammed Elhadi, Farshad Farzadfar, Ali Fatehizadeh, Masood Fereidoonnezhad, Masoud Foroutan, Seyyed-Hadi Ghamari Maryam Gholamalizadeh, Mohamad Golitaleb, Mohammed I M Gubari, Nima Hafezi-Nejad, Samer Hamidi, Shafiul Haque, khezar hayat, Mohammad-Salar Hosseini, Mowafa Househ, Elham Jamshidi, Hamidreza Jamshidi, Farahnaz Joukar, Ali Kabir, Rohollah Kalhor, Amirali Karimi, Yousef Khader, Javad Khanali, Behzad Kiani, Hamid Reza Koohestani, Bagher Larijani, Somayeh Livani, Farzan Madadizadeh, Ahmad Mafi, Ata Mahmoodpoor, Keivan Majidzadeh-A, Mohammad-Reza Malekpour, Reza Malekzadeh, Ahmad Azam Malik, Fariborz Mansour-Ghanaei, Seyed Farzad Maroufi, Entezar Mehrabi Nasab, Seyyedmohammadsadeq Mirmoeeni, Yousef Mohammad, Esmaeil Mohammadi, Saeed Mohammadi, Abdollah Mohammadian-Hafshejani, Ali H Mokdad, Sara Momtazmanesh, Rahmatollah Moradzadeh, Mohsen Naghavi, Mohammadreza Naghipour, Zuhair Natto, Seyed Aria Nejadghaderi, Maryam Noori, Ali Nowroozi, Hassan Okati-Aliabad, Zahra Zahid Piracha, Faheem Hyder Pottoo, Alireza Rafiei, Vahid Rahmanian, Araz Ramazan Ahmad, Mahsa Rashidi, Mohammad-Mahdi Rashidi, Mohammad Sadegh Razeghinia, Nazila Rezaei, Negar Rezaei, Mohsen Rezaeian, Umar Saeed, Sahar Saeedi Moghaddam, Maryam Sahebazzamani, Abdallah M Samy, Muhammad Arif Nadeem Saqib, Brijesh Sathian, Sadaf Sepanlou, Saeed Shahabi, Masood Ali Shaikh, Sara Sheikhbahaei, Reza Shirkoohi, Parnian Shobeiri, Muhammad Suleman, Amir Tiyuri, Irfan Ullah, Faezeh Vakhshiteh, Sahel Valadan Tahbaz, Hossein Yahyazadeh, Fereshteh Yazdanpanah, Deniz Yuce, Mazyar Zahir, and Maryam Zamanian.

### Drafting the work or revising is critically for important intellectual content

Behzad Abbasi, Mohsen Abbasi-Kangevari, Hassan Abidi, Eman Abu-Gharbieh, Muhammad Sohail Afzal, Sajjad Ahmad, Ali Ahmadi, Sepideh Ahmadi, Mostafa Akbarzadeh-Khiavi, Hamed Akhavizadegan, Fadwa Alhalaiqa, Omar Almidani, Erfan Amini, Jalal Arabloo, Morteza Arab-Zozani, Sina Azadnajafabad, Mohammadreza Azangou-Khyavy, Amirhossein Azari Jafari, Nader Bagheri, Sara Bagherieh, Nadeem Shafique Butt, Reza Darvishi Cheshmeh Soltani, Ahmad Daryani, Mostafa Dianatinasab, Iman El Sayed, Muhammed Elhadi, Farshad Farzadfar, Ali Fatehizadeh, Masoud Foroutan, Seyyed-Hadi Ghamari, Mohamad Golitaleb, Nima Hafezi-Nejad, Shafiul Haque, Khezar Hayat, Mohammad-Salar Hosseini, Mowafa Househ, Mohammed I M Gubari, Ali Kabir, Yousef Khader, Javad Khanali, Bagher Larijani, Ata Mahmoodpoor, Mohammad-Reza Malekpour, Reza Malekzadeh, Ahmad Azam Malik, Seyyedmohammadsadeq Mirmoeeni, Yousef Mohammad, Abdollah Mohammadian-Hafshejani, Ali H Mokdad, Sara Momtazmanesh, Paula Moraga, Mohsen Naghavi, Zuhair Natto, Seyed Aria Nejadghaderi, Ali Nowroozi, Reza Pakzad, Zahra Zahid Piracha, Araz Ramazan Ahmad, Mohammad Sadegh Razeghinia, Nazila Rezaei, Negar Rezaei, Umar Saeed, Sahar Saeedi Moghaddam, Maryam Sahebazzamani, Amirhossein Sahebkar, Abdallah M Samy, Sadaf Sepanlou, Saeed Shahabi, Sara Sheikhbahaei, Parnian Shobeiri, Irfan Ullah, Sahel Valadan Tahbaz, Hossein Yahyazadeh, Deniz Yuce, Mazyar Zahir, Maryam Zamanian, Iman Zare, and Mohammad Zoladl.

### Managing the overall research enterprise

Ali H Mokdad and Mohsen Naghavi.
